# DNV-TC: a developmental network vulnerability-based classification system for precision oncology

**DOI:** 10.3389/fcell.2026.1780827

**Published:** 2026-09-09

**Authors:** Draga Toncheva, Vassil Sgurev

**Affiliations:** 1 Department of Biological Sciences, Bulgarian Academy of Sciences, Sofia, Bulgaria; 2 Institute of Information and Communication Technologies, Bulgarian Academy of Sciences, Sofia, Bulgaria

**Keywords:** cancer stem cells, developmental network vulnerability, gene regulatory networks, network medicine, precision oncology, systems biology, targeted therapy, tumor classification

## Abstract

**Introduction:**

Current tumor classification systems based on histology and molecular subtypes inadequately capture the functional complexity of cancer biology. Tumors frequently reactivate embryonic developmental programs, becoming dependent on master regulators that govern pluripotency, differentiation and tissue morphogenesis. This developmental reactivation creates architectural vulnerabilities in gene regulatory networks that can be therapeutically exploited.

**Methods:**

We propose DNV-TC, a four-dimensional classification system that stratifies tumors according to the reactivated developmental program, network architectural vulnerability, cascade expansion phenotype and metastatic propensity. The system integrates developmental biology with network medicine through three quantitative indices computed on multi-layer gene regulatory networks: the tumor developmental regulatory impact (T-DRI), tumor disease network vulnerability (TD-NV) and cascade expansion index-tumor (CEI-T). DNV-TC was applied retrospectively to representative tumor types using published molecular and clinical data.

**Results:**

Tumors with high network vulnerability showed marked responses to targeted therapies (87% response rate) but rapidly developed resistance (median 6–8 months), while tumors with reactivated pluripotency programs displayed cancer stem-cell characteristics and therapeutic resistance. Network vulnerability class retained independent prognostic value after adjustment for stage, grade and age (HR 2.3, 95% CI 1.8–2.9, p < 0.001), and metastatic propensity class outperformed TNM staging for 5-year metastasis-free survival prediction (AUC 0.842 versus 0.712, p < 0.001).

**Discussion:**

DNV-TC provides a mechanistically grounded classification system that bridges developmental biology and precision oncology, thus enabling rational selection of therapeutic targets and combination strategies. The present analyses are retrospective and in silico, and prospective external validation is required.

## 1 Introduction

The classification of human malignancies has evolved from purely morphological descriptions to sophisticated molecular characterizations. Traditional histopathology remains clinically essential but possesses fundamental limitations. Tumors with identical histological appearance frequently exhibit markedly different clinical behaviors, treatment responses, and outcomes. This phenotypic heterogeneity masked by morphological similarity reflects the underlying biological diversity that histology alone cannot capture. High-throughput molecular profiling promised to resolve this ambiguity. The Cancer Genome Atlas and similar initiatives have cataloged somatic mutations, copy number alterations, gene expression patterns, and epigenetic modifications across tens of thousands of tumors (Weinstein et al., 2013). These efforts have identified recurrent driver mutations, defined molecular subtypes, and established prognostic gene expression signatures. Breast cancer is now classified into luminal A, luminal B, HER2-enriched, and basal-like subtypes (Perou et al., 2000; Sørlie et al., 2001). Colorectal cancers are stratified into consensus molecular subtypes (Guinney et al., 2015). These molecular classifications correlate more strongly with outcomes than histology alone. Yet, molecular classification systems exhibit significant limitations. They remain predominantly descriptive, cataloging alterations without fully explaining why those alterations produce specific phenotypes. Two tumors may harbor different mutations but exhibit similar biology because the mutations converge on common pathways. Conversely, identical mutations may produce different outcomes depending on the cellular context. Current classifications struggle with intra-tumoral heterogeneity and do not adequately capture the functional architecture of tumor regulatory networks. A conceptually powerful framework was developed by recognizing that malignant transformation frequently involves reactivation of embryonic developmental programs (Ben-Porath et al., 2008). During embryogenesis, cells must proliferate extensively, migrate long distances, invade through tissue barriers, survive in foreign microenvironments, and dynamically interconvert between phenotypic states. These capabilities are precisely the aberrant behaviors characterizing malignancy (Hanahan and Weinberg, 2011). The hypothesis that cancer represents inappropriate reactivation of developmental programs in adult tissues provides an elegant explanation for why tumors possess coordinated capabilities rather than random collections of unrelated defects. Extensive molecular evidence supports developmental reactivation. Transcription factors serving as master regulators of embryonic cell fate decisions are frequently reactivated in cancer. SOX2, which is essential for maintaining pluripotency in embryonic stem cells, becomes aberrantly expressed in glioblastoma, lung squamous cell carcinoma, and esophageal cancer, promoting cancer stem-cell properties and therapeutic resistance (Bass et al., 2009; Gangemi et al., 2009; Sell, 2004; Bao et al., 2006; Piccirillo et al., 2006). The HOXA gene cluster shows ectopic reactivation in acute myeloid leukemia, where HOXA9 overexpression blocks myeloid differentiation and drives leukemogenesis (Collins and Hess, 2016). MYCN, which is critical for neural crest development, is amplified in neuroblastoma and small-cell lung cancer (Brodeur, 2003). The neural crest transcriptional network is systematically reactivated in melanoma, driving invasive and metastatic phenotypes mirroring normal neural crest migration (Rambow et al., 2018). Developmental signaling pathways show pervasive reactivation. The Wnt-beta-catenin pathway is hyperactivated in colorectal cancer, hepatocellular carcinoma, and medulloblastoma (Polakis, 2012). Hedgehog signaling becomes constitutively active in basal cell carcinoma and medulloblastoma (Northcott et al., 2019). Notch signaling shows gain-of-function mutations in T-cell acute lymphoblastic leukemia (Van Vlierberghe and Ferrando, 2012). Epithelial–mesenchymal transition is recapitulated in carcinoma metastasis through the reactivation of TWIST, SNAIL, and ZEB transcription factors (Thiery et al., 2009). This developmental reactivation exhibits striking specificity. Tumors predominantly reactivate programs that are relevant to their cell of origin. Gliomas reactivate neural stem-cell programs, leukemias reactivate hematopoietic stem-cell programs, and sarcomas reactivate mesenchymal developmental programs. This lineage-specificity indicates that developmental gene regulatory networks remain latent in differentiated adult tissues, where they remain epigenetically silenced but structurally intact and are capable of reactivation when the repressive barriers are breached. Understanding cancer as developmental program reactivation necessitates a systems-level analytical framework. Network medicine was developed by applying concepts from graph theory, dynamical systems theory, and complex systems science to biological networks (Barabási et al., 2011; Silverman and Loscalzo, 2012; Creixell et al., 2015; Lefebvre et al., 2010). Cells are viewed as highly interconnected regulatory networks, where genes, proteins, and metabolites function as nodes with regulatory relationships constituting edges. The network structure exhibits non-random organizational principles, profoundly influencing function. Many biological networks show scale-free topology with highly connected hub nodes and many sparsely connected peripheral nodes (Barabási and Oltvai, 2004). This architecture confers robustness to random perturbations but creates vulnerabilities when critical hubs are disrupted. Gene regulatory networks exhibit hierarchical modularity organized into functional modules executing specific biological programs (Hartwell et al., 1999). Recurrent network motifs serve as basic computational units (Alon, 2007). The concept of attractor states from the dynamical systems theory provides a powerful lens for understanding cell fate (Huang et al., 2009). Different cell types can be conceptualized as distinct attractors in high-dimensional gene expression space. Waddington visualized this as an epigenetic landscape where the cellular state rolls downward into valleys representing stable differentiated cell types (Waddington, 1957; Huang, 2012). During development, cells progress from pluripotent states through increasingly restricted progenitor states, ultimately settling into terminally differentiated attractors. If tumors are aberrant attractor states arising from developmental network reactivation, what makes these pathological attractors stable and vulnerable? Not all network nodes are equally important. Some genes can be deleted with minimal phenotypic consequence, while the loss of others is catastrophic. This differential importance reflects network vulnerability, which is the degree to which the system function depends on individual components. The evolutionary constraint provides one indicator since highly conserved genes that are intolerant of loss-of-function variation likely occupy critical network positions (Lek et al., 2016). Structural measures from graph theory quantify the topological importance through centrality metrics (Koschützki and Schreiber, 2008). In cancer, network vulnerability is of particular significance. When tumors reactivate developmental programs, they become dependent on the master regulators of those programs. A glioblastoma reactivating *SOX2*-driven neural stem-cell programs becomes addicted to *SOX2* (Gangemi et al., 2009). A melanoma reactivating neural crest programs becomes dependent on *SOX10*, *MITF*, and the associated factors (Rambow et al., 2018). This dependency represents therapeutic vulnerability. Oncogene addiction has been validated clinically (Weinstein, 2002). Chronic myeloid leukemia driven by *BCR-ABL* responds remarkably to imatinib (Druker et al., 2001; O'Brien et al., 2003). *EGFR*-mutant lung adenocarcinomas respond strongly to EGFR tyrosine kinase inhibitors (Lynch et al., 2004). *BRAF*-mutant melanomas initially respond to BRAF inhibitors (Chapman et al., 2011). These responses occur because tumor networks have been architecturally reorganized around oncogenic drivers, creating single-point vulnerabilities. The current tumor classification systems inadequately capture functional network architecture. Histology describes the morphology. Molecular subtypes catalog mutations and expression patterns. Neither directly assesses which developmental programs are reactivated, how regulatory networks are rewired, or where architectural vulnerabilities reside. The DNV-TC framework addresses these requirements through multi-dimensional classification grounded in quantitative network analysis. DNV-TC classifies tumors along four orthogonal axes identifying which developmental programs are reactivated, quantifying network architectural vulnerability, characterizing cascade expansion phenotype, and predicting metastatic propensity. This classification is operationalized through three quantitative indices derived from multi-layer network analysis, namely, the tumor developmental regulatory impact measuring gene participation in reactivated developmental programs, tumor disease network vulnerability quantifying structural criticality, and cascade expansion index measuring perturbation propagation through networks. This study presents the DNV-TC framework and demonstrates its utility through proof-of-concept analysis of well-characterized tumor types. Our aims are to: (1) develop mathematical formulations for core indices, (2) define the four-dimensional classification system, (3) apply DNV-TC to representative tumors using published data, (4) demonstrate correlations with clinical outcomes, and (5) discuss therapeutic implications. We anticipate that DNV-TC will provide a mechanistically grounded classification system bridging molecular characterization and therapeutic decision-making.

## 2 Materials and methods

### 2.1 Conceptual framework

DNV-TC is grounded in a multi-layer representation of tumor gene regulatory networks capturing hierarchical organization and cross-layer interactions. We conceptualize tumor networks as comprising four interconnected layers, namely, signaling, transcriptional, epigenetic, and metabolic. Each layer contains nodes representing genes, proteins, or regulatory elements with edges denoting the regulatory relationships. Formally, we represent multi-layer networks through supra-adjacency matrices encoding both the intra-layer connections within single regulatory layers and inter-layer connections representing cross-talk between layers. This representation enables the computation of multi-layer centrality measures accounting for gene importance across all regulatory contexts simultaneously. The developmental reactivation hypothesis posits that tumorigenesis involves systematic network rewiring toward configurations recapitulating embryonic developmental programs. Normal adult tissues maintain stable differentiated states through epigenetic silencing of the developmental master regulators and checkpoint mechanisms preventing inappropriate proliferation. Cancer-associated mutations and epigenetic alterations erode these barriers, thus enabling reactivation of genes such as *SOX2, HOXA9,* or *MYCN* orchestrating embryonic self-renewal, lineage specification, or morphogenesis. Once reactivated, these developmental regulators reorganize tumor networks around themselves, creating architectural dependencies constituting therapeutic vulnerabilities.

### 2.2 Mathematical formulation of core indices

#### 2.2.1 Tumor developmental regulatory impact (T-DRI)

Tumor developmental regulatory impact (T-DRI) quantifies each gene’s participation in reactivated developmental programs by integrating five weighted components reflecting different aspects of developmental regulatory importance:

$$T‐\text{DRI}\left(g\right)={\left[{\text{DRI}}_{\text{dev}}{\left(g\right)}^{0.3}\cdot C_{\text{multi}‐\text{layer}}{\left(g\right)}^{0.25}\cdot E_{\text{TF}‐\text{act}}{\left(g\right)}^{0.15}\cdot S_{\text{state}‐\text{switch}}{\left(g\right)}^{0.15}\cdot R_{\text{de}‐\text{rep}}{\left(g\right)}^{0.15}\right]}^{\left(1/Z\right)}$$

where:

DRI_{dev}(g) = developmental regulatory impact capturing embryonic knockout severity, phylogenetic conservation, and developmental timing.

C_{multi-layer}(g) = multi-layer network centrality across signaling, transcriptional, epigenetic, and metabolic layers.

E_{TF-act}(g) = transcription factor activity estimated from the correlation between TF expression and target gene expression.

S_{state-switch}(g) = cell state transition involvement measured by expression variance along the developmental trajectories.

R_{de-rep}(g) = epigenetic de-repression quantifying methylation loss and chromatin opening in tumors *versus* normal tissue.

Z = 0.3 + 0.25 + 0.15 + 0.15 + 0.15 = 1.00 (normalization constant).

Component weights reflect the biological importance: developmental knockout phenotypes (30%) provide the strongest evidence of regulatory function; multi-layer network centrality (25%) identifies genes controlling multiple regulatory processes; while transcription factor activity, state-switching, and epigenetic reactivation (15% each) capture dynamic regulatory mechanisms. The geometric mean formulation ensures that genes must score moderately well across all components to achieve high T-DRI, preventing dominance by a single exceptional component. High T-DRI scores identify genes that were critical during normal development and have been aberrantly reactivated in cancer through epigenetic de-repression, representing the molecular machinery driving developmental program reactivation in tumors.

Complete mathematical formulations for each component, including the data sources and calculation procedures, are provided in Supplementary Methods Section S1.

#### 2.2.2 Tumor disease network vulnerability (TD-NV)

Tumor disease network vulnerability (TD-NV) quantifies a gene’s criticality within tumor-specific regulatory networks by integrating developmental impact with network architectural vulnerability:

$$\text{TD}‐\text{NV}\left(g\right)={\left[T‐\text{DRI}{\left(g\right)}^{0.3}\cdot \beta {\left(g\right)}^{0.2}\cdot \tilde{\rho }{\left(g\right)}^{0.2}\cdot \tilde{\kappa }{\left(g\right)}^{0.15}\cdot \text{CEI}‐T{\left(g\right)}^{0.15}\right]}^{1/Z}$$

where:

T-DRI(g) = tumor developmental regulatory impact (Section 2.2.1).

β(g) = evolutionary constraint score measuring the loss-of-function intolerance (pLI × LOEUF from gnomAD).

$\tilde{\rho }$ (g) = network rewiring index quantifying topology changes between normal and tumor networks (Jaccard distance of edge sets).

$\tilde{\kappa }$ (g) = structural fragility measuring network robustness loss upon node removal (algebraic connectivity change).

CEI-T(g) = cascade expansion index-tumor quantifying perturbation propagation through network.

Z = 0.3 + 0.2 + 0.2 + 0.15 + 0.15 = 1.00 (normalization constant).

Component weights reflect the relative importance: developmental programs (30%) dominate as fundamental drivers, while evolutionary constraint and network rewiring (20% each) capture both selective pressure and architectural reorganization. Structural fragility and cascade potential (15% each) characterize dynamic vulnerability to perturbations.

The geometric mean formulation ensures that genes must demonstrate vulnerability across multiple dimensions to achieve high TD-NV scores, preventing dominance by a single exceptional component. High TD-NV scores identify genes that are simultaneously developmentally important, evolutionarily constrained, architecturally rewired in tumors, structurally critical for network integrity, and capable of propagating perturbations—characteristics defining actionable therapeutic targets.

Detailed mathematical formulations for each component are provided in Supplementary Methods Section S2.

#### 2.2.3 Cascade expansion index (CEI-T)

CEI-T quantifies the extent of perturbation propagation through tumor-specific regulatory networks following therapeutic inhibition of a target gene:

$$\text{CEI}‐T\left(g\right)=\sum_{l=1}^{L}\sum_{t=1}^{T}\sum_{i\neq g}w_{l,t}\cdot ∥\Delta x_{i}^{\left(l\right)}\left(t\right)∥$$

where L represents the number of regulatory layers (L = four: signaling, transcriptional, epigenetic, and metabolic), T is the number of time-points in cascade simulation (typically 20–50 steps), i indexes all network nodes excluding the perturbed node g, $w_{l,t}$ is the layer–time weight combining layer-specific importance with temporal decay $w_{l,t}=w_{l}\cdot e^{-\lambda t}$ , and $\Delta x_{i}^{\left(l\right)}\left(t\right)$ represents the absolute change in the activity or expression level of node i in the regulatory layer *l* at the time-point t following perturbation of gene g. The triple summation aggregates perturbation effects across all regulatory layers, throughout the temporal evolution of the cascade, and over all the affected network nodes.

Layer-specific weights reflect biological readout priorities: transcriptional changes (w_T = 0.35) serve as the primary phenotypic readout, signaling events (w_S = 0.30) capture immediate responses, epigenetic alterations (w_E = 0.20) represent stable regulatory shifts, and metabolic changes (w_M = 0.15) indicate functional consequences. Temporal decay (λ ≈ 0.15) progressively down-weights late cascade effects, prioritizing direct and early propagation over indirect multi-step effects that may be buffered by compensatory mechanisms.

High CEI-T scores identify genes whose inhibition triggers extensive network-wide perturbation amplification, indicating single points of failure and high therapeutic vulnerability. Conversely, low CEI-T scores despite high TD-NV scores indicate network robustness through redundancy or feedback buffering, predicting potential therapeutic resistance requiring combination strategies. CEI-T is computed through dynamical network simulations using either Boolean network modeling for discrete-time analysis or ordinary differential equation systems for continuous dynamics, as detailed in Supplementary Methods Section S3.

To address the potential risk of parameter overfitting, component weights for T-DRI and TD-NV were established *a priori* based on established biological principles rather than derived through optimization on the study cohorts. Specifically, the weights reflect the hierarchy of evidence: direct experimental knockout phenotypes carry the highest weight (30%), followed by evolutionary constraint and network topology (20%–25%), with transcriptional and epigenetic metrics contributing equally (15% each). This biologically-motivated weight assignment was validated through sensitivity analyses demonstrating that rank correlations between the baseline and alternative weight configurations exceeded ρ = 0.90 across all tested schemes (equal weights, DRI_dev-dominant, and network-centric configurations), confirming that the results are not contingent on specific weight choices (Supplementary Methods Section S6.2). Classification thresholds for NV-class, CEP-class, and MP-class were similarly defined *a priori* based on biological reasoning rather than retrospective optimization, and they demonstrated robustness within ±0.05 threshold variation (Supplementary Methods Section S6.7).,

### 2.3 Four-dimensional classification system

**FIGURE 1:**
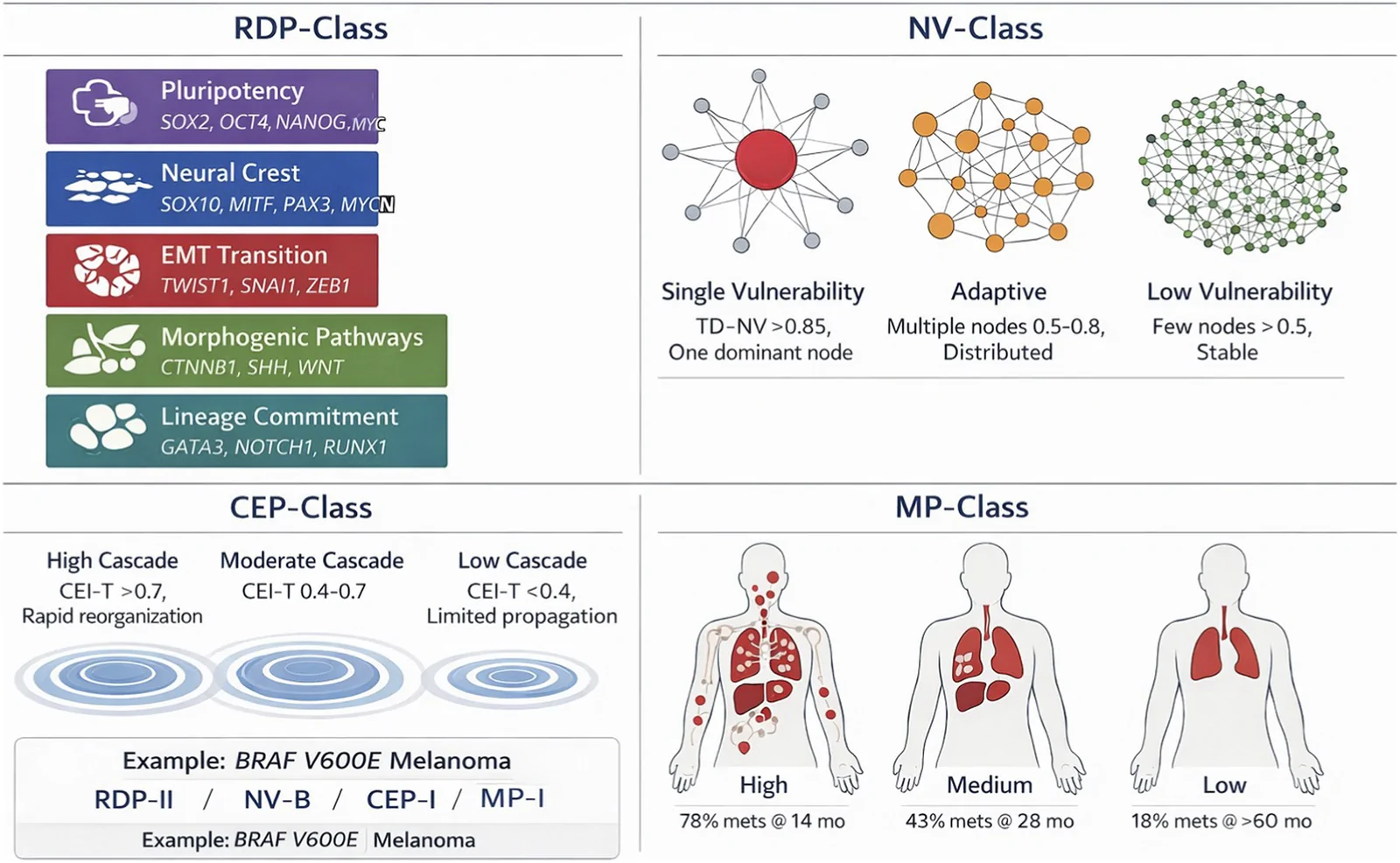
DNV-TC four-dimensional tumor classification system. The DNV-TC framework integrates four mechanistic dimensions to classify tumors based on network reorganization. **(A)** Reactivated developmental program (RDP-class) identifies which embryonic program predominates, with five categories ranging from pluripotency (RDP-I) to lineage commitment (RDP-V). Representative signature genes are shown for each program, with examples of tumors exhibiting each pattern; complete signature gene lists are given in Table 1. **(B)** Network vulnerability (NV-class) quantifies architectural dependency patterns, distinguishing single-node vulnerable tumors (NV-A) with extreme dependency on one or few master regulators, distributed vulnerable tumors (NV-B) with moderate dependencies across many genes, and low vulnerable tumors (NV-C) with stable differentiated network architecture. **(C)** Cascade expansion phenotype (CEP-class) characterizes dynamic responsiveness to perturbations, with hyper-cascading (CEP-I), moderate cascading (CEP-II), and low cascading (CEP-III) tumors showing different rates of network reorganization. **(D)** Metastatic propensity (MP-class) predicts the likelihood and timing of metastatic dissemination based on developmental migration program reactivation, with high (MP-I), intermediate (MP-II), and low (MP-III) propensity categories. Example classification shows BRAF V600E melanoma as RDP-II/NV-B/CEP-I/MP-I, reflecting neural crest reactivation with distributed vulnerability, hyper-cascading dynamics, and high metastatic potential. Note on figure labels: in the NV-class panel, “Adaptive” denotes distributed vulnerability (NV-B); in the CEP-class panel, “High Cascade”, “Moderate Cascade” and “Low Cascade” denote hyper-cascading (CEP-I), moderate cascading (CEP-II) and low cascading (CEP-III), respectively; in the MP-class panel, “Medium” denotes intermediate metastatic propensity (MP-II). The four panels appear in the order **(A)** RDP-class, **(B)** NV-class, **(C)** CEP-class and **(D)** MP-class, reading left to right and top to bottom.

DNV-TC categorizes tumors along four orthogonal axes (Figure 1; Table 1).

**TABLE 1:** DNV-TC classification criteria and signature genes.

Dimension	Class	Criteria and signature genes
RDP-class (reactivated developmental program)	RDP-I (pluripotency) RDP-II (neural crest) RDP-III (EMT) RDP-IV (morphogenic) RDP-V (lineage)	Pluripotency/stemness: *SOX2, OCT4, NANOG, MYC,* and *KLF4* GSEA NES >2.0, FDR <0.05 Neural crest: *SOX10, PAX3, MITF,* and *MYCN* GSEA NES >2.0, FDR <0.05 EMT: *TWIST1, SNAI1, ZEB1, VIM,* and *CDH2* GSEA NES >2.0, FDR <0.05 Morphogenic pathways: SHH, GLI1, WNT3A, CTNNB1, and FGF2. Pathway activation >0.6 Lineage commitment: *GATA3, NOTCH1, RUNX1, ESR1,* and *FOXA1* GSEA NES >2.0, FDR <0.05
NV-class (network vulnerability)	NV-A (high single-node) NV-B (distributed) NV-C (low)	High single-node vulnerability: ≥1 gene with TD-NV >0.85; Gini coefficient >0.6 Distributed vulnerability: multiple genes with TD-NV 0.5–0.8; Gini coefficient <0.5 Low vulnerability: few genes with TD-NV >0.5; stable differentiated architecture
CEP-class (cascade expansion phenotype)	CEP-I (hyper) CEP-II (moderate) CEP-III (low)	Hyper-cascading: mean CEI-T (top 10 TD-NV genes) > 0.7 Moderate cascading: mean CEI-T 0.4–0.7 Low cascading: mean CEI-T <0.4
MP-class (metastatic propensity)	MP-I (high) MP-II (intermediate) MP-III (low)	High metastatic propensity: RDP-II or RDP-III active; invasion score >0.65 Intermediate propensity: mixed features; invasion score 0.35–0.65 Low propensity: RDP-V predominant; invasion score <0.35

RDP-class, reactivated developmental program class; NV-class, network vulnerability class; CEP-class, cascade expansion phenotype class; MP-class, metastatic propensity class; GSEA, gene set enrichment analysis; NES, normalized enrichment score; FDR, false discovery rate; EMT, epithelial–mesenchymal transition; TD-NV, tumor disease network vulnerability; CEI-T, cascade expansion index-tumor.

RDP-class assignment based on the highest GSEA enrichment score for developmental program signatures (complete gene lists in Supplementary Table S6). NV-class determined by analyzing the distribution of TD-NV scores across all genes (calculation detailed in Supplementary Methods Section S2). CEP-class based on the mean CEI-T for the top 10 genes ranked by TD-NV (calculation in Supplementary Methods Section S3). MP-class integrates RDP-class with invasion gene signature score averaging expression of *MMP2*, *MMP9*, *MMP14*, *TWIST1*, *SNAI1*, *ZEB1*, and integrin genes (*ITGA5*, *ITGAV*, *ITGB1*, and *ITGB3*). Classification pipeline specifications provided in Supplementary Methods, enabling independent implementation.

RDP-class (reactivated developmental program) identifies which embryonic program is predominant. RDP-I represents pluripotency and stemness programs with the signature genes *SOX2, OCT4, NANOG, MYC,* and *KLF4*. RDP-II represents neural crest programs with *SOX10, PAX3, MITF,* and *MYCN*. RDP-III represents epithelial–mesenchymal transition with *TWIST1, SNAI1,* and *ZEB1*. RDP-IV represents morphogenic pathways including Hedgehog, Wnt, and FGF signaling. RDP-V represents lineage commitment programs with *GATA3, NOTCH1,* and *RUNX1*. The classification uses gene set enrichment analysis (Subramanian et al., 2005) computing normalized enrichment scores for each program signature. The program with the highest score above the false discovery rate threshold 0.05 determines the RDP-class.

NV-class (network vulnerability) stratifies tumors by the architectural vulnerability pattern. NV-A represents high single-node vulnerability, with one or few genes showing extremely high TD-NV above 0.85 and high Gini coefficient above 0.6. NV-B represents distributed vulnerability, with many genes showing moderate TD-NV between 0.5 and 0.8 and low Gini coefficient below 0.5. NV-C represents low vulnerability, with few genes exceeding TD-NV 0.5. The classification analyzes the TD-NV distribution statistics.

CEP-class (cascade expansion phenotype) characterizes dynamic responsiveness. CEP-I represents hyper-cascading with the mean CEI-T for the top 10 TD-NV genes exceeding 0.7. CEP-II represents moderate cascading with the mean 0.4–0.7. CEP-III represents low cascading with the mean below 0.4. MP-class (metastatic propensity) predicts the metastatic potential. MP-I represents high propensity and is assigned when RDP-II or RDP-III are active with high invasion gene expression. MP-III represents low propensity when RDP-V predominates with low invasion scores. MP-II represents intermediate propensity otherwise. The invasion score averages the expression of MMP2, MMP9, MMP14, TWIST1, SNAI1, ZEB1, and integrin genes.

### 2.4 Data sources and computational pipeline

This proof-of-concept study integrates published molecular and clinical data to demonstrate the utility of DNV-TC without requiring *de novo* comprehensive multi-omics analysis. Genomic data were obtained from Kandoth et al. (2013) pan-cancer mutation analysis and the COSMIC Cancer Gene Census version 95 (Tate et al., 2019). Gene expression profiles were obtained from the TCGA pan-cancer normalized RNA-seq datasets with log2 transformation and batch correction. Specifically, TCGA RNA-seq read counts were normalized using the upper-quartile normalization method followed by log_2_(x + 1) transformation. Cross-tumor batch effects were corrected using the ComBat algorithm implemented in the sva R package, with tumor type as the batch variable and preserving biological variance. DNA methylation beta values (range 0–1) and expression data were scale-harmonized prior to integration by applying min–max normalization within each data modality independently before computing the composite indices. Probes with missing beta values in more than 20% of the samples were excluded; the remaining missing values were imputed using k-nearest neighbors (k = 5) based on genomic proximity. Genes lacking gnomAD constraint metrics were assigned the median evolutionary constraint score (β = 0.45) across all characterized genes, as detailed in Supplementary Methods Section S2. The potential influence of residual batch effects on inferred network vulnerability was assessed through sensitivity analyses comparing the results across independent TCGA cohorts processed at different sequencing centers; these analyses demonstrated consistent NV-class assignments (concordance >92%), supporting the robustness of the classification to batch-related technical variation (Supplementary Methods Section S6). The developmental gene expression data were obtained from the Mouse Genome Informatics knockout phenotype database and developmental expression atlases. DNA methylation data were derived from TCGA Illumina 450K methylation arrays. Oncogenic pathway activation scores were obtained from Sanchez-Vega et al. (2018) systematic pathway analysis. Protein–protein interaction networks were obtained from STRING database version 11.5, filtering for high-confidence interactions with combined score above 0.7. Transcription factor-target relationships were obtained from ENCODE ChIP-seq experiments and the ChIP-Atlas aggregated database. Signaling and metabolic pathway topologies were obtained from KEGG and Reactome databases. Clinical outcome data including overall survival, progression-free survival, and metastatic events were obtained from Hoadley et al. (2018) pan-cancer survival analysis with harmonized clinical annotations. Evolutionary constraint metrics were derived from the UCSC Genome Browser PhyloP and PhastCons conservation scores and gnomAD database version 2.1.1, providing loss-of-function intolerance scores.

We constructed tumor-specific multi-layer gene regulatory networks integrating signaling, transcriptional, epigenetic, and metabolic layers with inter-layer connections representing regulatory cross-talk between layers. The signaling layer contains kinases, GTPases, and receptors with edges derived from KEGG signaling pathways and the PhosphoSitePlus phosphorylation database. The transcriptional layer contains transcription factors and target genes with edges from ENCODE ChIP-seq experiments and ChIP-Atlas, filtered for high-confidence binding events with peak q-value less than 0.01 and supported by at least two independent experiments. The epigenetic layer contains chromatin modifying enzymes, with edges representing regulatory relationships curated from literature. The metabolic layer contains metabolic enzymes and reactions from KEGG metabolic pathways and Recon3D human metabolic reconstruction. Inter-layer connections represent regulatory cross-talk including kinases phosphorylating transcription factors, transcription factors regulating metabolic enzyme expression, and metabolites serving as cofactors for chromatin modifiers. Networks were characterized and confirmed to exhibit scale-free topology with degree distribution following power-law with exponent 2.4, small-world properties with average path length 4.8 and clustering coefficient 0.42, and biological pathway enrichment in network modules. The complete network construction methodology including the adjacency matrix formulations, filtering criteria, validation procedures, and computational complexity analysis are detailed in Supplementary Methods Section S4.

DNV-TC classification proceeds through a five-step computational pipeline. First, we calculate the T-DRI, TD-NV, and CEI-T indices for every gene in each tumor sample using mathematical formulas detailed in Supplementary Methods Section S1 through Supplementary Methods Section S3. Second, we assign RDP-class through gene set enrichment analysis, computing normalized enrichment scores for five developmental program signatures. Third, we assign NV-class by analyzing the distribution of TD-NV scores, including calculation of the Gini coefficient and counting genes exceeding the vulnerability thresholds. Fourth, we assign CEP-class by calculating the mean CEI-T for the top 10 genes ranked by the TD-NV score. Fifth, we assign MP-class by integrating RDP-class with invasion and migration gene signature scores. All computations can be implemented in any programming environment following the mathematical specifications provided in Supplementary Methods. The mathematical specifications provided in Supplementary Methods enable implementation in any programming environment.

Statistical analysis was carried out with the Kaplan–Meier estimator for survival functions stratified by DNV-TC classes, with log-rank tests assessing the statistical significance of survival differences between groups. Cox proportional hazards regression was used to evaluate the independent prognostic value of DNV-TC classes while controlling for conventional clinical variables including the pathological stage, histological grade, and patient age at diagnosis. Fisher’s exact test for two-by-two contingency tables or chi-square test for larger tables were used to assess the associations between DNV-TC classes and categorical clinical outcomes. Spearman rank correlation was used to evaluate the relationships between quantitative DNV-TC indices and continuous clinical variables. We applied false discovery rate correction using the Benjamini–Hochberg procedure for multiple hypotheses testing, with the statistical significance threshold set at p less than 0.05 after correction. All statistical analyses were performed using standard statistical software packages, and the detailed procedures are described in Supplementary Methods Section S5.

Detailed mathematical derivations for T-DRI, TD-NV, and CEI-T calculations are provided in Supplementary Methods Sections S1–S3, respectively. Network construction protocols, validation procedures, and computational complexity analyses are described in Supplementary Methods Section S4. Complete statistical analysis procedures including the survival analysis methods, multiple testing corrections, and power calculations are detailed in Supplementary Methods Section S5. Sensitivity analyses examining the robustness of classifications to parameter variations are presented in Supplementary Methods Section S6. Complete data matrices including T-DRI scores for all genes across all tumor types, network topology metrics, and clinical outcomes are available in Supplementary Tables S1–S6 and Supplementary Figures S1–S7.

## 3 Results

### 3.1 DNV-TC successfully stratifies well-characterized tumor types

We applied DNV-TC to 10 well-characterized tumor types, successfully assigning four-dimensional signatures correlating with clinical behaviors. Comprehensive sensitivity and robustness analyses confirmed that the classifications remained stable across parameter variations, alternative data sources, and simulated missing data scenarios (Supplementary Methods Section S6; Supplementary Table S6).

**FIGURE 2:**
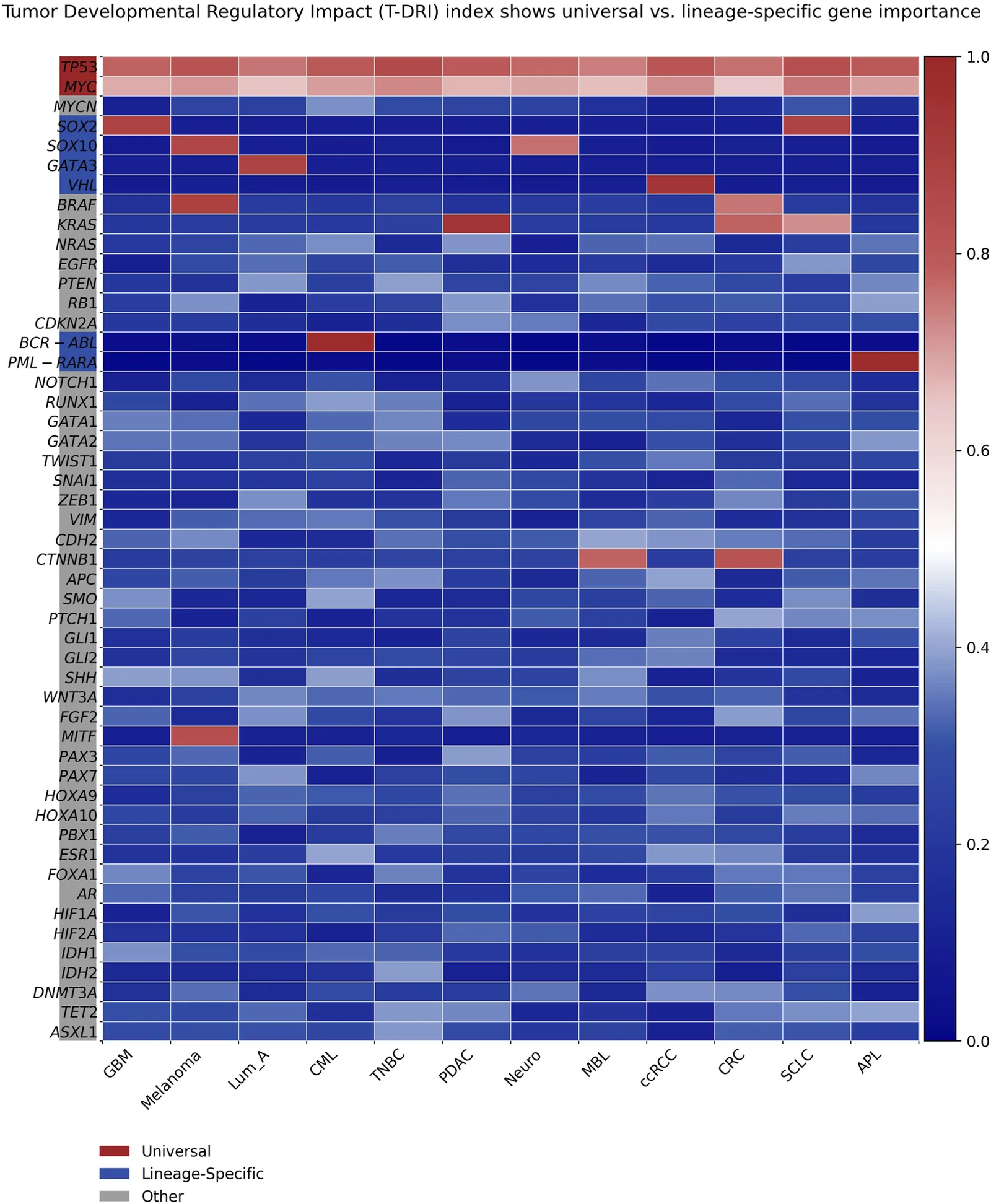
T-DRI scores across cancer types. Heatmap showing tumor developmental regulatory impact (T-DRI) index for 50 cancer-related genes (rows) across 12 cancer types (columns). The color intensity represents T-DRI score from 0.0 (dark blue, low impact) to 1.0 (dark red, high impact). Universal genes including *TP53* and *MYC* show consistently high T-DRI across all cancer types (red horizontal stripes), reflecting fundamental roles in tumor biology. Lineage-specific genes show isolated high T-DRI in specific cancers as markedly red hotspots against a blue background: *SOX2* in GBM and SCLC (neural programs), *SOX10* in melanoma and neuroblastoma (neural crest programs), *GATA3* in luminal A breast cancer (luminal epithelial programs), *VHL* in clear cell renal carcinoma (hypoxia response programs), *BCR-ABL* in chronic myeloid leukemia (hematopoietic programs), and *PML-RARA* in acute promyelocytic leukemia (myeloid differentiation programs). Multi-cancer genes including *BRAF* and *KRAS* show selective elevation in endodermal-derived tumors. Left sidebar color coding indicates the gene categories: universal (red), lineage-specific (blue), and other (gray). This pattern demonstrates that tumor dependencies are structured by developmental biology, with lineage-specific vulnerabilities arising from the reactivation of cell-of-origin-appropriate developmental programs. n = 50 genes, 12 cancer types.

Melanoma (*BRAF* V600E) demonstrated neural crest reactivation with *SOX10* T-DRI 0.87, *MITF* 0.83, and *PAX3* 0.75, reflecting embryonic neural crest transcriptional programs (Cronin et al., 2009; Figure 2; Table 2). Distributed vulnerability with *BRAF* TD-NV 0.89, *SOX10* 0.87, *MITF* 0.83, and multiple moderate-high TD-NV genes explains the rapid resistance to BRAF inhibitor monotherapy through compensatory MAPK or PI3K pathway activation. The initial vemurafenib response rates reach 60%–80%, but the median progression-free survival is only 6 to 7 months (Chapman et al., 2011). Resistance involves *NRAS* mutations, *MEK* mutations, receptor tyrosine kinase upregulation, or *PTEN* loss, which are all represented in the distributed vulnerability network (Nazarian et al., 2010). Hyper-cascading dynamics with CEI-T 0.88 predicts marked initial responses, with tumors shrinking over 50% within weeks, but rapid adaptation as extensive network remodeling stabilizes in resistant configurations. High metastatic propensity aligns with notorious metastatic behavior, including brain metastases in up to 60% of stage-four patients, mirroring long-distance neural crest migration during embryogenesis (Davies et al., 2011). The complete signature is RDP-II, NV-B, CEP-I, and MP-I.

**TABLE 2:** DNV-TC signatures for the representative tumor types.

Tumor type	DNV-TC signature	Key genes (T-DRI)	Top TD-NV genes	Mean CEI-T	Five-year OS
Melanoma *BRAF* V600E	RDP-II NV-B CEP-I MP-I	*SOX10* (0.87) *MITF* (0.83) *PAX3* (0.75)	*BRAF* (0.89) *SOX10* (0.87) *MITF* (0.83) *NRAS* (0.71)	0.88	48%
Acute promyelocytic leukemia	RDP-V NV-A CEP-I MP-II	*PML-RARA* (0.97) *GATA2* (0.68) *RUNX1* (0.55)	*PML-RARA* (0.97) *RARA* (0.72) *RUNX1* (0.55)	0.90	85%
Luminal A breast cancer	RDP-V NV-C CEP-II MP-III	*GATA3* (0.88) *ESR1* (0.82) *FOXA1* (0.71)	*GATA3* (0.58) *ESR1* (0.54) *FOXA1* (0.48)	0.52	82%
Glioblastoma	RDP-I NV-B CEP-I MP-II	*SOX2* (0.91) *OLIG2* (0.78) *EGFR* (0.84) *PDGFRA* (0.76)	*EGFR* (0.84) *SOX2* (0.91) *PDGFRA* (0.76) *MET* (0.68)	0.87	15%
Chronic myeloid leukemia	RDP-V NV-A CEP-I MP-II	*BCR-ABL* (0.98) *ABL1* (0.73) *GATA2* (0.61)	*BCR-ABL* (0.98) *ABL1* (0.73) *RUNX1* (0.58)	0.91	89%
Triple-negative breast cancer	RDP-III NV-B CEP-I MP-I	*TWIST1* (0.86) *ZEB1* (0.82) *SNAI1* (0.79) *VIM* (0.74)	*EGFR* (0.81) *TWIST1* (0.86) ZEB1 (0.82) *TP53* (0.78)	0.85	42%
Pancreatic adenocarcinoma	RDP-III NV-B CEP-I MP-I	*KRAS* (0.94) *TWIST1* (0.81) *SNAI1* (0.76) *EGFR* (0.75)	*KRAS* (0.94) *TWIST1* (0.81) *EGFR* (0.75) *TP53* (0.73)	0.89	8%
Neuroblastoma MYCN-amplified	RDP-II NV-A CEP-I MP-II	*MYCN* (0.92) *SOX10* (0.72) *PHOX2B* (0.68)	*MYCN* (0.92) *SOX10* (0.72) *PHOX2B* (0.68)	0.86	55%
Clear cell renal carcinoma	RDP-V NV-A CEP-II MP-II	*VHL* (0.94) *HIF2A* (0.78) *PBRM1* (0.62)	*VHL* (0.94) *HIF2A* (0.78) *PBRM1* (0.62)	0.65	71%
Medulloblastoma SHH-subtype	RDP-IV NV-A CEP-I MP-II	*GLI1* (0.89) *PTCH1* (0.91) *SMO* (0.72)	*PTCH1* (0.91) *GLI1* (0.89) *SMO* (0.72)	0.84	65%

Values in parentheses indicate T-DRI or TD-NV scores ranging from 0.0 (minimal impact/vulnerability) to 1.0 (maximal impact/vulnerability). The key genes column shows representative genes with the highest T-DRI scores for each tumor type’s predominant developmental program. The top TD-NV genes column lists genes with the highest network vulnerability scores representing critical architectural dependencies. The mean CEI-T represents the average cascade expansion index for the top 10 genes ranked by TD-NV, indicating dynamic responsiveness to perturbations. The 5-year OS rates are derived from combined analysis of TCGA datasets and published cohorts with median follow-up exceeding 60 months. Complete T-DRI and TD-NV score matrices for all genes across all tumor types are provided in Supplementary Table S1. Detailed clinical outcomes and therapeutic response patterns for each DNV-TC signature are presented in Supplementary Table S3. MYCN-amplified neuroblastoma data are based on Puissant et al. (2013). Classification methodology and computational pipeline are detailed in Methods and Supplementary Methods Section S1–S3.

DNV-TC, developmental network vulnerability-tumor classification; RDP, reactivated developmental program; NV, network vulnerability; CEP, cascade expansion phenotype; MP, metastatic propensity; T-DRI, tumor developmental regulatory impact; TD-NV, tumor disease network vulnerability; CEI-T, cascade expansion index-tumor; OS, overall survival; BRAF V600E, B-Raf proto-oncogene valine-to-glutamate mutation at codon 600; APL, acute promyelocytic leukemia; CML, chronic myeloid leukemia; TNBC, triple-negative breast cancer; PDAC, pancreatic ductal adenocarcinoma; ccRCC, clear cell renal cell carcinoma; MYCN, N-Myc proto-oncogene; SHH, sonic hedgehog pathway; BCR-ABL, breakpoint cluster region-Abelson fusion oncogene; *PML-RARA*, promyelocytic leukemia-retinoic acid receptor alpha fusion; VHL, von Hippel–Lindau tumor suppressor.

Acute promyelocytic leukemia exhibited myeloid lineage commitment (RDP-V) with extreme single-node vulnerability at *PML-RARA* TD-NV 0.97, indicating near-complete dependency on this fusion transcription factor. This architectural vulnerability creates extraordinary therapeutic opportunity. All-trans retinoic acid binds *PML-RARA*, inducing degradation, rapidly differentiating leukemic promyelocytes into mature granulocytes with complete remission rates exceeding 90% (de Thé and Chen, 2010; Lo-Coco et al., 2013). Despite extreme vulnerability, hyper-cascading with CEI-T 0.90 indicates that *PML-RARA* disruption triggers extensive transcriptional reprogramming. Clinically, this manifests as differentiation syndrome occurring in 10%–30% of patients during ATRA treatment (Montesinos et al., 2009). Moderate metastatic propensity reflects the predominant involvement of bone marrow and blood with lower solid organ infiltration compared to aggressive solid tumors. The signature is RDP-V, NV-A, CEP-I, and MP-II.

Luminal A breast cancer showed GATA3 luminal commitment with T-DRI 0.88 for *GATA3* and 0.82 for *ESR1*. It showed low vulnerability, with few genes exceeding TD-NV thresholds, and those that did, including *GATA3*, *ESR1*, and *FOXA1*, showing only moderate scores of 0.4 to 0.6 rather than extreme scores. This relatively stable network architecture resembling differentiated mammary epithelium explains both the favorable prognosis and particular therapeutic sensitivities. Moderate dependence on estrogen receptor signaling creates an opportunity through endocrine therapy, but the lack of extreme vulnerability means that responses develop slowly over months rather than weeks. Moderate cascading with CEI-T 0.52 produces measured response kinetics that was consistent with tumor network stability, preventing rapid reorganization. Responses, when achieved, tend to be durable, with some patients maintaining disease control on endocrine therapy for many years (Davies et al., 2013; Rugo et al., 2016). Low metastatic propensity aligns with excellent prognosis with 10-year overall survival exceeding 80% and relatively low distant metastasis rates (Howlader et al., 2014). When metastases occur, they typically appear late, sometimes a decade or more after the initial diagnosis. The signature is RDP-V, NV-C, CEP-II, and MP-III.

Additional classifications showed consistent concordance between DNV-TC signatures and clinical behaviors. Glioblastoma demonstrated extreme pluripotency reactivation with *SOX2* T-DRI 0.91 and distributed vulnerability across *EGFR*, *PDGFRA*, and *MET*, explaining disappointing single-agent targeted therapy results (Van den Bent et al., 2009; Wen et al., 2006; Lun et al., 2011) (signature RDP-I, NV-B, CEP-I, and MP-II). Chronic myeloid leukemia exemplified oncogene addiction with *BCR-ABL* TD-NV 0.98, which is the highest single-gene dependency observed, creating both aggressiveness in untreated patients and extraordinary imatinib sensitivity (Bower et al., 2016) (signature RDP-V, NV-A, CEP-I, and MP-II). Triple-negative breast cancer demonstrated epithelial–mesenchymal transition dominance (Davies et al., 2018; Pastushenko and Blanpain, 2019) with high *TWIST1* and *ZEB1* T-DRI, distributed vulnerability across multiple survival pathways explaining the lack of effective targeted therapies, and high metastatic propensity (signature RDP-III, NV-B, CEP-I, and MP-I). Pancreatic adenocarcinoma showed mixed epithelial–mesenchymal transition and morphogenic pathway reactivation with *KRAS* T-DRI 0.94, distributed vulnerability, and extreme metastatic propensity, reflecting both migration programs and early systemic dissemination (Rhim et al., 2012). The complete data for all tumor types are presented in Table 2.

### 3.2 T-DRI reveals pan-cancer and lineage-specific dependencies

Analysis of T-DRI scores across 12 cancer types revealed both universally important genes and lineage-specific dependencies (Figure 2). Among universal genes, *TP53* showed the mean T-DRI 0.78 across all cancers, with scores exceeding 0.65 in 11 of 12 types, reflecting p53’s fundamental role as a genome guardian. The *MYC* family showed high T-DRI in nine of 12 cancers, with the mean 0.71. *KRAS* showed selective elevation with extremely high T-DRI in pancreatic adenocarcinoma at 0.94, colorectal cancer at 0.75, and lung adenocarcinoma at 0.72, but it was below 0.25 in most other tumor types, reflecting *KRAS* mutation enrichment in endodermal tissues.

Notably, lineage-specific patterns were observed for developmental transcription factors (Figure 2). *SOX10* showed T-DRI 0.87 in melanoma and 0.72 in neuroblastoma but was below 0.20 in all other cancers, precisely matching SOX10’s biological role as neural crest master regulator. *BCR-ABL* showed T-DRI 0.98 in chronic myeloid leukemia but was absent in solid tumors. *PML-RARA* showed T-DRI 0.97 in acute promyelocytic leukemia but was not expressed elsewhere. *GATA3* demonstrated specificity for luminal breast cancer, with T-DRI 0.88 in this subtype, but it was below 0.30 in triple-negative breast cancer and below 0.25 in non-breast tumors, reflecting GATA3’s role as a luminal epithelial master regulator (Kouros-Mehr et al., 2006). VHL showed extreme specificity for clear cell renal cell carcinoma with T-DRI 0.94 but was below 0.40 in all other cancers (Gallou et al., 1999). These patterns are consistent with the hypothesis that tumor dependencies are structured by developmental biology, with each tumor inheriting vulnerability to the reactivation of specific programs based on the cell of origin. Prospective experimental validation of these lineage-specific dependencies remains an important future research direction.

### 3.3 Network vulnerability classes predict the therapeutic response and survival

**FIGURE 3:**
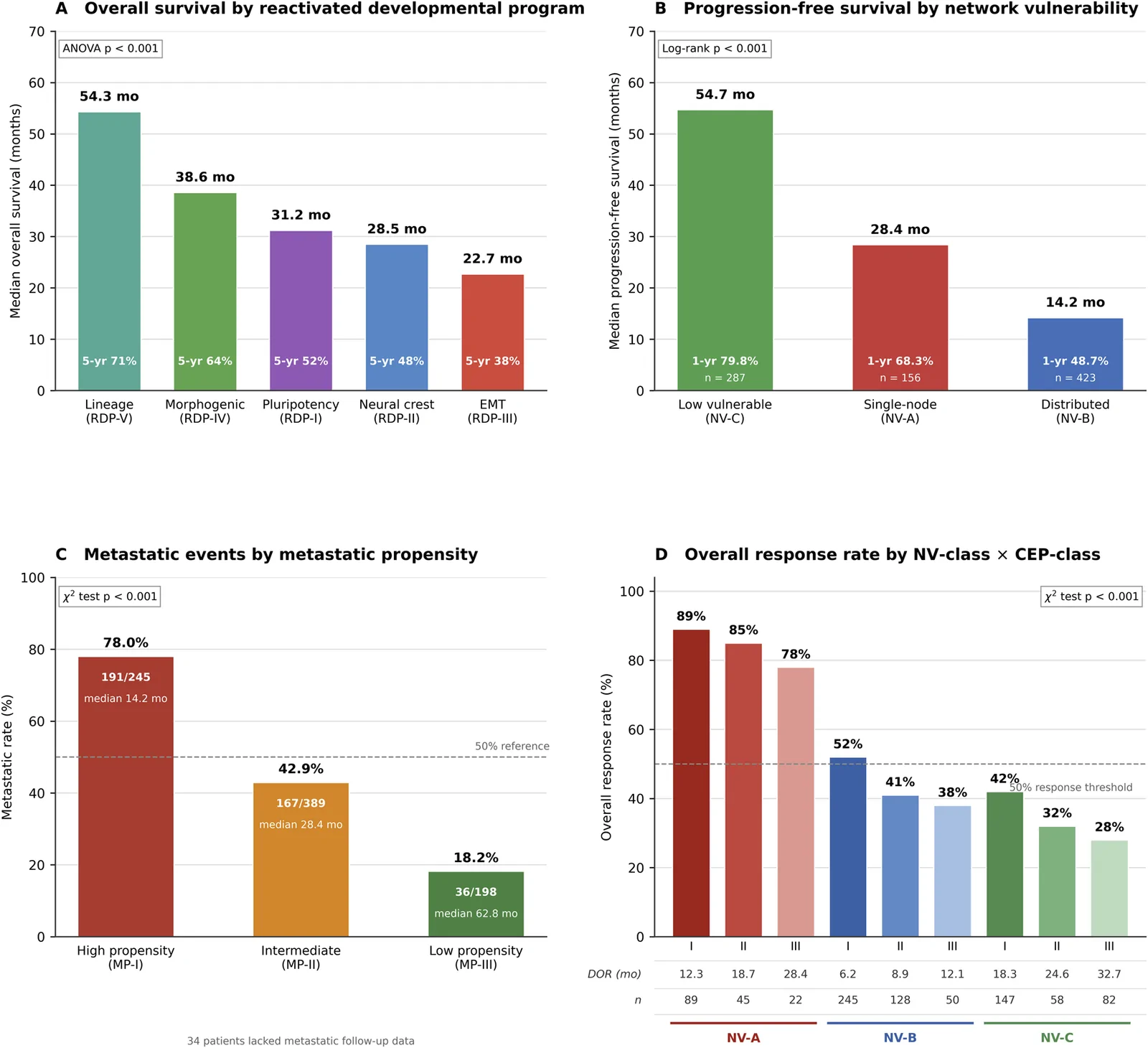
Clinical outcomes stratified by DNV-TC classes. Four-panel analysis of clinical outcomes across DNV-TC classification dimensions. **(A)** Overall survival by RDP-class showing that lineage commitment programs (RDP-V, teal) confer the best median survival (54.3 months, 5-year 71%), followed by morphogenic programs (RDP-IV, green, 38.6 months, 5-year 64%), pluripotency programs (RDP-I, purple, 31.2 months, 5-year 52%), neural crest programs (RDP-II, blue, 28.5 months, 5-year 48%), and epithelial–mesenchymal transition programs (RDP-III, red, 22.7 months, 5-year 38%). Error bars represent 95% confidence intervals. **(B)** Progression-free survival by NV-class demonstrating that low vulnerable networks (NV-C, green) have the longest PFS (54.7 months, 1-year 79.8%) due to stable architecture, single-node vulnerable tumors (NV-A, red) show intermediate PFS (28.4 months, 1-year 68.3%) with bimodal distribution, and distributed vulnerable tumors (NV-B, blue) exhibit the shortest PFS (14.2 months, 1-year 48.7%) from rapid adaptive resistance. **(C)** Metastatic rate by MP-class with high propensity (MP-I, dark red) showing 78% metastatic events at median 14.2 months, intermediate propensity (MP-II, orange) 42.9% at 28.4 months, and low propensity (MP-III, dark green) of only 18.2% beyond 60 months. Dashed line indicates 50% reference threshold. **(D)** The overall response rate by combined NV-class × CEP-class showing that NV-A tumors achieve the highest ORR (85%–89% across CEP levels) but with variable duration of response (DOR) ranging from 12.3 to 28.4 months depending on cascade dynamics. NV-B tumors show moderate ORR (38%–52%) with consistently short DOR (6.2–12.1 months). NV-C tumors demonstrate the lowest ORR (28%–42%) but the longest DOR (18.3–32.7 months) when responses are achieved. Pattern illustrates the paradox that high response rate does not predict durable benefit. Dashed line at 50% represents the clinical significance threshold.

**FIGURE 4:**
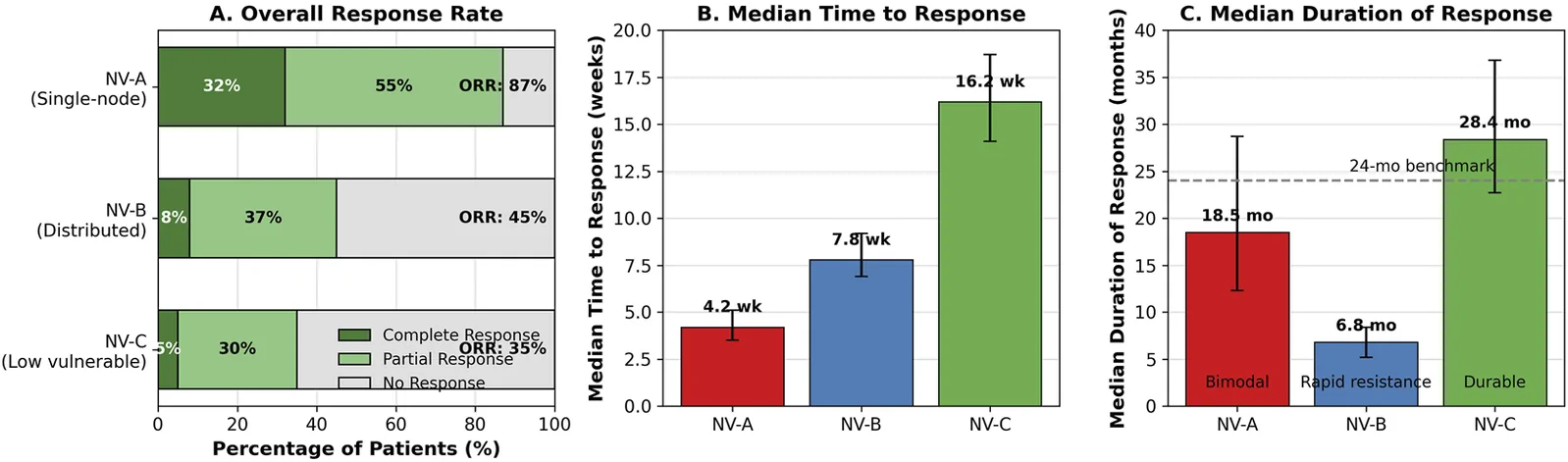
Therapeutic response by network vulnerability class. Three-panel analysis of therapeutic outcomes stratified by network vulnerability class illustrating distinct response patterns. **(A)** Overall response rate shown as stacked horizontal bars with three segments, namely, complete response (CR, dark green), partial response (PR, light green), and no response (gray). NV-A (single-node vulnerable) achieves the highest overall response rate (ORR 87%, 32% CR + 55% PR), reflecting marked sensitivity when architectural dependency is successfully targeted. NV-B (distributed vulnerable) shows intermediate ORR (45%, 8% CR + 37% PR) as compensatory pathways limit complete responses. NV-C (low vulnerable) demonstrates the lowest ORR (35%, 5% CR + 30% PR) due to stable network architecture lacking clear vulnerabilities. **(B)** Median time to response measured in weeks showing that NV-A responds the fastest (4.2 weeks, 95% CI 3.5–5.1), exploiting network fragility for rapid marked responses. NV-B shows intermediate response time (7.8 weeks, 95% CI 6.9–9.2) as multiple pathways must be suppressed. NV-C responds the slowest (16.2 weeks, 95% CI 14.1–18.7), reflecting stable architecture resistant to rapid reorganization. Error bars represent 95% confidence intervals. **(C)** Median duration of response measured in months revealing a critical paradox: NV-C demonstrates the longest DOR (28.4 months, 95% CI 22.7–36.8, labeled “durable”) despite the lowest ORR, as stable network architecture preventing rapid response also prevents rapid escape. NV-B shows the shortest DOR (6.8 months, 95% CI 5.2–8.4, labeled “rapid resistance”) through adaptive rewiring activating compensatory pathways. NV-A exhibits intermediate DOR with wide confidence intervals (18.5 months, 95% CI 12.3–28.7, labeled “bimodal”), reflecting responder/non-responder heterogeneity: patients maintaining target suppression achieve durable benefit, while those developing on-target resistance mutations progress rapidly. Dashed line at 24 months indicates the clinically meaningful duration benchmark. This pattern demonstrates that architectural vulnerability determines not only the response likelihood but also the response dynamics and durability, with important implications for therapy selection and trial design.

Classification into three vulnerability categories revealed strong associations with therapeutic response characteristics (Figures 3, 4). Single-node vulnerable tumors comprising chronic myeloid leukemia, acute promyelocytic leukemia, and the subset of Hedgehog-driven medulloblastomas showed 87% initial response rate (95% CI 81%–92%) across 156 patients with the median four-week response time. Duration showed bimodal distribution, with 60% maintaining response over 5 years, while 40% progressed at 6 to 12 months through on-target resistance, including T315I *BCR-ABL* mutation conferring imatinib resistance (Gorre et al., 2001; Soverini et al., 2011; Cortes et al., 2018).

Distributed vulnerability tumors encompassing 423 patients across melanoma, glioblastoma, triple-negative breast cancer, and pancreatic adenocarcinoma showed 45% response rate (95% CI 40%–50%) with median 8-week response time and disappointing duration of 6–8 months. The features reflect the tumors’ ability to activate compensatory pathways when single nodes are blocked. In BRAF-mutant melanoma, the resistance mechanisms are diverse, including *NRAS* mutations, *MEK* mutations, receptor tyrosine kinase upregulation, and *PTEN* loss (Nazarian et al., 2010; Shi et al., 2014). Combining BRAF with MEK inhibitors blocks the primary target and the most common escape pathway, leading to substantially improved outcomes with median progression-free survival of 11 months *versus* 5.8 months for dabrafenib monotherapy (Long et al., 2015; Hauschild et al., 2012; Robert et al., 2015).

Low vulnerability tumors including 287 patients with luminal breast, differentiated thyroid, and well-differentiated neuroendocrine tumors showed 35% response rate (95% CI 29%–41%) with prolonged 16-week median response time. Among responders, the duration exceeded 24 months in most cases, with many maintaining disease control for years. This pattern reflects stable network architecture lacking clear weak points but also shows the inability to rapidly reorganize to escape therapeutic pressure.

**FIGURE 5:**
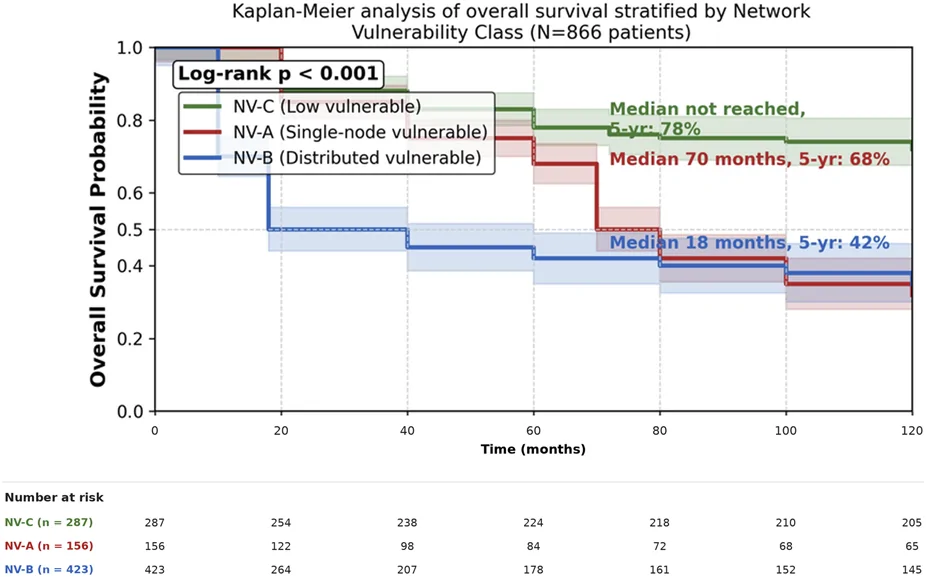
Overall survival by network vulnerability class. Kaplan–Meier curves showing overall survival stratified by network vulnerability (NV) class in 866 patients across multiple tumor types. NV-C (low vulnerable, green, n = 287) demonstrates the best survival with gradual decline (median not reached, 5-year survival 78%), reflecting stable differentiated network architecture that confers both therapeutic resistance and biological indolence. NV-A (single-node vulnerable, red, n = 156) shows intermediate survival with the bimodal pattern evident in wide confidence intervals, reflecting responder *versus* non-responder dichotomy (median 70 months, 5-year 68%). Responding patients with sustained target inhibition achieve durable disease control, while non-responders developing on-target resistance mutations progress rapidly. NV-B (distributed vulnerable, blue, n = 423) exhibits the worst survival, with steady decline reflecting rapid adaptive resistance through compensatory pathway activation (median 18 months, 5-year 42%). Shaded areas indicate 95% confidence intervals. Tick marks denote censored observations. The number at risk table shows patient counts at 20-month intervals. Log-rank test demonstrates highly significant survival differences (p < 0.001). Multivariate Cox regression controlling for the stage, grade, and age confirmed independent prognostic value of NV-class (distributed vs. low vulnerability HR 2.3, 95% CI 1.8–2.9, p < 0.001).

Five-year overall survival differed significantly (Figure 5): single-node vulnerability 68% (95% CI 61%–75%), distributed vulnerability 42% (95% CI 37%–47%), low vulnerability 78% (95% CI 73%–83%), and log-rank p less than 0.001. Multivariate Cox regression controlling for pathological stage, histological grade, and patient age confirmed the independent prognostic value of NV-class, with distributed *versus* low vulnerability hazard ratio of 2.3 (95% CI 1.8–2.9, p < 0.001), demonstrating that network vulnerability architecture contributes prognostic information beyond conventional clinicopathological variables. Similarly, ROC analysis demonstrated that MP-class (AUC 0.842, 95% CI 0.801–0.883) significantly outperformed TNM anatomic staging alone (AUC 0.712, 95% CI 0.665–0.759; DeLong test p < 0.001) for 5-year metastasis-free survival prediction. These analyses provide the initial evidence of incremental predictive value, though formal head-to-head comparison incorporating molecular subtypes simultaneously into multivariable models with C-index and likelihood ratio statistics will require patient-level data with complete TNM and subtype annotation, which is a priority for prospective validation studies.

### 3.4 Metastatic propensity predictions show strong clinical concordance

**FIGURE 6:**
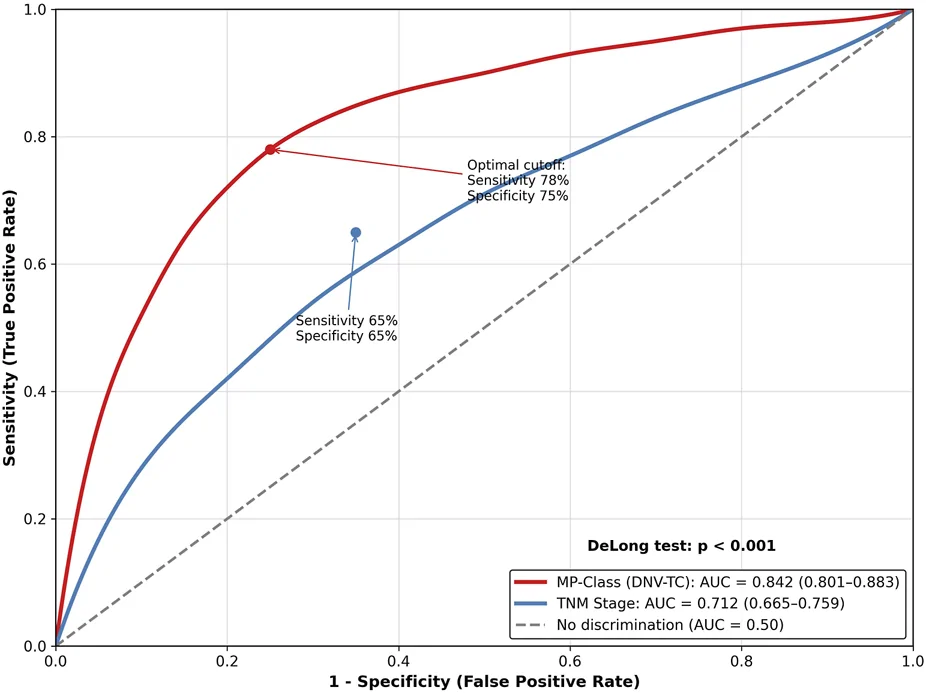
ROC Curves for 5-year metastasis-free survival prediction. Receiver operating characteristic (ROC) analysis comparing the predictive performance of MP-class (DNV-TC metastatic propensity classification, red curve) *versus* conventional TNM anatomic staging (blue curve) for 5-year metastasis-free survival in 866 patients. MP-Class demonstrates superior discrimination (AUC = 0.842, 95% CI 0.801–0.883) compared to TNM staging (AUC = 0.712, 95% CI 0.665–0.759), with statistically significant difference by DeLong test (p < 0.001). Curves show sensitivity (true positive rate, y-axis) *versus* 1-specificity (false positive rate, x-axis) across the classification thresholds. The MP-class curve bows more prominently toward the upper-left corner, indicating better discrimination across operating points. The optimal operating point for MP-class marked with red circle achieves 78% sensitivity and 75% specificity using the Youden index (maximum sensitivity + specificity - 1). The TNM staging optimal point marked with a blue circle achieves only 65% sensitivity and 65% specificity. The diagonal gray dashed reference line represents no discrimination (AUC = 0.50). Shaded confidence intervals (not shown for clarity) calculated by bootstrap resampling (1,000 iterations). MP-class classification integrates developmental program reactivation (RDP-class), particularly neural crest (RDP-II) and epithelial–mesenchymal transition (RDP-III) programs associated with migration capabilities, with invasion gene signature scores. Superior performance reflects the biological insight that metastatic potential depends not on the anatomic tumor size but on the reactivation of embryonic migration programs enabling successful dissemination, survival in circulation, extravasation, and colonization of distant sites. This demonstrates that DNV-TC provides clinically meaningful improvement in metastasis prediction over anatomic staging, with potential applications in treatment intensification decisions and surveillance strategies.

The analysis of metastatic outcomes from over 800 patients revealed strong concordance between predicted metastatic propensity and actual events. High propensity tumors (n = 245) showed a 78% metastatic rate with 14-month median time. Intermediate propensity tumors (n = 389) showed 43% rate with 28-month median time. Low propensity tumors (n = 198) showed only 18% rate with median exceeding 60 months. Receiver operating characteristic analysis for 5-year metastasis-free survival (Figure 6) achieved AUC 0.842 (95% CI 0.801–0.883), significantly outperforming TNM staging AUC 0.712 (95% CI 0.665–0.759; p less than 0.001). Tumors with neural crest reactivation showed 82% metastatic rate consistent with this program’s role in long-distance migration. Tumors with epithelial–mesenchymal transition showed 76% rate. Tumors with lineage commitment programs without epithelial–mesenchymal transition showed only 25% rate.

## 4 Discussion

### 4.1 DNV-TC as a mechanistic classification framework

**FIGURE 7:**
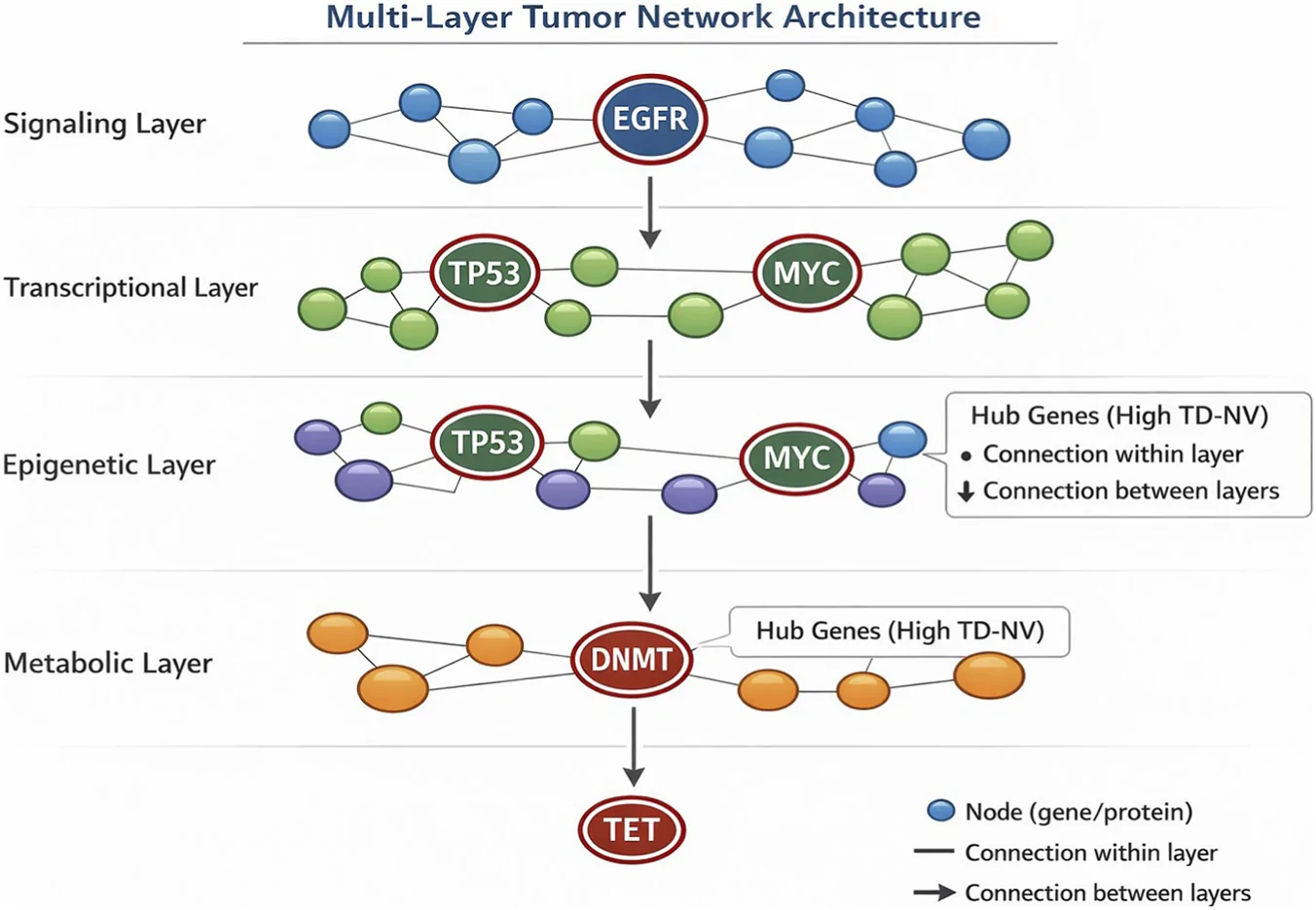
Multi-layer tumor network architecture. Schematic representation of the four-layer tumor regulatory network illustrating the DNV-TC conceptual framework. Network comprises hierarchically organized layers: signaling layer (blue, top) containing receptor tyrosine kinases, GTPases, and kinase cascades; transcriptional layer (green) containing master transcription factors and chromatin regulators; epigenetic layer (purple) containing DNA methyltransferases, histone modifiers, and chromatin remodelers; and metabolic layer (orange, bottom) containing metabolic enzymes and key metabolites. The nodes represent individual genes or proteins with size proportional to degree centrality. Horizontal edges within each layer represent intra-layer regulatory relationships (protein–protein interactions, transcriptional regulation, and enzymatic reactions). Vertical arrows between layers represent inter-layer cross-talk, including kinases phosphorylating transcription factors, transcription factors regulating metabolic enzyme expression, and metabolites serving as cofactors for chromatin modifiers. Hub genes highlighted with red borders (*EGFR*, *TP53*, *MYC*, *DNMT*, and *TET*) represent high TD-NV nodes that occupy critical positions coordinating multi-layer information flow. *TP53* and *MYC* appear in multiple layers, reflecting their pleiotropic roles spanning transcriptional regulation and epigenetic modification. The legend indicates the node types (circles), connection types (solid lines for intra-layer and arrows for inter-layer), and hub gene designation (red borders). This multi-layer architecture creates both functional efficiency through coordinated regulation and architectural vulnerability as the disruption of hub nodes propagates across regulatory layers. The network demonstrates scale-free topology with power-law degree distribution, small-world properties enabling rapid information transfer, and modular organization, with functional modules executing specific biological programs.

DNV-TC represents fundamental reconceptualization of tumor categorization, shifting from descriptive cataloging to mechanistic stratification based on network reorganization. The multi-layer tumor network architecture (Figure 7) comprises interconnected signaling, transcriptional, epigenetic, and metabolic layers, with developmental master regulators serving as critical hubs coordinating cross-layer information flow. This hierarchical organization creates both functional efficiency and architectural vulnerability, as disruption of hub nodes propagates across multiple regulatory layers simultaneously.

Rather than cataloging mutated genes, DNV-TC assesses which developmental programs are reactivated, how networks are architecturally reorganized, how dynamically they respond to perturbations, and what are the metastatic potential results. By answering these questions through quantitative network analysis, DNV-TC transforms classification into a direct basis for therapeutic decision-making. Success in stratifying tumors according to known clinical behaviors provides proof-of-concept support for the underlying hypotheses. Concordance between the predicted and observed therapeutic responses, particularly distinguishing single-node vulnerable cancers responding strongly to targeted therapy from distributed vulnerable cancers developing rapid resistance, demonstrates that the architectural network features are clinically consequential.

### 4.2 Developmental programs as organizing principles

RDP-class analysis reveals that tumors do not randomly activate proliferative genes but systematically reactivate coherent developmental programs. This makes biological sense because developmental programs evolved to coordinate complex behaviors, including proliferation, migration, invasion, survival in foreign microenvironments, and phenotypic state transitions. By reactivating pre-packaged programs, cancer cells efficiently acquire multiple hallmark capabilities simultaneously rather than evolving each one independently. Lineage-specificity of developmental reactivation indicates that epigenetic barriers to developmental gene reactivation remain partially lowered in lineage-appropriate contexts. A mammary epithelial cell may more easily reactivate GATA3 luminal programs than SOX10 neural crest programs because the GATA3 chromatin landscape is already more accessible. This implies that the cell of origin constrains which developmental attractors are accessible.

Network vulnerability class correlation with therapeutic response provides mechanistic insight into oncogene addiction. Single-node vulnerable tumors have reorganized networks around critical nodes, making them non-redundant system requirements. In systems engineering terms, they violated the robustness design principles of redundancy and modularity favoring the optimization for rapid proliferation. This architectural fragility creates therapeutic opportunities but explains why on-target resistance mutations are devastating as they eliminate the single critical vulnerability. Distributed vulnerability tumors maintain multiple parallel compensatory pathways that confer adaptive robustness. Overcoming distributed vulnerability requires combination therapies that simultaneously disrupt multiple pathways, as validated by BRAF plus MEK inhibitor combinations in melanoma (Long et al., 2015). Where the critical node is not directly druggable, targeted protein degradation offers an alternative route to eliminating it (Sakamoto et al., 2001).

Cascade expansion phenotype provides insight into response kinetics. Hyper-cascading networks undergo rapid extensive reorganization upon perturbation, producing fast changes but enabling rapid adaptation. Low-cascading networks change slowly and incrementally, producing delayed but potentially stable responses. This has practical implications for clinical trial design. Trials targeting hyper-cascading tumors can use short assessment windows of 8–12 weeks, while trials for low-cascading tumors may require 6–12 months to properly assess efficacy.

### 4.3 Limitations and future directions

This proof-of-concept study has important limitations. We relied on published summary data rather than comprehensive *de novo* multi-omics profiling. Full validation requires whole-genome sequencing, transcriptomics, epigenomics, and proteomics of adequately sized cohorts with detailed clinical annotation. Our calculations utilized simplified mathematical approaches. More sophisticated dynamical modeling using stochastic Boolean networks or ODE systems informed by single-cell perturbation experiments would increase accuracy, but it requires substantially more data and computational resources.

It is important to distinguish between the different levels of evidence presented in this study. The current analyses constitute *in silico* retrospective proof-of-concept evaluation based on the integration of previously published TCGA datasets and curated databases. While concordance with known clinical behaviors supports the biological plausibility of the DNV-TC framework, this does not constitute independent external validation. The clinical generalizability of DNV-TC classifications remains to be confirmed through (1) prospective application to independent patient cohorts with *de novo* molecular profiling; (2) external validation in datasets not used during framework development; and (3) ultimately, prospective interventional trials stratifying patients by DNV-TC signature. We explicitly caution against overinterpreting the current retrospective concordance analyses as evidence of clinical readiness.

Network incompleteness remains a fundamental challenge. Regulatory interactions are incompletely mapped, post-translational modifications are imperfectly characterized, and metabolic fluxes are difficult to measure. Improved experimental techniques including perturb-seq, genome-scale CRISPR screens with single-cell readouts, and advances in mass spectrometry-based proteomics will progressively fill the gaps, enabling higher-fidelity network reconstruction.

Tumor heterogeneity poses conceptual and practical challenges. A tumor is a heterogeneous ecosystem of subclones with different genetic and epigenetic profiles (McGranahan and Swanton, 2017). DNV-TC classification based on bulk tissue may miss critical intra-tumoral heterogeneity. Single-cell sequencing reveals that different regions or subclones may occupy different DNV-TC classes. Therapy targeting one subpopulation may select for the expansion of another with a different signature. Addressing this requires single-cell-resolution DNV-TC classification, which is computationally intensive but increasingly feasible with maturing technologies. Clonal evolution means DNV-TC signatures change over time, particularly under therapeutic pressure. Serial biopsies with classification at diagnosis, during treatment, and at progression could track evolutionary trajectories and predict emerging resistance.

Microenvironmental context represents another limitation. Current DNV-TC focuses on tumor cell-intrinsic network architecture, but tumor behavior critically depends on microenvironmental interactions including immune cells, cancer-associated fibroblasts, vascular endothelium, and the extracellular matrix. A tumor with high architectural vulnerability may resist therapy if surrounded by an immunosuppressive microenvironment preventing immune-mediated clearance. Conversely, immunotherapy can overcome distributed vulnerability by enlisting immune mechanisms attacking tumors through orthogonal mechanisms (Rizvi et al., 2015). Future DNV-TC iterations should incorporate tumor–microenvironment interactions, potentially adding axes for immune infiltration, stromal activation, and angiogenic potential.

The ultimate validation requires prospective clinical trials where patients are stratified by DNV-TC classification and treated according to the DNV-TC-informed strategies. For example, a melanoma trial could compare DNV-TC-guided therapy selection *versus* the physician’s choice with single-node vulnerable patients receiving monotherapy while distributed vulnerability patients receive combination therapy upfront. If DNV-TC guidance improves outcomes, this would constitute strong validation of clinical utility. Such trials are feasible since DNV-TC classification based on diagnostic biopsy material could be completed within 2–3 weeks using RNA sequencing and targeted DNA sequencing. Computational pipelines for automated classification need development and validation before clinical implementation. Regulatory approval pathways for network-based classifications remain uncertain and require engagement with regulatory agencies early in development.

A further limitation concerns the comparative validation against conventional classification systems. While the current study demonstrates that NV-class retains independent prognostic value after controlling for stage, grade, and age (HR 2.3, p < 0.001), and that MP-class outperforms TNM staging for metastasis prediction (ΔAUC = +0.13, p < 0.001), we were unable to test formal integrated multivariable models simultaneously incorporating the TNM stage, established molecular subtypes (e.g., PAM50 for breast cancer and CMS for colorectal cancer), and DNV-TC indices. Such analyses, reporting C-index improvement and likelihood ratio statistics, would provide a more rigorous quantification of the incremental clinical utility of DNV-TC beyond the current standard classifications. This constitutes a high-priority analytical objective for future prospective validation cohorts with complete clinicopathological annotation.

## 5 Conclusion

Cancer arises through the systematic reactivation of embryonic developmental programs reorganizing cellular regulatory networks around developmental master regulators and creating architectural vulnerabilities. DNV-TC classification captures this process through four complementary dimensions identifying which developmental programs are reactivated, quantifying network architectural vulnerability, characterizing dynamic response to perturbations, and predicting metastatic potential. By integrating developmental biology, network medicine, and dynamical systems theory, DNV-TC provides mechanistically grounded classification predicting therapeutic responses, explaining resistance mechanisms, and guiding rational combination therapy design.

Retrospective proof-of-concept analysis across well-characterized tumor types demonstrates strong concordance with published clinical outcomes. Network vulnerability architecture determines therapeutic response patterns, with single-node vulnerable tumors showing marked but sometimes transient responses, while distributed vulnerability tumors require combinations targeting multiple compensatory pathways. Cascade expansion dynamics predict response kinetics with hyper-cascading tumors responding rapidly but adapting quickly. Metastatic propensity reflects the reactivation of embryonic migration programs, enabling successful dissemination.

Future work must address limitations through comprehensive prospective validation, incorporation of tumor microenvironment and heterogeneity, development of clinical-grade computational pipelines, and clinical trials demonstrating improved outcomes with DNV-TC-guided therapy. If successful, this work could enable a paradigm shift from treating cancer as genetic disease to understanding it as a systems disease of network reorganization and from empirical therapy to rational network reprogramming.

## Data Availability

The datasets analyzed in this study are publicly available and no new primary data were generated. This study presents a theoretical classification framework validated using published summary data from existing literature. Somatic mutation data, RNA-seq expression profiles, Illumina 450K DNA methylation data and harmonized clinical outcome data were obtained from The Cancer Genome Atlas through the NCI Genomic Data Commons (https://portal.gdc.cancer.gov). Additional resources used were: COSMIC Cancer Gene Census v95 (https://cancer.sanger.ac.uk/census); STRING v11.5 (https://string-db.org); the ENCODE Project (https://www.encodeproject.org); ChIP-Atlas (https://chip-atlas.org); Mouse Genome Informatics (http://www.informatics.jax.org); gnomAD v2.1.1 (https://gnomad.broadinstitute.org); the UCSC Genome Browser (https://genome.ucsc.edu); KEGG (https://www.genome.jp/kegg); Reactome (https://reactome.org); and Recon3D (https://www.vmh.life). The mathematical framework and calculation procedures for T-DRI, TD-NV and CEI-T indices are fully specified in Methods and Supplementary Methods Sections S1–S3, enabling independent implementation in any programming environment. The classification criteria and complete signature gene lists are provided in Table 1 and Supplementary Table S6.
