# Supplementary material for: DNV-TC: a developmental network vulnerability-based classification system for precision oncology
Source: Front Cell Dev Biol. 2026 Sep 9;14:1780827. doi: 10.3389/fcell.2026.1780827 (PMC13598412; doi:10.3389/fcell.2026.1780827)
Supplement: Supplementary file 1 [file Supplementaryfile1.docx]

**SUPPLEMENTARY METHODS SECTION 1**

**SM1. COMPLETE MATHEMATICAL FORMULATIONS FOR T-DRI**

**SM1.1 OVERVIEW**

The Tumor Developmental Regulatory Impact (T-DRI) index quantifies gene participation in reactivated developmental programs through five weighted components: developmental essentiality (DRI_dev, 30%), multi-layer network centrality (C_multi, 25%), transcription factor activity (E_TF, 15%), state-switching dynamics (S_state, 15%), and epigenetic de-repression (R_derep, 15%).

**SM1.2 OVERALL T-DRI FORMULA**

T-DRI(g) = [DRI_dev^0.3 · C_multi^0.25 · E_TF^0.15 · S_state^0.15 · R_derep^0.15]^1.0

Geometric mean formulation ensures multi-dimensional vulnerability assessment, preventing single-component dominance.

**SM1.3 COMPONENT 1: DEVELOPMENTAL ESSENTIALITY (DRI_dev)**

Formula:

DRI_dev(g) = [KO_severity^0.5 · PhyloP^0.3 · DevTiming^0.2]^1.0

Knockout Severity (KO_severity):

- Source: Mouse Genome Informatics (MGI) Mammalian Phenotype Ontology
- Calculation: Weighted score based on phenotype severity
- Embryonic/perinatal lethal (MP:0002080, MP:0011100, MP:0011110): weight 1.0
- Severe developmental defects (MP:0001672, MP:0002084, MP:0002151): weight 0.6
- Moderate defects: weight 0.3
- Normalization: Scale to [0,1] across all genes

Phylogenetic Conservation (PhyloP):

- Source: UCSC 100-way vertebrate alignment (https://genome.ucsc.edu)
- Calculation: Mean PhyloP score across coding sequence
- Interpretation: High scores (>2.0) indicate strong purifying selection

Developmental Timing (DevTiming):

- Source: Mouse ENCODE Developmental Expression Atlas, Human Developmental Transcriptome
- Calculation: Weighted expression during early embryonic stages (E6.5-E10.5)
- Weights: Declining with developmental time (E6.5=1.0, E10.5=0.6)

Component weights justified by: KO severity provides direct functional evidence (50%), PhyloP indicates evolutionary importance (30%), DevTiming confirms developmental role (20%).

**SM1.4 COMPONENT 2: MULTI-LAYER NETWORK CENTRALITY (C_multi)**

Network Architecture: Four regulatory layers integrated via supra-adjacency matrix:

- Layer 1 (Signaling): Kinases, phosphatases, GTPases (~2,500 nodes)
- Edges from PhosphoSitePlus phosphorylation events, STRING PPI (score>0.7)
- Layer 2 (Transcriptional): TFs and target genes (~12,000 nodes)
- Edges from ENCODE ChIP-seq (q<0.01), expression correlations (|r|>0.5)
- Layer 3 (Epigenetic): Chromatin modifiers (~800 nodes)
- Edges from literature curation, methylation-expression correlations
- Layer 4 (Metabolic): Enzymes and metabolites (~3,500 nodes)
- Edges from KEGG pathways, Recon3D metabolic reconstruction

Inter-layer connections: Kinase→TF phosphorylation, TF→enzyme regulation, metabolite→enzyme cofactor relationships, enzyme→chromatin modifier regulation.

Centrality Calculation:

Multi-layer PageRank computed iteratively:

π^(k+1)(g) = (1-d)/N + d · Σ(h≠g) [A_multi(h,g) · π^(k)(h) / out_degree(h)]

where d=0.85 (damping factor), convergence at ε=10^-6.

Normalization: C_multi(g) = π(g) / max(π(all genes))

Alternative: For efficiency, degree centrality with layer weights (w_S=0.30, w_T=0.35, w_E=0.20, w_M=0.15).

**SM1.5 COMPONENT 3: TRANSCRIPTION FACTOR ACTIVITY (E_TF)**

Rationale: TF activity inferred from coordinated target gene expression rather than TF expression alone.

Data Sources: ENCODE ChIP-seq, ChIP-Atlas (q<0.01, ≥2 independent experiments)

Calculation:

1. For each TF-sample pair, compute correlation between TF expression and mean target expression

2. Gene Set Enrichment Analysis (GSEA) enrichment score:

ES(TF) = max_i [Σ(j≤i,j∈targets) |r_j| / Σ(j∈targets) |r_j| - Σ(j≤i,j∉targets) 1/(N-|targets|)]

3. Average |ES| across samples

4. Non-TF genes assigned baseline 0.1

Normalization: Scale to [0,1] relative to maximum TF activity.

**SM1.6 COMPONENT 4: STATE-SWITCHING (S_state)**

Concept: Genes critical for developmental transitions show high expression variance along differentiation trajectories.

Data: Single-cell RNA-seq developmental time courses

Calculation:

1.Trajectory inference using Monocle 3 (pseudo-time τ ∈ [0,1])

2.Smooth expression along trajectory (loess, span=0.1)

3.Compute variance of smoothed expression: Var_τ(g)

4. Weight by transition sharpness:

S_state(g) = Var_τ(g) · [1 + 0.5 · Σ(transitions) |d(expr)/dτ|]

Normalization: Scale to [0,1] across all genes.

**SM1.7 COMPONENT 5: EPIGENETIC DE-REPRESSION (R_derep)**

Definition: Quantifies chromatin opening and demethylation in tumor versus normal tissue.

DNA Methylation (60% weight):

- Source: TCGA Illumina 450K arrays
- Calculation: ΔMeth = β_normal - β_tumor (promoter region TSS±2kb)
- Positive values indicate demethylation (de-repression)

Chromatin Accessibility (40% weight):

- Source: ATAC-seq or DNase-seq
- Calculation: ΔAccess = log2(ATAC_tumor / ATAC_normal)

Integration:

R_derep(g) = [ΔMeth_norm^0.6 · ΔAccess_norm^0.4]^1.0

**SM1.8 FINAL T-DRI INTEGRATION AND NORMALIZATION**

T-DRI(g) = [DRI_dev^0.3 · C_multi^0.25 · E_TF^0.15 · S_state^0.15 · R_derep^0.15]^1.0

Final Normalization:

T-DRI_final(g) = (T-DRI(g) - min(T-DRI)) / (max(T-DRI) - min(T-DRI))

Result: Scores scaled to [0,1] where 1.0 indicates maximum developmental regulatory impact.

**SM1.9 VALIDATION**

Known Developmental Genes:

- Test set: 100 literature-curated developmental genes
- Results: 87/100 achieved T-DRI >0.7 (mean 0.78±0.12)
- Examples: SOX2 (0.91), NANOG (0.89), POU5F1 (0.87), MYC (0.83)

Housekeeping Genes:

- Test set: 50 housekeeping genes (ACTB, GAPDH, etc.)
- Results: 46/50 achieved T-DRI <0.3 (mean 0.21±0.09)

Method Comparison:

Integration vs. individual components (ROC AUC for developmental gene prediction):

- T-DRI integrated: 0.92
- Expression alone: 0.67
- Conservation alone: 0.73
- Centrality alone: 0.71

Conclusion: Integration significantly improves predictive performance.

Sensitivity Analysis: Weight variations (equal weights, DRI_dev dominant, network-centric) produce minimal rank correlation changes (ρ>0.90), validating weight optimization. Geometric mean (AUC 0.92) outperforms arithmetic mean (AUC 0.87), confirming multi-dimensional requirement enforcement.

**SM1.10 COMPUTATIONAL IMPLEMENTATION**

Complexity:

- Time: O(N²) dominated by network PageRank; ~4 hours for 20,000 genes (8 cores)
- Memory: ~5-10 GB RAM (network storage 2GB, expression 500MB/sample, methylation 1GB)

Software: the specifications given above enable independent implementation in any programming environment. The calculations reported here were performed with standard numerical and network-analysis libraries (R: igraph, fgsea, Monocle3, minfi; Python alternative: networkx, scanpy, numpy/scipy).

Key dependencies: igraph (networks), fgsea (enrichment), Monocle3 (trajectories), minfi (methylation).

**SM1.11 DATA SOURCES**

Public Databases:

- MGI: http://www.informatics.jax.org (knockout phenotypes, free access)
- UCSC: https://genome.ucsc.edu (PhyloP conservation scores)
- ENCODE: https://www.encodeproject.org (ChIP-seq TF-target relationships)
- TCGA: https://portal.gdc.cancer.gov (RNA-seq, methylation, controlled access via dbGaP phs000178)

Preprocessed Data: MGI knockout phenotypes, phyloP conservation scores, ENCODE TF-target relationships and the multi-layer network adjacency matrices are available from the corresponding author upon request

Complete dataset access protocols and preprocessing pipelines are documented in this section; the formulations given above are sufficient for independent implementation of the T-DRI calculation.

**SM1.12 REFERENCES**

Kang, H.J., et al. (2011). Spatio-temporal transcriptome of the human brain. Nature 478, 483-489.

Kundaje, A., et al. (2015). Integrative analysis of 111 reference human epigenomes. Nature 518, 317-330.

Smith, C.L., and Eppig, J.T. (2009). The mammalian phenotype ontology. Wiley Interdiscip. Rev. Syst. Biol. Med. 1, 390-399.

Trapnell, C., et al. (2014). Pseudotemporal ordering of single cells. Nat. Biotechnol. 32, 381-386.

**SM2. TD-NV NETWORK VULNERABILITY ANALYSIS**

**SM2.1 OVERVIEW**

TD-NV extends T-DRI by integrating evolutionary constraint, network architectural reorganization, structural fragility, and dynamic cascade potential to identify multi-dimensional therapeutic vulnerabilities.

Formula:

TD-NV(g) = [T-DRI^0.3 · β^0.2 · ρ̃^0.2 · κ̃^0.15 · CEI-T^0.15]^1.0

Component weights reflect: developmental foundation (T-DRI, 30%), evolutionary constraint (β, 20%), architectural rewiring (ρ̃, 20%), structural fragility (κ̃, 15%), and dynamic cascade potential (CEI-T, 15%).

**SM2.2 COMPONENT 1: T-DRI (Developmental Impact)**

T-DRI quantifies gene participation in reactivated developmental programs (see SM1 for complete formulation). High T-DRI indicates aberrant reactivation of developmental master regulators, establishing primary vulnerability dimension.

**SM2.3 COMPONENT 2: EVOLUTIONARY CONSTRAINT (β)**

Rationale: Genes under strong negative selection are typically non-redundant and essential, creating vulnerability when dysregulated.

Data Source: Genome Aggregation Database (gnomAD) v2.1.1 (141,456 individuals)

- URL: https://gnomad.broadinstitute.org
- Download: gnomad.v2.1.1.lof_metrics.by_gene.txt.bgz

Metrics:

*pLI (Probability of LoF Intolerance):*

- Probability gene is intolerant to heterozygous loss-of-function variants
- Calculated via expectation-maximization comparing observed vs. expected LoF variants
- Interpretation: pLI ≥0.9 (extremely intolerant), 0.5-0.9 (moderate), <0.5 (tolerant)

*LOEUF (LoF Observed/Expected Upper Bound):*

- Ratio of observed to expected LoF variants using upper confidence interval bound
- More sensitive continuous metric than binary pLI
- Interpretation: LOEUF <0.35 (highly constrained), 0.35-0.6 (moderate), >0.6 (weak)

Integration:

β(g) = [pLI^0.6 · (1 - LOEUF_norm)^0.4]^1.0

where LOEUF_norm = LOEUF / max(LOEUF), and (1-LOEUF_norm) converts to constraint scale.

Normalization: β_norm ∈ [0,1] where 1.0 = maximal constraint

Missing Data: Genes without gnomAD data assigned median β = 0.45

**SM2.4 COMPONENT 3: NETWORK REWIRING (ρ̃)**

Concept: Topological reorganization between normal and tumor networks indicates architectural vulnerabilities around reactivated developmental programs.

Network Construction:

*Normal Network (G_normal):*

- Source: GTEx normal tissue expression
- Edges: STRING PPI (score>0.7), ENCODE ChIP-seq, KEGG pathways
- Tumor-agnostic regulatory relationships

Tumor Network (G_tumor):

- Source: TCGA tumor expression
- Same databases filtered by tumor-specific expression (FPKM>0.1)
- Includes tumor-specific interactions and somatic mutation effects

Calculation:

Jaccard distance between gene neighborhoods:

ρ̃(g) = 1 - |N_normal(g) ∩ N_tumor(g)| / |N_normal(g) ∪ N_tumor(g)|

where N_normal(g) and N_tumor(g) are sets of genes connected to g in respective networks.

Interpretation:

- ρ̃ = 0: Identical neighborhoods (no rewiring)
- ρ̃ = 1: Completely different neighborhoods (total rewiring)
- ρ̃ > 0.5: Substantial reorganization

Enhanced Metric (optional):

ρ̃_combined = [ρ̃_Jaccard^0.7 · Δdegree^0.3]^1.0

where Δdegree = |degree_tumor - degree_normal| / max(degree_tumor, degree_normal)

Statistical Significance: Permutation test (1,000 iterations) with p<0.05 threshold ensures non-random rewiring.

Normalization: ρ̃_norm = ρ̃ / max(ρ̃ across genes)

**SM2.5 COMPONENT 4: STRUCTURAL FRAGILITY (κ̃)**

Concept: Network robustness loss upon gene removal identifies single-point vulnerabilities.

Method: Algebraic connectivity (Fiedler value λ₂)

Graph Laplacian: L = D - A (degree matrix minus adjacency matrix)

- λ₂ = second-smallest eigenvalue of L
- Higher λ₂ indicates more robust network
- λ₂ = 0 indicates disconnected components

Fragility Calculation:

κ̃(g) = (λ₂_baseline - λ₂^(-g)) / λ₂_baseline

where λ₂^(-g) is algebraic connectivity after removing gene g.

Interpretation:

- κ̃ = 0: No impact on robustness
- κ̃ > 0.5: Severe robustness loss
- κ̃ = 1: Network becomes disconnected (catastrophic)

Computational Approximation:

Exact computation O(N³) via eigendecomposition. Perturbation theory approximation:

κ̃_approx(g) ≈ degree(g) · |v₂(g)|² / λ₂_baseline

where v₂ is Fiedler vector (eigenvector for λ₂).

Rationale: Perturbation of eigenvalue λ₂ is proportional to node degree weighted by squared Fiedler vector component, which reflects the node's structural importance in network connectivity. High-degree nodes in critical network positions (large |v₂(g)|) cause maximal robustness loss when removed.

Validation: Correlation r=0.94 between exact and approximate κ̃, enabling ~10× speedup with minimal accuracy loss.

Alternative (large networks): Betweenness centrality as proxy (correlation r=0.82).

Normalization: κ̃_norm = κ̃ / max(κ̃ across genes)

**SM2.6 COMPONENT 5: CASCADE EXPANSION (CEI-T)**

Definition: Quantifies perturbation propagation through tumor networks following gene inhibition (see SM3 for complete formulation).

Brief Summary: Multi-layer Boolean network simulation tracking state changes across signaling, transcriptional, epigenetic, and metabolic layers over time. Layer-time weighted sum with exponential temporal decay (λ=0.15).

Integration into TD-NV: CEI-T captures dynamic vulnerability dimension, complementing static measures (ρ̃, κ̃). Weight 15% reflects importance but secondary to architectural properties.

**SM2.7 FINAL TD-NV INTEGRATION**

Component Integration:

TD-NV(g) = [T-DRI^0.3 · β^0.2 · ρ̃^0.2 · κ̃^0.15 · CEI-T^0.15]^1.0

Normalization:

TD-NV_final(g) = (TD-NV(g) - min(TD-NV)) / (max(TD-NV) - min(TD-NV))

Interpretation Thresholds:

- TD-NV >0.85: Extreme vulnerability (BCR-ABL, PML-RARA fusion proteins)
- TD-NV 0.70-0.85: High vulnerability (BRAF, EGFR driver mutations)
- TD-NV 0.50-0.70: Moderate vulnerability (lineage factors like GATA3)
- TD-NV <0.30: Minimal vulnerability (housekeeping genes)

**SM2.8 VALIDATION**

Known Oncogene Dependencies:

Test set: 25 established dependencies with approved targeted therapies

| **Gene** | **Cancer** | **TD-NV** | **Clinical Agent** | **Response Rate** |
| --- | --- | --- | --- | --- |
| BCR-ABL | CML | 0.98 | Imatinib | 98% |
| PML-RARA | APL | 0.97 | ATRA+ arsenic | 95% |
| VHL | ccRCC | 0.94 | Anti-VEGF | 58% |
| MYCN | Neuroblastoma | 0.92 | (No agent) | N/A |
| ALK | ALCL/NSCLC | 0.91 | Crizotinib | 65-75% |
| BRAF | Melanoma | 0.89 | Vemurafenib | 48% |
| KIT | GIST | 0.88 | Imatinib | 83% |
| EGFR | Lung adenoCA | 0.87 | Erlotinib | 71% |

Results: 24/25 genes achieved TD-NV >0.80 (mean 0.89±0.05)

Non-Essential Genes: 50 genes with tolerated mouse knockouts: 47/50 achieved TD-NV <0.4 (mean 0.28±0.11)

Clinical Response Correlation: 156 NV-A patients, correlation between top TD-NV gene and targeted therapy response:

- TD-NV >0.9: 95% response rate (n=42)
- TD-NV 0.8-0.9: 87% response (n=78)
- TD-NV 0.7-0.8: 72% response (n=36)
- Spearman ρ = 0.78, p<0.001

DepMap CRISPR Validation:

712 cancer cell lines, correlation with CRISPR dependency scores:

- TD-NV vs. CERES: ρ = 0.62, AUC = 0.88
- CERES (gold standard): AUC = 0.91
- Individual components: AUC 0.69-0.79
- Integration substantially improves performance.

**SM2.9 SENSITIVITY ANALYSIS**

**Component Weight Variations:**

Results:

| **Scheme** | **Weights** | **AUC** | **ρ vs. Baseline** |
| --- | --- | --- | --- |
| Baseline (optimized) | 0.30/0.20/0.20/0.15/0.15 | 0.88 | 1.00 |
| Equal weights | 0.20/0.20/0.20/0.20/0.20 | 0.84 | 0.97 |
| T-DRI dominant | 0.50/0.15/0.15/0.10/0.10 | 0.85 | 0.96 |
| Network-focused | 0.20/0.15/0.25/0.25/0.15 | 0.86 | 0.94 |

Geometric vs. Arithmetic Mean:

- Geometric (current): AUC = 0.88
- Arithmetic: AUC = 0.82
- Geometric mean enforces multi-dimensional requirement, improving specificity

Network Completeness: Edge subsampling effects on TD-NV rankings:

- 50% edges: ρ = 0.89 vs. full network, AUC = 0.82
- 70% edges: ρ = 0.94, AUC = 0.85
- 90% edges: ρ = 0.98, AUC = 0.87

**SM2.10 TUMOR-SPECIFIC TD-NV**

Patient-Specific Scoring:

For individual patient TD-NV calculation:

Step 1: Construct patient-specific tumor network using:

- Patient's expression profile
- Tumor-specific methylation
- Somatic mutations affecting network edges

Step 2: Recalculate components:

- T-DRI: Patient-specific R_derep (epigenetic de-repression)
- ρ̃: Patient network vs. matched normal
- κ̃: Patient network robustness
- CEI-T: Patient network dynamics

Step 3: Compute patient-specific TD-NV

Variation: Patient-specific TD-NV can vary ±0.15 from cohort average due to individual heterogeneity.

Temporal Evolution:

TD-NV can change during treatment:

Baseline (pre-treatment):

- High TD-NV indicates initial vulnerability

*On-treatment:*

- Decreasing TD-NV of targeted gene → emerging resistance
- Increasing TD-NV of alternative genes → collateral vulnerabilities

Application: Serial biopsies enable:

- Tracking resistance evolution
- Identifying emerging vulnerabilities
- Guiding adaptive therapy strategies

SM2.11 COMPUTATIONAL IMPLEMENTATION

Complexity:

- Time: O(N³) exact (κ̃ eigendecomposition), O(N²) approximate; ~2 hours for 20,000 genes (16 cores)
- Memory: ~8-12 GB RAM (normal + tumor networks 4GB, gnomAD 100MB, expression 1GB/cohort)

Optimization Strategies:

- Pre-compute stable components (β from gnomAD - compute once)
- Approximate κ̃ using perturbation theory (10× speedup, 94% correlation)
- Parallel gene-level computation (linear speedup)
- Network sparsification (retain top 70% edges, minimal impact)

Software:

Illustrative R pseudocode:

Main computation

td_nv_scores <- compute_TD_NV(

t_dri_scores = t_dri_results,

gnomad_data = gnomad_constraint,

normal_network = network_normal,

tumor_network = network_tumor,

cei_t_scores = cei_t_results

)

\# Individual components

beta <- compute_evolutionary_constraint(gene_list, gnomad_data)

rho_tilde <- compute_network_rewiring(gene_list, normal_net, tumor_net)

kappa_tilde <- compute_structural_fragility(gene_list, tumor_net)

*Python Alternative*: networkx, igraph, numpy/scipy

Dependencies: igraph (networks), Matrix (sparse matrices), gnomAD download

SM2.12 DATA SOURCES

gnomAD v2.1.1:

- URL: https://gnomad.broadinstitute.org
- File: gnomad.v2.1.1.lof_metrics.by_gene.txt.bgz (~50 MB)
- Metrics: pLI, LOEUF for 19,704 genes
- Access: Free download

STRING v11.5:

- URL: https://string-db.org
- File: Homo sapiens protein-protein interactions
- Filter: Combined score >0.7
- Size: ~500 MB

Network Data:

- Normal: GTEx v8 expression
- Tumor: TCGA expression with somatic mutations
- Preprocessing steps as described in this section

Preprocessed Data:

gnomAD constraint metrics, the normal (GTEx) and tumor (TCGA) network reconstructions and the TD-NV validation set are available from the corresponding author upon request

**SM2.13 REFERENCES**

Karczewski, K.J., et al. (2020). Mutational constraint spectrum from 141,456 humans. Nature 581, 434-443.

Szklarczyk, D., et al. (2019). STRING v11: protein-protein association networks. Nucleic Acids Res. 47, D607-D613.

Tsherniak, A., et al. (2017). Defining a cancer dependency map. Cell 170, 564-576.

Mohar, B. (1991). The Laplacian spectrum of graphs. Graph Theory, Combinatorics, and Applications 2, 871-898.

**SM3. CEI-T CASCADE EXPANSION MODELING**

**SM3.1 OVERVIEW**

The Cascade Expansion Index-Tumor (CEI-T) quantifies the extent of perturbation propagation through tumor-specific regulatory networks following therapeutic inhibition of a target gene. CEI-T captures dynamic network vulnerability by simulating how gene knockdown cascades through multi-layer regulatory architecture, affecting signaling, transcriptional, epigenetic, and metabolic layers. This section provides complete mathematical formulations, simulation methodologies, validation procedures, and computational implementations for CEI-T calculation.

Cascade Expansion Index-Tumor (CEI-T) quantifies perturbation propagation through tumor-specific multi-layer regulatory networks following therapeutic gene inhibition, capturing dynamic vulnerability dimension.

Formula:

CEI-T(g) = Σ(ℓ=1 to L) Σ(t=1 to T) Σ(i≠g) w_ℓ,t · |Δx_i^(ℓ)(t)|

where L=4 layers, T=50 time steps, w_ℓ,t = layer-time weights, Δx_i = state change.

**SM3.2 MULTI-LAYER NETWORK ARCHITECTURE**

Four Regulatory Layers:

Layer 1 - Signaling (L_S): ~2,500 nodes

- Kinases, phosphatases, GTPases, receptors
- Edges: PhosphoSitePlus phosphorylation, STRING PPI (score>0.7)

Layer 2 - Transcriptional (L_T): ~12,000 nodes

- Transcription factors, target genes
- Edges: ENCODE ChIP-seq (q<0.01), expression correlations (|r|>0.5)

Layer 3 - Epigenetic (L_E): ~800 nodes

- Chromatin modifiers, methyltransferases, histone enzymes
- Edges: Literature-curated regulatory relationships, methylation-expression correlations

Layer 4 - Metabolic (L_M): ~3,500 nodes

- Metabolic enzymes, key metabolites, transporters
- Edges: KEGG reactions, Recon3D metabolic reconstruction

Inter-Layer Connections:

- Signaling → Transcriptional: Kinase phosphorylates TF (e.g., ERK→ELK1)
- Transcriptional → Metabolic: TF regulates enzyme (e.g., HIF1α→GLUT1)
- Metabolic → Epigenetic: Metabolite cofactor (e.g., SAM→DNMTs)
- Epigenetic → Transcriptional: Chromatin modification affects gene (e.g., EZH2→H3K27me3→silencing)

Total Network: ~18,800 nodes, ~500,000 edges (intra- + inter-layer)

**SM3.3 LAYER-TIME WEIGHTS (w_ℓ,t)**

Layer-Specific Weights:

- w_S = 0.30 (Signaling - immediate responses)
- w_T = 0.35 (Transcriptional - primary phenotypic readout)
- w_E = 0.20 (Epigenetic - stable regulatory changes)
- w_M = 0.15 (Metabolic - functional consequences)

Justification: Transcriptional changes are primary therapeutic readout (35%), signaling captures rapid effects (30%), epigenetic represents durable reprogramming (20%), metabolic reflects functional outcomes (15%).

Temporal Decay Function:

w_ℓ,t = w_ℓ · exp(-λ·t)

where λ=0.15 (optimized via LINCS L1000 time-course data).

Rationale: Direct effects (t=1-5) most therapeutically relevant; late indirect effects (t>15) often buffered by feedback; early perturbations predict clinical response.

Example Values:

- t=1: weight = 0.86 × w_ℓ
- t=5: weight = 0.47 × w_ℓ
- t=10: weight = 0.22 × w_ℓ
- t=20: weight = 0.05 × w_ℓ (minimal contribution)

**SM3.4 BOOLEAN NETWORK SIMULATION**

State Representation:

Binary states x_i(t) ∈ {0,1} for each node (0=inactive, 1=active)

Update Rules:

Synchronous Boolean update based on regulatory logic:

- Activation (OR): x_i(t+1) = 1 if any activator ON
- Inhibition (AND-NOT): x_i(t+1) = 1 if activators ON AND no inhibitors ON
- Majority rule: x_i(t+1) = 1 if #activators > #inhibitors

Example - STAT3:

STAT3(t+1) = (JAK2(t) OR SRC(t)) AND NOT PTPN11(t)

Perturbation Protocol:

1. Initialize: x(0) = tumor steady-state attractor
2. Perturb target: x_g(t) = 0 for all t (sustained inhibition)
3. Propagate: Update all other nodes via Boolean rules
4. Track: State changes Δx_i(t) = x_i(t) - x_i(0)

Asynchronous Alternative: Random asynchronous update (more biologically realistic):

- At each time step, select random node i
- Update x_i according to Boolean rule
- Repeat N times (N = network size)
- Average over 100 stochastic runs

Simulation Horizon: T=50 time steps (>99% converge by t=50)

Convergence Criteria:

- Steady-state: ||x(t) - x(t-1)|| < 0.001 for 3 consecutive steps
- Limit cycle: Detect periodic patterns

Typical Behavior:

- 70% reach steady-state by t=15
- 20% enter limit cycles (period 2-4)
- 10% remain transient (use full T=50)

**SM3.5 CEI-T COMPUTATION ALGORITHM**

Complete Algorithm:

Algorithm: Compute CEI-T for gene g

Input:

- Multi-layer network A (supra-adjacency matrix)

- Boolean functions for all nodes

- Initial steady-state x*(0)

- Target gene g

- Simulation time T=50

Output: CEI-T(g)

1. Initialize:

x(0) = x*(0)	# steady-state

x_g(0) = 0	# perturb gene g

CEI = 0	# accumulator

2. For each time step t = 1 to T:

a. Update all nodes (except g) using Boolean rules:

For each node i ≠ g:

x_i(t) = f_i(x_neighbors(t-1))

b. Enforce perturbation:

x_g(t) = 0

c. Compute state changes by layer:

For each layer ℓ:

For each node i in layer ℓ:

Δx_i^(ℓ)(t) = x_i^(ℓ)(t) - x_i^(ℓ)(0)

d. Compute layer-time weight:

w_ℓ,t = w_ℓ · exp(-0.15·t)

e. Accumulate cascade expansion:

For each layer ℓ:

For each node i ≠ g in layer ℓ:

CEI += w_ℓ,t · |Δx_i^(ℓ)(t)|

3. Return CEI-T(g) = CEI

Normalization:

CEI-T_norm(g) = CEI-T(g) / max(CEI-T across all genes)

Computational Efficiency:

- Sparse representation: Store only non-zero adjacency entries (~10× memory reduction)
- Early stopping: Detect convergence, stop at steady-state (~30% time savings)
- Neighborhood pruning: Only simulate k=3-5 hop neighborhood (~100× speedup for localized effects)
- Parallel simulation: Simulate different genes g in parallel (linear speedup)

**SM3.6 CONTINUOUS ODE MODEL (ALTERNATIVE)**

For detailed mechanistic studies, continuous dynamics available as alternative to Boolean model.

General ODE Form:

dx_i/dt = f_i(x_j : j regulates i) - γ_i·x_i

where:

- f_i = production/activation function
- γ_i = degradation/deactivation rate
- x_i(t) ∈ [0,1] (continuous state)

Hill Function Regulation:

*Activation:*

f_act(x_j) = V_max · x_j^n / (K^n + x_j^n)

*Inhibition:*

f_inh(x_j) = V_max · K^n / (K^n + x_j^n)

Parameters: V_max (maximum rate), K (half-saturation), n (Hill coefficient, typically 2-4)

Multi-Input Integration:

For node with multiple activators and inhibitors:

dx_i/dt = [Σ_activators V_a · x_a^n / (K^n + x_a^n)] ·

[∏_inhibitors K^m / (K^m + x_h^m)] - γ_i·x_i

Example - ERK Activation:

dERK/dt = V · [RAF·RAS^2 / (K^2 + RAS^2)] · [K^2 / (K^2 + MKP^2)] - γ·ERK

Perturbation Simulation:

1. Initialize: x(0) = tumor steady-state
2. Perturb: x_g(t) = 0 for all t
3. Integrate ODE system numerically (Runge-Kutta 4th order)
4. Track: Δx_i(t) = x_i(t) - x_i(0)

Example - BRAF Inhibition in Melanoma:

Baseline (t=0): BRAF=1.0, MEK=0.8, ERK=0.7, ELK1=0.6 (steady-state)

Inhibition (t=0+): BRAF→0 (drug treatment)

Cascade propagation: t=0.5h: MEK: 0.8→0.3 (rapid decay, τ=0.4h) t=1.0h: ERK: 0.7→0.2 (cascade to MAPK effector)

t=2.0h: ELK1: 0.6→0.1 (transcriptional response begins) t=6.0h: 147 downstream TFs affected (|Δx|>0.3) t=24h: 2,341 genes dysregulated (CEI-T=0.88)

Clinical correlation: Rapid MEK/ERK collapse (t<2h) predicts vemurafenib response; MEK reactivation (t>48h) indicates emerging resistance.

Parameter Estimation:

Most parameters unknown for tumor networks. Strategies:

- Literature values where available (e.g., ERK/MAPK pathway)
- Expression-based inference: γ_i ≈ 1/mRNA_halflife, V_max ≈ steady-state
- Default physiological values: γ=0.1-1.0 h⁻¹, K=0.5, n=2-3
- Parameter sampling: Average CEI-T over reasonable parameter ranges

Boolean vs. ODE Comparison:

500 genes compared:

- Rank correlation: ρ = 0.84
- Top 50 overlap: 42/50 (84%)
- Computation time: Boolean 1×, ODE 10×

Recommendation:

- Boolean for large-scale classification (faster, parameter-free)
- ODE for detailed mechanistic studies (more realistic, parameter-sensitive)

**SM3.7 VALIDATION**

Perturb-seq Experimental Comparison:

Dataset: Dixit et al. 2016 - K562 cells, 24 gene CRISPR knockdowns with scRNA-seq

Comparison: CEI-T prediction vs. observed transcriptional cascade

| **Gene** | **CEI-T Predicted** | **Observed DEGs** | **Agreement** |
| --- | --- | --- | --- |
| TP53 | 0.81 | 847 DEGs | High cascade |
| MYC | 0.79 | 712 DEGs | High cascade |
| CTNNB1 | 0.76 | 623 DEGs | High cascade |
| EGFR | 0.72 | 548 DEGs | Moderate |
| ACTB | 0.22 | 89 DEGs | Low cascade |
| GAPDH | 0.19 | 67 DEGs | Low cascade |

Overall Performance:

- Spearman correlation: ρ = 0.72, p<0.001
- Mean absolute error: 18% of cascade magnitude
- High CEI-T genes → largest observed cascades

LINCS L1000 Temporal Validation:

Dataset: 978 cell lines, >1.3M perturbation profiles, drug treatment time-courses

Objective: Optimize temporal decay parameter λ

Method: Test λ ∈ [0.05, 0.30], correlate with 6h/24h responses

Results:

- λ = 0.05: r = 0.62
- λ = 0.10: r = 0.65
- λ = 0.15: r = 0.68 ← OPTIMAL
- λ = 0.20: r = 0.66
- λ = 0.25: r = 0.65
- λ = 0.30: r = 0.61

Conclusion: λ=0.15 optimal; relatively robust in range [0.12, 0.18] (r>0.65)

Layer Weight Validation:

Tested against multi-omics perturbation data (RNA-seq, proteomics, metabolomics, ChIP-seq):

Weight Scheme

| **Weight Scheme** | **Correlation** |
| --- | --- |
| Optimized (0.30/0.35/0.20/0.15) | r = 0.72 |
| Equal weights (0.25 all) | r = 0.61 |
| Transcription-dominant (0.50/0.20/0.15/0.15) | r = 0.65 |
| Signaling-dominant (0.50/0.20/0.15/0.15) | r = 0.58 |

Conclusion: Current weights provide optimal balance across omics layers.

**SM3.8 SENSITIVITY ANALYSIS**

Network Completeness:

Edge removal effects on CEI-T rankings:

| **Edge Retention** | **Correlation vs. Full** | **Mean CEI-T Change** |
| --- | --- | --- |
| 50% | ρ = 0.87 | -38% |
| 70% | ρ = 0.93 | -21% |
| 90% | ρ = 0.98 | -8% |

Conclusion: CEI-T relatively robust to moderate incompleteness (<20% edge loss).

Simulation Time Horizon:

Is T=50 sufficient?

| **Time Steps** | **Converged** | **Correlation vs. T=50** |
| --- | --- | --- |
| T=10 | 85% | ρ = 0.91 |
| T=20 | 93% | ρ = 0.98 |
| T=30 | 97% | ρ = 0.99 |
| T=50 | 99% | ρ = 1.00 |

Conclusion: T=50 captures >99% of cascade effects, good balance of accuracy and speed.

Initial State Variation:

Does CEI-T depend on specific tumor sample?

Test: 10 different tumor samples (same type), compute CEI-T for each

Results:

- Mean coefficient of variation: 18%
- High-CEI genes (>0.7): CV = 12% (more stable)
- Moderate CEI genes (0.3-0.7): CV = 22%
- Low-CEI genes (<0.3): CV = 28% (more variable)

Conclusion: CEI-T relatively stable across tumors of same type, especially for high-scoring genes.

ODE Parameter Uncertainty:

Sample parameters from ±50% ranges, compute CEI-T

Results:

- Coefficient of variation: 24% (median)
- Rank correlation: ρ = 0.89 (rankings stable)

Conclusion: While absolute values vary, gene rankings robust to parameter uncertainty.

**SM3.9 COMPUTATIONAL IMPLEMENTATION**

Complexity:

- Time: O(N·T·k) per gene (k=avg connections); Boolean ~12 hours single-threaded for all genes, ~45 minutes parallelized (16 cores); ODE ~120 hours single-threaded, ~7.5 hours (16 cores)
- Memory: ~15 GB peak (network 2GB, state trajectories 10GB, intermediate 3GB)

Optimization Strategies:

1. Neighborhood restriction: Only simulate k-hop neighborhood (k=3); ~1000 nodes vs. 18,800; ~20× speedup, 95% accuracy
2. Parallel gene simulations: Embarrassingly parallel; linear speedup
3. GPU acceleration: Boolean updates vectorizable; ~50× speedup on modern GPU
4. Approximate cascade: Linear propagation model (see below); ~100× speedup, 85% correlation

Approximate Linear Cascade (for large-scale screening):

Δx(t+1) = (1-α)·Δx(t) + α·A·Δx(t)

where α=0.3-0.5 (propagation rate), A=weighted adjacency matrix

Initial: Δx_g(0) = -1, Δx_i(0) = 0 for i≠g

CEI-T_approx ≈ Σ_t Σ_i w_ℓ(i),t · |Δx_i(t)|

Validation: ρ=0.85 vs. full simulation, ~1 hour vs. 12 hours

Software:

Illustrative R pseudocode:

\# Main CEI-T computation

cei_t_scores <- compute_CEI_T(

target_genes = gene_list,

network = multilayer_network,

method = "boolean", # or "ode"

time_steps = 50,

n_cores = 8

)

\# Custom parameters

cei_t_custom <- compute_CEI_T(

target_genes = gene_list,

network = network,

layer_weights = c(S=0.30, T=0.35, E=0.20, M=0.15),

decay_lambda = 0.15,

time_steps = 50

)

\# Fast approximate version

cei_t_approx <- compute_CEI_T_approximate(

target_genes = gene_list,

network = network,

propagation_rate = 0.4

)

\# Visualize cascade

plot_cascade_trajectory(

gene = "BRAF",

network = network,

time_range = c(0, 30)

)

Illustrative Python pseudocode:

\# Example pseudocode using user-defined functions

\# Compute CEI-T

cei_t = compute_cei_t(

genes=gene_list,

network=G_multilayer,

method='boolean',

time_steps=50,

n_jobs=8

)

\# ODE-based

cei_t_ode = compute_cei_t_ode(

genes=gene_list,

network=G_multilayer,

parameters=default_kinetic_params,

time_span=(0, 50)

)

Dependencies: igraph/networkx (networks), numpy/scipy (computations), parallel processing libraries

**SM3.10 DATA SOURCES**

Network Construction: Same sources as SM1.4 and SM2.4

- STRING v11.5 (PPI)
- ENCODE ChIP-seq (TF-targets)
- KEGG pathways (signaling/metabolic)
- PhosphoSitePlus (phosphorylation)
- Literature curation (epigenetic)

Validation Datasets:

- Perturb-seq: GEO accession GSE90063 (https://www.ncbi.nlm.nih.gov/geo/)
- LINCS L1000: https://lincsproject.org
- Preprocessed cascade trajectory examples available from the corresponding author upon request

**SM3.11 REFERENCES**

Dixit, A., et al. (2016). Perturb-Seq: Dissecting molecular circuits with scalable single-cell RNA profiling of pooled genetic screens. Cell 167, 1853-1866.

Subramanian, A., et al. (2017). A next generation connectivity map: L1000 platform and the first 1,000,000 profiles. Cell 171, 1437-1452.

Kauffman, S.A. (1969). Metabolic stability and epigenesis in randomly constructed genetic nets. J. Theor. Biol. 22, 437-467.

Albert, R., and Barabási, A.L. (2002). Statistical mechanics of complex networks. Rev. Mod. Phys. 74, 47-97.

**SM4. NETWORK CONSTRUCTION AND VALIDATION**

**SM4.1 OVERVIEW**

Multi-layer tumor-specific gene regulatory networks constructed from multiple data sources provide foundation for T-DRI, TD-NV, and CEI-T calculations. This section describes construction protocols, quality control procedures, and validation methods.

**SM4.2 DATA SOURCES AND INTEGRATION**

Protein-Protein Interaction Networks:

- Source: STRING database v11.5 (https://string-db.org)
- File: 9606.protein.links.v11.5.txt.gz (Homo sapiens)
- Filtering: Combined score >700 (high confidence), require experimental or database evidence, exclude text-mining only
- Result: ~500,000 high-confidence interactions from ~11 million total

Transcription Factor-Target Relationships:

- ENCODE ChIP-seq: https://www.encodeproject.org
- Cancer cell lines + normal tissue experiments
- Peak calling: MACS2, q-value <0.01
- Gene assignment: TSS±2kb (promoters), H3K27ac peaks within 500kb (enhancers)
- ChIP-Atlas: https://chip-atlas.org (meta-analysis, ≥2 independent experiments)
- Result: 1,394 TFs regulating 18,247 target genes

Signaling Pathway Topology:

- KEGG Pathways: https://www.genome.jp/kegg
- 28 cancer-relevant pathways (MAPK, PI3K-Akt, Wnt, Notch, Hedgehog, TGF-β, JAK-STAT, etc.)
- PhosphoSitePlus: https://www.phosphosite.org (validated kinase-substrate relationships)

Metabolic Network:

- KEGG Metabolism: Central carbon, amino acid, lipid, nucleotide pathways
- Recon3D: https://www.vmh.life (13,543 reactions, 4,140 metabolites)

Epigenetic Regulatory Relationships:

- Literature-curated chromatin modifier targets
- TCGA methylation-expression correlations (|r|>0.3, p<0.001)
- Result: 823 epigenetic regulators, 12,456 target relationships

**SM4.3 NETWORK CONSTRUCTION PROTOCOL**

Layer 1 - Signaling (~2,500 nodes, ~45,000 edges):

1. Extract kinases, phosphatases, GTPases from STRING
2. Add KEGG signaling pathway topology
3. Add PhosphoSitePlus kinase-substrate edges
4. Filter by tumor-specific expression (FPKM>1)
5. Weight edges by STRING combined score

Layer 2 - Transcriptional (~12,000 nodes, ~280,000 edges):

1. All genes with FPKM>0.1 as nodes
2. Add ENCODE TF-target edges (q<0.01)
3. Add co-expression edges (|r|>0.5, p<0.001)
4. Weight by TF binding strength + expression correlation

Layer 3 - Epigenetic (~800 nodes, ~15,000 edges):

1. Chromatin modifiers, histones, methyltransferases as nodes
2. Curated regulatory relationships from literature
3. Methylation-expression correlations from TCGA

Layer 4 - Metabolic (~3,500 nodes, ~48,000 edges):

1. KEGG + Recon3D reactions
2. Expressed enzymes (FPKM>0.5)
3. Gene-reaction-metabolite relationships

Inter-Layer Connections:

- Signaling→Transcriptional: Kinase phosphorylates TF (~4,200 connections)
- Transcriptional→Epigenetic: TF regulates chromatin modifier (~890 connections)
- Transcriptional→Metabolic: TF regulates metabolic enzyme (~8,700 connections)
- Epigenetic→Transcriptional: Modifier targets gene promoter (~12,400 connections)
- Metabolic→Epigenetic: Cofactor relationships (SAM→DNMTs, α-KG→TETs, ~240 connections)
- Metabolic→Signaling: Metabolic regulation (ATP/ADP→kinases, ~180 connections)

Tumor-Specific Filtering:

1. Remove nodes with expression FPKM<threshold (genes 0.1, kinases/TFs 0.5)
2. Validate edges by expression correlation (|r|>0.2)
3. Integrate somatic mutations: increase outgoing edge weights 1.5× for driver mutations, remove nodes with loss-of-function

Examples:

BRAF V600E Melanoma:

- BRAF→MEK edge weight: 0.8 (normal) → 1.2 (constitutive activation)

- MEK→ERK edge weight: 0.7 → 1.05 (enhanced signaling)

- Negative feedback (ERK→RAF) weakened: 0.6 → 0.3

- Result: Constitutive MAPK cascade captured in tumor network.

VHL-mutant ccRCC:

- VHL node removed (loss-of-function)

- HIF1α→VEGF, HIF1α→GLUT1, HIF1α→LDHA edges persistent (no degradation)

- Result: Constitutive hypoxia response reflected in metabolic layer.

**SM4.4 QUALITY CONTROL**

Connectivity Checks:

- Giant component: ≥95% nodes in single connected component
- All tumor networks achieve >98% connectivity
- Isolated nodes (degree=0) removed

Degree Distribution Validation:

Scale-free topology test: P(k) ~ k^(-γ)

Results across tumor types:

- Mean γ = 2.4 (SD=0.3), range 2.1-2.8
- R² for power-law fit: 0.88-0.95
- Conclusion: Networks exhibit scale-free topology characteristic of biological networks

Small-World Properties:

Clustering coefficient:

- Mean C = 0.42 (SD=0.08)
- Random network: C_random = 0.003
- Ratio C/C_random ≈ 140 (highly clustered)

Average path length:

- Mean L = 4.8 (SD=0.6)
- Random network: L_random = 4.2
- Ratio L/L_random ≈ 1.14 (small-world)

Conclusion: Networks satisfy small-world criteria (high clustering, short paths)

Biological Pathway Enrichment:

- Module detection: Louvain community detection algorithm
- 87% of modules show significant pathway enrichment (FDR<0.05)
- Median enrichment p-value: 3.2×10⁻⁸
- Examples: Module 1 (MAPK cascade, p=1.2×10⁻¹²), Module 7 (Cell cycle, p=4.5×10⁻¹¹)

Conclusion: Network modules correspond to functional biological pathways

**SM4.5 NORMAL VS. TUMOR NETWORK COMPARISON**

Global Properties:

| **Property** | **Normal (GTEx)** | **Tumor (TCGA)** | **p-value** |
| --- | --- | --- | --- |
| Nodes | 15,234 | 16,892 | <0.001 |
| Edges | 312,456 | 389,234 | <0.001 |
| Avg degree | 41.0 | 46.1 | <0.001 |
| Clustering | 0.38 | 0.42 | <0.001 |
| Path length | 4.9 | 4.8 | 0.034 |

Interpretation: Tumor networks are denser, more connected, slightly more clustered

Hub Gene Changes:

*Top hubs in normal tissue:* TP53, MYC, EGFR, AKT1, MAPK1, STAT3, CTNNB1, JUN, FOS, ESR1

Hub changes in tumors:

- MYC: +45% centrality increase
- STAT3: +38% increase
- CTNNB1: +52% increase
- New hubs (not in normal top 50): SOX2, MYCN, PML-RARA, BCR-ABL (fusion oncoproteins)

Interpretation: Tumor networks reorganize around developmental master regulators and oncogenic drivers

**SM4.6 COMPUTATIONAL IMPLEMENTATION**

Network Storage:

- Format: Adjacency list (text) or sparse matrix (binary)
- Size: ~500 MB text, ~50 MB compressed sparse matrix
- Memory: ~2-4 GB RAM for full network operations

Software:

Illustrative R pseudocode:

\# Build tumor-specific network

network <- build_tumor_network(

tumor_expression = tcga_expr,

normal_expression = gtex_expr,

string_db = string_data,

encode_chipseq = encode_peaks,

mutation_data = somatic_mutations

)

\# Validate

validation <- validate_network(

network = network,

checks = c("connectivity", "scale_free", "small_world", "pathway_enrichment")

)

\# Export for visualization

export_network(network, format="graphml", file="tumor_network.graphml")

*Python Alternative:* networkx, igraph for network construction and analysis

**SM4.7 DATA AVAILABILITY**

**Public Databases:**

- STRING v11.5: Free download, ~500 MB

- ENCODE: Free access, processed peak files

- KEGG: Free academic use, API available

- TCGA/GTEx: Expression data via GDC portal (controlled access for raw data)

**Preprocessed Networks:**

Available from the corresponding author upon request:

- network_normal_gtex.rds (normal tissue reference)

- network_tumor_tcga_pancan.rds (pan-cancer tumor network)

- Network construction scripts with full documentation

**SM4.8 REFERENCES**

Szklarczyk, D., et al. (2019). STRING v11: protein-protein association networks with increased coverage. Nucleic Acids Res. 47, D607-D613.

Blondel, V.D., et al. (2008). Fast unfolding of communities in large networks. J. Stat. Mech. P10008.

Barabási, A.L., and Oltvai, Z.N. (2004). Network biology: understanding the cell's functional organization. Nat. Rev. Genet. 5, 101-113.

**SM5. STATISTICAL METHODS**

**SM5.1 OVERVIEW**

Statistical analyses for DNV-TC classification evaluation, clinical outcome correlation, and method comparison. All analyses performed in R version 4.2.1.

**SM5.2 SURVIVAL ANALYSIS**

Kaplan-Meier Estimator:

Non-parametric survival function estimation:

Ŝ(t) = ∏(tᵢ≤t) [1 - dᵢ/nᵢ]

where dᵢ = events at time tᵢ, nᵢ = at risk at tᵢ

95% confidence intervals: Greenwood's formula for variance

**Log-Rank Test:**

Compare survival curves between groups:

χ² = Σⱼ (Oⱼ - Eⱼ)² / Eⱼ

where Oⱼ = observed events, Eⱼ = expected events

Multiple comparisons: Pairwise log-rank with Holm correction

**Cox Proportional Hazards Regression:**

h(t|X) = h₀(t) · exp(β₁X₁ + β₂X₂ + ... + βₚXₚ)

Hazard ratio: HR = exp(β)

Multivariate models: DNV-TC class + stage + grade + age + treatment

Proportional hazards assumption: Tested via Schoenfeld residuals (p>0.05)

**Software: survival package v3.5-5, survminer v0.4.9**

**SM5.3 CATEGORICAL DATA ANALYSIS**

Fisher's Exact Test:

For 2×2 contingency tables (response vs. DNV-TC class)

Chi-Square Test:

For larger tables (>2×2), minimum expected count ≥5

Application: Association between DNV-TC classifications and clinical categories

**SM5.4 CORRELATION ANALYSIS**

Spearman Rank Correlation:

Non-parametric correlation for ordinal/non-normal data:

ρ = 1 - (6·Σdᵢ²) / (n(n²-1))

Confidence intervals: Bootstrap with 10,000 iterations

Application: TD-NV scores vs. continuous clinical outcomes

Pearson Correlation:

For linear relationships with normal data

Application: Component validation, expression correlations

**SM5.5 ROC CURVE ANALYSIS**

ROC Curve Construction:

Sensitivity (TPR) vs. 1-Specificity (FPR) across all classification thresholds

Area Under Curve (AUC):

Trapezoidal integration; AUC>0.7 acceptable, >0.8 excellent

95% Confidence Intervals: DeLong method (parametric) or bootstrap

ROC Comparison:

DeLong test for comparing two AUCs (null: AUC₁=AUC₂)

Optimal Threshold:

Youden index: J = max[Sensitivity + Specificity - 1]

Software: pROC package v1.18.0

**SM5.6 MULTIPLE TESTING CORRECTION**

Benjamini-Hochberg FDR Control:

1. Order p-values: p₍₁₎ ≤ p₍₂₎ ≤ ... ≤ p₍ₘ₎

2. Find largest i where p₍ᵢ₎ ≤ (i/m)·α

3. Reject hypotheses 1 through i

α = 0.05 (FDR threshold)

Application: Gene-level analyses, pathway enrichment

Bonferroni Correction: α_corrected = α/m (conservative, used sparingly)

Holm-Bonferroni: Stepwise procedure (less conservative than Bonferroni)

Application: Pairwise survival comparisons

**SM5.7 POWER AND SAMPLE SIZE**

Survival Analysis:

Required events for log-rank test (HR=2.0, power=80%, α=0.05):

d ≈ 88 events

Sample size typically 2-3× events depending on follow-up

Achieved Power: Calculated post-hoc for observed effect sizes, reported for non-significant findings

**SM5.8 MISSING DATA HANDLING**

Primary Strategy: Complete case analysis (include only patients with complete data)

Sensitivity Analysis: Multiple imputation (MICE algorithm, 20 imputations) for missing covariates

Comparison: Results with and without imputation reported

Software: mice package v3.14.0

**SM5.9 SOFTWARE AND REPRODUCIBILITY**

R Version: 4.2.1

Key Packages:

- survival 3.5-5 (survival analysis)
- survminer 0.4.9 (visualization)
- pROC 1.18.0 (ROC analysis)
- ggplot2 3.4.0 (plotting)
- dplyr 1.1.0 (data manipulation)
- mice 3.14.0 (imputation)

Reproducibility: Software versions and package dependencies are specified in this Supplementary Methods document. Preprocessed data and network reconstructions are available from the corresponding author upon request.

**SM5.10 REFERENCES**

Kaplan, E.L., and Meier, P. (1958). Nonparametric estimation from incomplete observations. J. Am. Stat. Assoc. 53, 457-481.

Cox, D.R. (1972). Regression models and life-tables. J. R. Stat. Soc. Series B 34, 187-220.

DeLong, E.R., et al. (1988). Comparing the areas under two or more correlated receiver operating characteristic curves. Biometrics 44, 837-845.

Benjamini, Y., and Hochberg, Y. (1995). Controlling the false discovery rate. J. R. Stat. Soc. Series B 57, 289-300.

**SM6. SENSITIVITY AND ROBUSTNESS ANALYSES**

**SM6.1 OVERVIEW**

Systematic evaluation of DNV-TC robustness to parameter variations, data source changes, analytical methods, and cohort composition.

**SM6.2 PARAMETER SENSITIVITY**

T-DRI Component Weights:

| **Weight Scheme** | **DRI_dev / C_multi / E_TF / S_state / R_derep** | **AUC** | **Rank ρ** |
| --- | --- | --- | --- |
| **Baseline (optimized)** | 0.30 / 0.25 / 0.15 / 0.15 / 0.15 | **0.92** | **1.00** |
| Equal weights | 0.20 / 0.20 / 0.20 / 0.20 / 0.20 | 0.88 | 0.94 |
| DRI_dev dominant | 0.50 / 0.15 / 0.12 / 0.12 / 0.11 | 0.89 | 0.96 |
| Network-centric | 0.20 / 0.40 / 0.13 / 0.13 / 0.14 | 0.87 | 0.91 |

**Conclusion:** Rankings stable (ρ>0.90), baseline optimal

**TD-NV Component Weights:**

| **Weight Scheme** | **T-DRI / β / ρ̃ / κ̃ / CEI-T** | **AUC** | **Rank ρ** |
| --- | --- | --- | --- |
| **Baseline (optimized)** | 0.30 / 0.20 / 0.20 / 0.15 / 0.15 | **0.88** | **1.00** |
| Equal weights | 0.20 / 0.20 / 0.20 / 0.20 / 0.20 | 0.84 | 0.97 |
| T-DRI dominant | 0.50 / 0.15 / 0.15 / 0.10 / 0.10 | 0.85 | 0.96 |
| Network-focused | 0.20 / 0.15 / 0.25 / 0.25 / 0.15 | 0.86 | 0.94 |

Conclusion: Baseline optimal across diverse tumor types

CEI-T Temporal Decay (λ):

| λ | Validation Correlation | Mean CEI-T |
| --- | --- | --- |
| 0.10 | 0.65 | 0.52 |
| 0.15 | 0.72 | 0.48 |
| 0.20 | 0.69 | 0.44 |
| 0.25 | 0.65 | 0.39 |

Robust range: λ ∈ [0.12, 0.18] maintains correlation >0.70

**SM6.3 DATA SOURCE SENSITIVITY**

Network Database Alternatives:

STRING Score Thresholds:

- Score >600: AUC 0.85 (850K edges)
- Score >700: AUC 0.88 (500K edges, current)
- Score >800: AUC 0.87 (280K edges)
- Score >900: AUC 0.83 (120K edges)

Conclusion: 700 threshold optimal

*Alternative PPI Databases*:

- BioGRID: AUC 0.84, ρ=0.91 vs. STRING
- HIPPIE: AUC 0.86, ρ=0.93
- IntAct: AUC 0.83, ρ=0.89

Conclusion: STRING optimal, results consistent across databases

Expression Platform:

RNA-seq (TCGA) vs. Microarray (GEO):

- Correlation of T-DRI scores: ρ=0.88 (p<0.001)
- Top 100 genes overlap: 87/100

Conclusion: Platform-independent for high-scoring genes

**SM6.4 COHORT VARIATIONS**

Leave-One-Out Tumor Type Analysis:

| Excluded Type | ΔTD-NV (mean) | Correlation |
| --- | --- | --- |
| Melanoma | -0.02 | ρ = 0.99 |
| APL | -0.01 | ρ = 0.99 |
| Breast | -0.03 | ρ = 0.98 |
| GBM | -0.02 | ρ = 0.99 |
| CML | -0.01 | ρ = 0.99 |
| All others | <0.03 | ρ = 0.97 |

Conclusion: Results not driven by single tumor type

**SM6.5 METHODOLOGICAL VARIATIONS**

Aggregation Method:

| **Method** | **T-DRI AUC** | **TD-NV AUC** |
| --- | --- | --- |
| Geometric mean (current) | **0.92** | **0.88** |
| Arithmetic mean | 0.87 | 0.82 |

Conclusion: Geometric mean enforces multi-dimensional requirement

Survival Analysis:

| **Method** | **C-index** | **Parameter Estimates** |
| --- | --- | --- |
| Cox regression (current) | 0.83 | Reference |
| Weibull AFT | 0.82 | Similar |
| Random survival forest | 0.84 | Consistent variable importance |

Conclusion: Results robust to model choice

**SM6.6 CLASSIFICATION STABILITY**

Bootstrap Validation (1,000 iterations)

| **Classification** | **Agreement** | **Cohen's κ** |
| --- | --- | --- |
| RDP-Class | 94% | 0.92 (excellent) |
| NV-Class | 91% | 0.89 (excellent) |
| CEP-Class | 89% | 0.85 (good) |
| MP-Class | 93% | 0.91 (excellent) |

5-Fold Cross-Validation:

| **Outcome** | **Mean AUC** | **SD** | **Range** |
| --- | --- | --- | --- |
| 5-year OS | 0.86 | 0.03 | 0.83-0.89 |
| Metastasis | 0.84 | 0.04 | 0.79-0.87 |
| Response | 0.81 | 0.05 | 0.76-0.86 |

Conclusion: Consistent performance across folds

**SM6.7 THRESHOLD SENSITIVITY**

TD-NV Classification Thresholds (±0.05, ±0.10):

Current: NV-A >0.85, NV-B 0.5-0.8, NV-C <0.5

| **Variation** | **Clinical HR (NV-B vs. NV-C)** | **Stability** |
| --- | --- | --- |
| Current | 2.3 | Reference |
| ±0.05 | 2.1-2.5 | Stable |
| ±0.10 | 1.9-2.7 | More variable |

Conclusion: Robust within ±0.05 threshold variation

CEP-Class Thresholds:

Current: CEP-I >0.7, CEP-II 0.4-0.7, CEP-III <0.4

| **Variation** | **Early Response Rate** | **Late Resistance** | **Reclassification** |
| --- | --- | --- | --- |
| Current | 72% / 48% / 31% | 68% / 42% / 18% | Reference |
| ±0.05 | 68-76% / 44-52% / 28-34% | 64-72% / 38-46% / 15-21% | 7% patients |
| ±0.10 | 62-81% / 38-58% / 24-38% | 58-78% / 32-52% / 12-24% | 15% patients |

MP-Class Thresholds:

Current: MP-I >0.65 invasion score, MP-II 0.35-0.65, MP-III <0.35

| **Variation** | **Metastasis Prediction AUC** | **5-Year MFS HR** | **Stability** |
| --- | --- | --- | --- |
| Current | 0.842 | MP-I vs MP-III: 5.4 | Reference |
| ±0.05 | 0.834-0.848 | 4.9-5.9 | Good |
| ±0.10 | 0.816-0.861 | 4.2-6.8 | Moderate |

Conclusion: All DNV-TC classification thresholds robust within ±0.05 variation.

CEP and MP classes show similar stability to NV-Class, supporting clinical applicability of current cutoffs.

**SM6.8 MISSING DATA IMPACT**

Simulated Missingness:

| **% Missing** | **Imputation vs. Complete** | **Classification Agreement** |
| --- | --- | --- |
| 10% | ρ = 0.97 | 95% |
| 25% | ρ = 0.91 | 88% |
| 50% | ρ = 0.79 | 76% |

Conclusion: Robust to ≤25% missingness with imputation

**SM6.9 OVERALL ROBUSTNESS SUMMARY**

Highly Robust (minimal impact):

- Component weights ±10%: ρ>0.94
- Database choice: ρ>0.90
- Tumor type exclusion: ρ>0.97
- Classification thresholds ±0.05: HR within 90%

Moderately Robust (some impact):

- Aggregation method: ΔAUC≈0.05
- Simulation method (Boolean vs. ODE): ρ=0.84
- Missing data 25%: ρ=0.91

Requires Careful Selection:

- Network completeness (>50% edge loss): ρ<0.80
- Temporal decay λ outside [0.12, 0.18]: ρ<0.70

Overall Conclusion: DNV-TC classifications robust to reasonable variations in parameters, data sources, and analytical methods, supporting clinical reliability.

SM6.10 OVERFITTING AND CIRCULARITY ASSESSMENT

"A potential concern in framework development is circular validation — using the same datasets for both parameter establishment and performance evaluation. DNV-TC addresses this through three design principles: (1) all component weights were established a priori from biological literature rather than optimized on study data; (2) classification thresholds were defined from first principles (e.g., NV-A threshold of TD-NV >0.85 reflects the empirical boundary between oncogene-addicted and heterogeneous tumors established in prior oncology literature); (3) concordance with clinical outcomes was assessed against published survival and response data from independent analyses (Hoadley et al., 2018; Chapman et al., 2011), not data used in network construction. Nevertheless, we acknowledge that true prospective validation on independent cohorts is required to exclude residual circularity, as discussed in the main text (Section 4.3)."

**SM6.11 REFERENCES**

Efron, B., and Tibshirani, R.J. (1993). An Introduction to the Bootstrap. Chapman & Hall/CRC.

Hastie, T., et al. (2009). The Elements of Statistical Learning, 2nd ed. Springer.

**SUPPLEMENTARY TABLES**

**TABLE S1**. **T-DRI SCORES FOR TOP 50 GENES ACROSS 12 CANCER TYPES**

**UNIVERSAL GENES (High across all cancers)**

**Gene	GBM	Mela-	Lum	CML	TNBC PDAC Neuro MBL ccRCC CRC	SCLC	APL	Mean	SD**

**noma	A**

TP53	0.82	0.78	0.71	0.68	0.85	0.89	0.75	0.72	0.74	0.81	0.83	0.79	0.78	0.06

MYC	0.75	0.68	0.62	0.59	0.81	0.78	0.69	0.71	0.64	0.77	0.79	0.74	0.71	0.07

EGFR	0.88	0.67	0.52	0.48	0.71	0.64	0.58	0.62	0.56	0.69	0.74	0.51	0.63	0.12

PIK3CA 0.72	0.69	0.78	0.54	0.76	0.71	0.58	0.61	0.63	0.81	0.68	0.62	0.68	0.08

PTEN	0.79	0.68	0.71	0.52	0.74	0.69	0.61	0.64	0.58	0.72	0.66	0.55	0.66	0.08

AKT1	0.68	0.64	0.69	0.58	0.72	0.71	0.59	0.61	0.62	0.74	0.67	0.61	0.66	0.05

CDKN2A 0.74	0.81	0.58	0.62	0.69	0.85	0.68	0.71	0.64	0.72	0.78	0.67	0.71	0.08

RB1	0.71	0.64	0.68	0.59	0.72	0.67	0.78	0.74	0.61	0.69	0.82	0.64	0.69	0.07

NOTCH1 0.64	0.58	0.61	0.71	0.68	0.62	0.59	0.67	0.54	0.66	0.72	0.88	0.66	0.09

CTNNB1 0.68	0.62	0.59	0.54	0.64	0.71	0.58	0.82	0.61	0.85	0.63	0.57	0.65	0.10

**LINEAGE-SPECIFIC GENES**

SOX2	0.91	0.15	0.08	0.05	0.12	0.09	0.18	0.21	0.07	0.11	0.88	0.09	0.25	0.32

SOX1	0 0.08	0.87	0.12	0.05	0.09	0.07	0.72	0.11	0.06	0.08	0.14	0.07	0.20	0.29

GATA3	0.06	0.08	0.88	0.11	0.24	0.09	0.07	0.08	0.12	0.10	0.09	0.15	0.17	0.24

VHL	0.11	0.09	0.08	0.07	0.12	0.10	0.08	0.09	0.94	0.11	0.10	0.08	0.16	0.25

BCR-ABL0.00	0.00	0.00	0.98	0.00	0.00	0.00	0.00	0.00	0.00	0.00	0.00	0.08	0.28

PML-RARA 0.00	0.00	0.00	0.00	0.00	0.00	0.00	0.00	0.00	0.00	0.00	0.97	0.08	0.28

ESR1	0.08	0.06	0.82	0.09	0.18	0.07	0.05	0.06	0.11	0.09	0.07	0.12	0.15	0.23

FOXA1	0.09	0.07	0.71	0.11	0.22	0.14	0.06	0.08	0.13	0.16	0.12	0.10	0.17	0.19

OLIG2	0.78	0.11	0.06	0.05	0.09	0.08	0.12	0.14	0.07	0.09	0.18	0.06	0.15	0.23

ASCL1	0.21	0.08	0.05	0.04	0.07	0.19	0.28	0.11	0.06	0.08	0.91	0.05	0.18	0.27

MYCN	0.28	0.19	0.11	0.08	0.14	0.16	0.92	0.24	0.09	0.12	0.31	0.10	0.23	0.26

GATA1	0.05	0.04	0.07	0.78	0.06	0.05	0.08	0.06	0.09	0.07	0.06	0.84	0.19	0.30

GATA2	0.07	0.06	0.09	0.71	0.08	0.07	0.11	0.08	0.10	0.09	0.08	0.68	0.19	0.25

RUNX1	0.09	0.08	0.12	0.82	0.11	0.09	0.14	0.10	0.13	0.11	0.10	0.79	0.22	0.28

**ONCOGENES**

BRAF	0.25	0.89	0.18	0.15	0.22	0.19	0.21	0.28	0.16	0.68	0.24	0.17	0.30	0.23

KRAS	0.18	0.21	0.24	0.16	0.27	0.94	0.19	0.22	0.15	0.75	0.72	0.18	0.35	0.28

NRAS	0.32	0.71	0.28	0.42	0.31	0.29	0.24	0.27	0.22	0.38	0.34	0.48	0.36	0.14

MET	0.78	0.48	0.39	0.28	0.52	0.64	0.41	0.38	0.71	0.45	0.59	0.32	0.50	0.16

ALK	0.24	0.19	0.16	0.21	0.18	0.22	0.81	0.17	0.14	0.19	0.67	0.15	0.28	0.22

ERBB2	0.42	0.38	0.74	0.28	0.48	0.52	0.31	0.34	0.36	0.58	0.41	0.29	0.43	0.13

RET	0.18	0.22	0.19	0.16	0.21	0.58	0.24	0.21	0.17	0.28	0.31	0.18	0.24	0.11

FGFR2	0.51	0.34	0.62	0.24	0.48	0.41	0.28	0.38	0.32	0.54	0.36	0.26	0.40	0.12

IDH1	0.85	0.12	0.08	0.42	0.11	0.14	0.09	0.11	0.10	0.21	0.13	0.64	0.25	0.27

**TUMOR SUPPRESSORS**

NF1	0.72	0.58	0.48	0.42	0.61	0.54	0.68	0.52	0.49	0.56	0.64	0.45	0.56	0.09

APC	0.21	0.18	0.24	0.19	0.28	0.32	0.17	0.22	0.20	0.91	0.26	0.21	0.28	0.21

BRCA1	0.38	0.42	0.68	0.31	0.74	0.48	0.34	0.36	0.41	0.52	0.44	0.38	0.45	0.13

BRCA2	0.41	0.44	0.71	0.34	0.69	0.52	0.36	0.38	0.43	0.54	0.46	0.39	0.47	0.12

STK11	0.28	0.32	0.38	0.24	0.41	0.48	0.26	0.29	0.31	0.42	0.78	0.28	0.37	0.15

**EMT & INVASION GENES**

TWIST1	0.42	0.68	0.21	0.14	0.86	0.81	0.38	0.34	0.28	0.52	0.48	0.18	0.44	0.23

ZEB1	0.38	0.61	0.18	0.12	0.82	0.78	0.34	0.31	0.24	0.48	0.44	0.16	0.41	0.23

SNAI1	0.41	0.64	0.22	0.15	0.79	0.74	0.36	0.32	0.26	0.51	0.46	0.17	0.42	0.22

VIM	0.48	0.71	0.28	0.21	0.84	0.82	0.42	0.38	0.34	0.58	0.52	0.24	0.49	0.22

CDH1	0.32	0.24	0.68	0.28	0.19	0.22	0.21	0.26	0.34	0.42	0.28	0.31	0.31	0.14

**EPIGENETIC REGULATORS**

EZH2	0.71	0.64	0.58	0.48	0.74	0.68	0.62	0.78	0.54	0.66	0.69	0.52	0.64	0.09

DNMT3A	0.42	0.38	0.34	0.81	0.41	0.38	0.36	0.39	0.37	0.44	0.40	0.88	0.46	0.18

TET2	0.38	0.34	0.31	0.74	0.36	0.35	0.33	0.36	0.34	0.41	0.37	0.82	0.43	0.17

KMT2A	0.51	0.46	0.42	0.78	0.48	0.44	0.54	0.49	0.41	0.52	0.56	0.84	0.54	0.14

ARID1A	0.64	0.58	0.68	0.42	0.71	0.62	0.48	0.52	0.74	0.78	0.61	0.46	0.60

Abbreviations: GBM, glioblastoma; Lum A, luminal A breast cancer; CML, chronic myeloid leukemia; TNBC, triple-negative breast cancer; PDAC, pancreatic ductal adenocarcinoma; Neuro, neuroblastoma; MBL, medulloblastoma; ccRCC, clear cell renal cell carcinoma; CRC, colorectal cancer; SCLC, small cell lung cancer; APL, acute promyelocytic leukemia; Mean, average T-DRI score across all 12 cancer types; SD, standard deviation.

Gene Categories and Patterns:

Universal Genes (n=10): TP53, MYC, EGFR, PIK3CA, PTEN, AKT1, CDKN2A, RB1, NOTCH1, CTNNB1 show consistently high scores (mean >0.60) across all cancer types, reflecting fundamental roles in tumor biology regardless of lineage origin.

Lineage-Specific Genes (n=14): Extreme tissue specificity with high scores in cognate lineages and near-zero elsewhere. Examples: SOX2 (GBM 0.91, SCLC 0.88; neuroectoderm), SOX10 (melanoma 0.87, neuroblastoma 0.72; neural crest), GATA3 (luminal breast 0.88; mammary epithelium), VHL (ccRCC 0.94; kidney), ASCL1 (SCLC 0.91; neuroendocrine), MYCN (neuroblastoma 0.92; sympathetic nervous system), GATA1/GATA2/RUNX1 (leukemias 0.68-0.84; hematopoietic lineages).

Oncofusion Proteins (n=2): BCR-ABL (CML 0.98) and PML-RARA (APL 0.97) show absolute lineage restriction (all other cancer types 0.00), reflecting unique defining genetic dependencies in specific leukemia subtypes.

Oncogenes (n=9): Tissue-preferential activation patterns. BRAF (melanoma 0.89, CRC 0.68), KRAS (PDAC 0.94, CRC 0.75, SCLC 0.72; endodermal cancers), NRAS (melanoma 0.71; broader distribution), MET (GBM 0.78, ccRCC 0.71; RTK amplification), ALK (neuroblastoma 0.81, SCLC 0.67), IDH1 (GBM 0.85, AML 0.64; IDH1-mutant contexts).

Tumor Suppressors (n=5): Moderate scores reflecting partial redundancy and context-dependent functions. APC elevated in CRC (0.91), BRCA1/2 in breast cancers (0.68-0.74), STK11 in SCLC (0.78).

EMT Genes (n=5): TWIST1, ZEB1, SNAI1, VIM coordinately elevated in aggressive mesenchymal cancers—TNBC (0.79-0.86) and PDAC (0.74-0.82)—consistent with high metastatic propensity. CDH1 shows inverse pattern (luminal breast 0.68, epithelial contexts).

Epigenetic Regulators (n=5): EZH2 broadly elevated (mean 0.64), DNMT3A/TET2/KMT2A preferentially high in leukemias (0.74-0.88), ARID1A in epithelial cancers with chromatin remodeling dependencies (ccRCC 0.74, CRC 0.78).

Data Sources: T-DRI scores calculated from TCGA Pan-Cancer Atlas (n=4,827 patients, 12 cancer types). T-DRI from developmental databases (MGI, ENCODE, UCSC PhyloP), β from gnomAD v2.1.1, network components from STRING v11.5 and tumor-specific expression. Validation with DepMap CRISPR screens (ρ=0.62, p<0.001) and clinical targeted therapy response (ρ=0.78, p<0.001). Complete validation in Supplementary Methods Section S2.

Complete T-DRI scores for all genes and all tumor types are available from the corresponding author upon request.

**TABLE S2. TD-NV VALIDATION BENCHMARKS AND REPRESENTATIVE ONCOGENE DEPENDENCIES**

| **Gene** | **Cancer** | **TD-NV** | **Clinical agent** | **Reported response rate** |
| --- | --- | --- | --- | --- |
| BCR-ABL | CML | 0.98 | Imatinib | 98% |
| PML-RARA | APL | 0.97 | ATRA + arsenic | 95% |
| VHL | ccRCC | 0.94 | Anti-VEGF | 58% |
| MYCN | Neuroblastoma | 0.92 | No agent | N/A |
| ALK | ALCL/NSCLC | 0.91 | Crizotinib | 65-75% |
| BRAF | Melanoma | 0.89 | Vemurafenib | 48% |
| KIT | GIST | 0.88 | Imatinib | 83% |
| EGFR | Lung adenocarcinoma | 0.87 | Erlotinib | 71% |

Validation summary: 24/25 established dependencies achieved TD-NV >0.80 (mean 0.89 ± 0.05). In 712 cancer cell lines, TD-NV correlated with CERES dependency scores (Spearman ρ = 0.62; AUC = 0.88). In 156 NV-A patients, TD-NV correlated with targeted-therapy response (Spearman ρ = 0.78, p < 0.001).

**TABLE S3: CLINICAL OUTCOMES BY DNV-TC SIGNATURE**

**PANEL A: OUTCOMES BY RDP-CLASS**

| RDP_  Class | Description | N  Patients | Median  OS_  months | 5yr_OS  percent | 95pct  CI_  lower | 95pct  CI_  upper | Median  PFS_  months | 1yr_  PFS  percent | Metastatic  Events | Metastatic_  Rate  percent | Median_Time  to_Mets_months |
| --- | --- | --- | --- | --- | --- | --- | --- | --- | --- | --- | --- |

RDP-I	Pluripotency/Stemness	145	31.2	52	44	60	8.7	38	84	58	16.4

RDP-II	Neural Crest	178	28.5	48	41	55	7.2	34	112	63	14.8

RDP-III EM Transition	192	22.7	38	31	45	5.8	28	150	78	12.3

RDP-IV Morphogenic Pathways 134	38.6	64	56	72	12.4	52	56	42	24.7

RDP-V Lineage Commitment	217	54.3	71	65	77	18.9	68	61	28	38.5

Pairwise comparisons (Holm-corrected):

RDP-III vs RDP-V: HR 2.14 (95% CI 1.66-2.76), p<0.001

RDP-I vs RDP-V: HR 1.68 (1.28-2.21), p<0.001

RDP-II vs RDP-V: HR 1.78 (1.36-2.33), p<0.001

**PANEL B: OUTCOMES BY NV-CLASS (with CEP integration)**

| NV  Class | Description | N  Patients | Response  Rate  percent | 95pct  CI_  lower | 95pct  CI_  upper | Median  TTR  weeks | TTR_  range  weeks | Median  DOR  months | DOR_  range_  months | 5yr OS  percent | 95pct  CI  lower | 95pct  CI_  upper | Median_OS_  months |
| --- | --- | --- | --- | --- | --- | --- | --- | --- | --- | --- | --- | --- | --- |

NV-A	High Single-Node	156	87	81	92	4.2	2.8-6.1	18.5	12.3-28.7	68	61	75	42.0

Vulnerability

NV-B	Distributed	423	45	40	50	7.8	4.2-12.4	6.8	5.2-8.4	42	37	47	25.0

Vulnerability

NV-C	Low	287	35	29	41	16.2	8.7-24.3	28.4	22.7-36.8	78	73	83	72.0

Vulnerability

Treatment modality effects (NV-A only):

Targeted monotherapy: Response 91%, median PFS 22.4 months, OS 58.7 months (HR 0.28 vs untreated)

Combination (target+chemo): Response 94%, median PFS 28.8 months, OS 64.2 months (HR 0.21)

NV×CEP interaction: Within NV-A, cascade phenotype modulates duration (CEP-I: 12.3mo, CEP-III: 28.4mo) but not response rate (78-89%). Within NV-C, cascade affects both response (28-42%) and duration (18.3-32.7mo).

**PANEL C: OUTCOMES BY CEP-CLASS**

| CEP  Class | Description | N  Patients | Early_  Response_Rate_  percent | Early  Response_CI_  lower | Early_  Response  CI_  upper | Late_  ResistanceRate_  percent | Late_  ResistanceCI_  lower | Late_  Resistance  CI_  upper | Median  OS_  months | 5yr_OS_  percent | Median_  PFS  months |
| --- | --- | --- | --- | --- | --- | --- | --- | --- | --- | --- | --- |

CEP-I	Hyper-Cascading	387	72	67	77	68	63	73	29.4	45	7.8

CEP-II	Moderate Cascading	312	48	42	54	42	36	48	42.7	58	11.6

CEP-III	Low Cascading	167	31	24	38	18	12	24	58.2	72	16.8

**PANEL D: OUTCOMES BY MP-CLASS (with ROC metrics)**

| MP  Class | Description | N  Patients | Metastatic  Events | Metastatic  Rate  percent | Median  Time_  to_  Mets_  months | Time_  to  Mets  Range  months | 5yr_  MFS  percent | 95pct  CI_  lower | 95pct  CI_  upper | Distant  Sites_  mean | Median  PFS  months | Lung_  mets percent | Brain  mets  percent | Bone  mets_  percent |
| --- | --- | --- | --- | --- | --- | --- | --- | --- | --- | --- | --- | --- | --- | --- |

MP-I	High Metastatic	245	191	78	14.2	8.4-21.6	22	16	28	2.8	68	82	45	58

MP-II Intermediate	389	167	42.9	28.4	18.7-42.3	57	51	63	1.4	38	52	24	31

MP-III Low	198	36	18.2	62.8	48.3-84.5	82	76	88	0.8	18	24	8	14

Metastasis Prediction Performance:

MP-Class: AUC 0.842 (95% CI 0.801-0.883), Sensitivity 78%, Specificity 75%

TNM Staging: AUC 0.712 (0.665-0.759), Sensitivity 65%, Specificity 65%

Combined 4D DNV-TC: AUC 0.878 (0.842-0.914), Sensitivity 82%, Specificity 79%

DeLong test MP vs TNM: p<0.001

**PANEL E: COMBINED SIGNATURES (TOP 10 MOST COMMON)**

| DNV_Signature | N  Patients | Primary_  Examples | Response  Rate_  percent | Median_TTR_weeks | Median_DOR  months | Median_OS | 5yr_OS_percent | Metastatic_  Rate_  percent | Distant  Median_Time  to_Mets_months |
| --- | --- | --- | --- | --- | --- | --- | --- | --- | --- |

RDP-I/NV-B/CEP-I/MP-II	89	GBM	42	6.8	8.4	15.3	8	52 1	8.7

RDP-II/NV-B/CEP-I/MP-I	67	Melanoma BRAF	68	4.8	7.2	48.2	48	78	14.2

RDP-II/NV-A/CEP-I/MP-II	34	Neuroblastoma MYCN 82	3.2	18.6	67.8	55	48	22.4

RDP-III/NV-B/CEP-I/MP-I	78	TNBC	38	8.4	6.1	28.4	42	76	12.8

RDP-III/NV-B/CEP-I/MP-I	56	PDAC	22	9.2	5.2	12.7	8	89	9.4

RDP-V/NV-A/CEP-I/MP-II	43	CML BCR-ABL	98	2.8	120	120	89	12	NA

RDP-V/NV-A/CEP-I/MP-II	38	APL PML-RARA	95	3.1	120	120	85	8	NA

RDP-V/NV-C/CEP-II/MP-III	156	Luminal A Breast	35	14.6	32.4	92.3	82 1	8	54.8

RDP-V/NV-A/CEP-II/MP-II	92	ccRCC VHL	58	8.2	24.7	78.4	71	34	28.5

RDP-IV/NV-A/CEP-I/MP-II	48	Medulloblastoma SHH 71	5.4	36.8	84.7	65	28	32.6

Key observations:

Single-node vulnerabilities (NV-A) with oncofusions achieve >95% response: CML, APL

Distributed vulnerabilities (NV-B) with EMT programs show worst outcomes: TNBC, PDAC

Lineage commitment (RDP-V) with low cascade (CEP-II/III) predicts excellent survival: Luminal A breast

**PANEL F: RESPONSE PATTERNS BY NV-CLASS × CEP-CLASS**

| **NV-Class** | **CEP-Class** | **N patients** | **Overall response rate (%)** | **Median DOR (months)** |
| --- | --- | --- | --- | --- |

NV-A	CEP-I	89	89	12.3

NV-A	CEP-II	45	85	18.7

NV-A	CEP-III	22	78	28.4

NV-B	CEP-I	245	52	6.2

NV-B	CEP-II	128	41	8.9

NV-B	CEP-III	50	38	12.1

NV-C	CEP-I	147	42	18.3

NV-C	CEP-II	58	32	24.6

NV-C	CEP-III	82	28	32.7

**PANEL G: SURVIVAL BY TREATMENT MODALITY (NV-CLASS)**

| NV_  Class | Treatment | N_  Patients | Response_  Rate_  percent | Median_  PFS_  months | Median_  OS_  months | 5yr_  OS_  percent | HR_vs_  no_  treatment | HR_  95pct_CI  lower | HR_  95pct_CI  upper | p_value |
| --- | --- | --- | --- | --- | --- | --- | --- | --- | --- | --- |

NV-A	Targeted Monotherapy	98	91	22.4	58.7	72	0.28	0.19	0.42	<0.001

NV-A	Chemotherapy	42	48	8.6	32.4	54	0.64	0.42	0.98	0.039

NV-A	Combination

(Target+Chemo)	16	94	28.8	64.2	78	0.21	0.11	0.39	<0.001

NV-B	Targeted Monotherapy	156	52	7.2	28.4	38	0.71	0.58	0.87	0.001

NV-B	Chemotherapy	187	42	8.4	32.6	42	0.68	0.56	0.83	<0.001

NV-B	Target+Chemo	80	68	14.8	48.3	58	0.42	0.31	0.57	<0.001

NV-C	Targeted Monotherapy	84	38	18.6	68.4	76	0.58	0.42	0.79	<0.001

NV-C	Chemotherapy	142	32	14.2	58.7	72	0.62	0.48	0.81	<0.001

NV-C	Hormonal Therapy	61	42	32.7	98.6	84	0.34	0.22	0.53	<0.001

**PANEL H: ROC ANALYSIS FOR METASTASIS PREDICTION**

| Predictor | AUC t | 95pct_  CI_  lower | 95pct_  CI_  upper | Sensitivity  percent | Specificity percent | PPV_  percent | NPV  percent | p_value_vs_TNM |
| --- | --- | --- | --- | --- | --- | --- | --- | --- |

MP-Class (DNV-	0.842	0.801	0.883	78	75	72	81	<0.001

TNM Anatomic Staging	0.712	0.665	0.759	65	65	58	71	Reference

RDP-Class	0.768	0.724	0.812	71	68	64	75	0.024

NV-Class	0.621	0.572	0.670	58	62	54	66	0.148

CEP-Class	0.687	0.638	0.736	64	66	60	70	0.082

Combined DNV-TC (4D)	0.878	0.842	0.914	82	79	76	84	<0.001

**PANEL I: MULTIVARIATE COX REGRESSION (OVERALL SURVIVAL)**

| Variable | Reference | Comparison | Hazard_  Ratio | HR_95pct  CI_  lower | HR_95pct  CI_  upper | P  value | Coefficient | SE |
| --- | --- | --- | --- | --- | --- | --- | --- | --- |

NV-Class	NV-C (Low)	NV-A (Single-node)	1.42	1.08	1.86	0.012	0.35	0.14

NV-Class	NV-C (Low)	NV-B (Distributed)	2.31	1.84	2.89	<0.001	0.84	0.12

RDP-Class	RDP-V (Lineage) RDP-I (Pluripotency)	1.68	1.28	2.21	<0.001	0.52	0.14

RDP-Class	RDP-V (Lineage) RDP-II (Neural Crest)	1.78	1.36	2.33	<0.001	0.58	0.14

RDP-Class	RDP-V (Lineage) RDP-III (EMT)	2.14	1.66	2.76	<0.001	0.76	0.13

RDP-Class	RDP-V (Lineage) RDP-IV (Morphogenic)	1.24	0.92	1.67	0.156	0.22	0.15

CEP-Class	CEP-III (Low)	CEP-I (Hyper)	1.58	1.22	2.04	<0.001	0.46	0.13

CEP-Class	CEP-III (Low)	CEP-II (Moderate)	1.28	0.98	1.67	0.069	0.25	0.14

MP-Class	MP-III (Low)	MP-I (High)	1.94	1.48	2.54	<0.001	0.66	0.14

MP-Class	MP-III (Low)	MP-II (Intermediate)	1.42	1.11	1.82	0.006	0.35	0.13

TNM	Stage I/II	III	1.86	1.52	2.28	<0.001	0.62	0.10

TNM	StageI/II	IV	3.24	2.64	3.98	<0.001	1.18	0.10

Grade	1-2	3-4	1.52	1.28	1.81	0.001	0.42	0.09

Age	(per 10 years)	Continuous	1.18	1.12	1.25	<0.001	0.17	0.03

Interpretation: All four DNV-TC dimensions (RDP, NV, CEP, MP) provide independent prognostic information after adjusting for conventional clinicopathological variables. DNV-TC contributes C-index +0.18 beyond stage/grade/age.

**PANEL J: PROGRESSION-FREE SURVIVAL BY DNV-TC CLASS (Kaplan-Meier)**

| **Classification** | **Class** | **N patients** | **Median PFS (months)** | **1-year PFS (%)** |
| --- | --- | --- | --- | --- |

RDP-Class	RDP-I	145	8.7	38

RDP-Class	RDP-II	178	7.2	34

RDP-Class	RDP-III	192	5.8	28

RDP-Class	RDP-IV	134	12.4	52

RDP-Class	RDP-V	217	18.9	68

NV-Class	NV-A	156	28.4	68.3

NV-Class	NV-B	423	14.2	48.7

NV-Class	NV-C	287	54.7	79.8

CEP-Class	CEP-I	387	8.4	36

CEP-Class	CEP-II	312	12.8	52

CEP-Class	CEP-III	167	18.2	72

MP-Class	MP-I	245	7.8	32

MP-Class	MP-II	389	14.2	54

**TABLE S3 FOOTNOTES (CONSOLIDATED):**

Comprehensive clinical outcomes for 866 patients across 12 cancer types. Panel A: Reactivated Developmental Program (RDP) classes show survival gradient from RDP-V (differentiated, median OS 54.3 months) to RDP-III (EMT, 22.7 months). Panel B: Network Vulnerability (NV) classes integrated with Cascade Expansion Phenotype (CEP) demonstrate response patterns and resistance kinetics. NV-A achieves 87% response but shows bimodal duration (16-39 months depending on CEP). Panel D: Metastatic Propensity (MP) classes predict metastatic events with superior performance vs. TNM staging (AUC 0.842 vs 0.712, p<0.001). Panel E: Combined four-dimensional signatures illustrate clinical heterogeneity—single-node dependencies (BCR-ABL, PML-RARA) achieve >95% response while distributed EMT programs (TNBC, PDAC) show <25% response despite therapy. Panel I: Multivariate analysis confirms independent prognostic value of all DNV-TC dimensions.

Abbreviations: RDP, Reactivated Developmental Program; NV, Network Vulnerability; CEP, Cascade Expansion Phenotype; MP, Metastatic Propensity; OS, overall survival; PFS, progression-free survival; MFS, metastasis-free survival; TTR, time to response; DOR, duration of response; HR, hazard ratio; CI, confidence interval; AUC, area under curve; GBM, glioblastoma; TNBC, triple-negative breast cancer; PDAC, pancreatic ductal adenocarcinoma; CML, chronic myeloid leukemia; APL, acute promyelocytic leukemia; ccRCC, clear cell renal cell carcinoma.

Statistical Methods: Overall and progression-free survival estimated by Kaplan-Meier method, compared by log-rank test. Multivariate analysis via Cox proportional hazards regression. Response rates compared by chi-square or Fisher's exact test. ROC curves compared by DeLong test. All p-values adjusted for multiple comparisons using Benjamini-Hochberg FDR correction (α<0.05). See Supplementary Methods Section S5 for complete statistical methodology.

Data Sources: TCGA Pan-Cancer Atlas (Hoadley et al. 2018), supplemented with published cohort data. Median follow-up 48.7 months (range 2.1-156.8). Response assessed by RECIST 1.1 (solid tumors) or IWG criteria (leukemias). Treatment reflects standard-of-care 2012-2020. Total: 866 patients, 478 deaths (55%) and 588 progression events (68%). Metastatic follow-up was available for 832 patients, with 394 metastatic events (47.4%); 34 patients lacked metastatic follow-up data.

Progression-free survival summary (Panel J): Median and 1-year progression-free survival are reported for the DNV-TC classes listed in Panel J. For NV-class, these values are aligned with Figure 3B of the main article. The corresponding overall survival curves by NV-class are shown in Figure 5 of the main article (reproduced here as Supplementary Figure S3).

**TABLE S4: VALIDATION DATASETS AND DATA SOURCES FOR DNV-TC FRAMEWORK**

CLINICAL COHORTS

Dataset 1: TCGA Pan-Cancer Atlas

Source: NCI Genomic Data Commons (GDC) | Accession: TCGA-PANCAN | URL: https://portal.gdc.cancer.gov | Sample Size: 4,827 patients (12 cancer types) | Data Types: RNA-seq, whole exome sequencing, DNA methylation (450K), copy number variation, clinical outcomes | Median Follow-up: 48.7 months | Primary Use: Primary cohort for DNV-TC classification development and validation | Reference: Hoadley et al. (2018) Cell 173:291-304

Dataset 2: TCGA-GBM (Glioblastoma)

Source: The Cancer Genome Atlas | Accession: TCGA-GBM | URL: https://portal.gdc.cancer.gov/projects/TCGA-GBM | Sample Size: 617 patients | Data Types: RNA-seq, whole exome sequencing, DNA methylation, copy number variation, clinical outcomes | Median Follow-up: 14.2 months | Primary Use: GBM classification, SOX2/EGFR network analysis, RDP-I validation | Reference: Brennan et al. (2013) Cell 155:462-477

Dataset 3: TCGA-SKCM (Melanoma)

Source: The Cancer Genome Atlas | Accession: TCGA-SKCM | URL: https://portal.gdc.cancer.gov/projects/TCGA-SKCM | Sample Size: 470 patients | Data Types: RNA-seq, whole exome sequencing, DNA methylation, copy number variation, clinical outcomes | Median Follow-up: 52.3 months | Primary Use: BRAF V600E mutation analysis, SOX10/neural crest program validation, NV-B distributed vulnerability | Reference: Cancer Genome Atlas Network (2015) Cell 161:1681-1696

Dataset 4: TCGA-BRCA (Breast Cancer)

Source: The Cancer Genome Atlas | Accession: TCGA-BRCA | URL: https://portal.gdc.cancer.gov/projects/TCGA-BRCA | Sample Size: 1,100 patients | Data Types: RNA-seq, whole exome sequencing, DNA methylation, copy number variation, clinical outcomes, PAM50 subtypes | Median Follow-up: 68.4 months | Primary Use: Luminal A (GATA3/ESR1, NV-C) vs TNBC (TWIST1/ZEB1, NV-B) comparison | Reference: Cancer Genome Atlas Network (2012) Nature 490:61-70

Dataset 5: TCGA-LAML (Acute Myeloid Leukemia)

Source: The Cancer Genome Atlas | Accession: TCGA-LAML | URL: https://portal.gdc.cancer.gov/projects/TCGA-LAML | Sample Size: 200 patients (including APL) | Data Types: RNA-seq, whole exome sequencing, clinical outcomes, cytogenetics | Median Follow-up: 24.8 months | Primary Use: APL PML-RARA fusion analysis, NV-A single-node vulnerability validation | Reference: Cancer Genome Atlas Research Network (2013) N Engl J Med 368:2059-2074

Dataset 6: TARGET-NBL (Neuroblastoma)

Source: Therapeutically Applicable Research to Generate Effective Treatments (TARGET) | Accession: TARGET-NBL | URL: https://target-data.nci.nih.gov/Public/NBL | Sample Size: 249 patients (pediatric) | Data Types: RNA-seq, whole exome sequencing, copy number variation, clinical outcomes, MYCN amplification status | Median Follow-up: 42.6 months | Primary Use: MYCN amplification analysis, neural crest lineage (RDP-II), NV-A validation in pediatric cancer | Reference: Pugh et al. (2013) Nature 497:338-343

Dataset 7: BEAT AML

Source: Oregon Health & Science University (OHSU) | Accession: phs001657 | URL: https://www.ncbi.nlm.nih.gov/projects/gap/cgi-bin/study.cgi?study_id=phs001657 | Sample Size: 562 patients | Data Types: RNA-seq, whole exome sequencing, ex vivo drug sensitivity testing (122 drugs) | Primary Use: NV-A therapeutic response validation in patient-derived AML samples | Reference: Tyner et al. (2018) Nature 562:526-531

FUNCTIONAL GENOMICS VALIDATION

Dataset 8: Cancer Cell Line Encyclopedia (CCLE)

Source: Broad Institute | Accession: CCLE | URL: https://sites.broadinstitute.org/ccle | Sample Size: 712 cancer cell lines (34 cancer types) | Data Types: RNA-seq, whole exome sequencing, CRISPR dependency screens (Avana library), drug response profiles | Primary Use: Orthogonal validation of TD-NV gene rankings via CRISPR dependency scores (CERES). TD-NV vs CERES correlation: ρ=0.62, p<0.001; AUC=0.88 | Reference: Ghandi et al. (2019) Nature 569:503-508

Dataset 9: DepMap Public 23Q4

Source: Broad Institute Cancer Dependency Map | Accession: DepMap-23Q4 | URL: https://depmap.org/portal | Sample Size: 1,073 cell lines (multiple cancer types) | Data Types: RNA-seq, CRISPR screens (Avana/KY libraries), drug screening, proteomics | Primary Use: TD-NV vs CERES dependency correlation analysis, systematic vulnerability validation across cancer types | Reference: Tsherniak et al. (2017) Cell 170:564-576

Dataset 10: Perturb-seq (Dixit et al.)

Source: Broad Institute / MIT | Accession: GSE90063 | URL: https://www.ncbi.nlm.nih.gov/geo/query/acc.cgi?acc=GSE90063 | Sample Size: ~200,000 single cells across 24 gene perturbations (K562 CML cells) | Data Types: Single-cell RNA-seq combined with CRISPR-mediated gene knockdown | Primary Use: CEI-T cascade expansion validation—direct measurement of transcriptional cascades following gene perturbation. CEI-T vs observed DEGs correlation: ρ=0.72, p<0.001 | Reference: Dixit et al. (2016) Cell 167:1853-1866

Dataset 11: LINCS L1000

Source: NIH Library of Integrated Network-based Cellular Signatures (LINCS) Program | Accession: LINCS | URL: https://lincsproject.org | Sample Size: 978 cell lines, >1.3 million expression profiles | Data Types: Transcriptional response profiles to 42,080 perturbagens (small molecules, genetic perturbations) with time-course data | Primary Use: Temporal decay parameter (λ) optimization by correlating predicted cascade dynamics with observed 6h/24h/48h time-course responses. Optimal λ=0.15 (r=0.68) | Reference: Subramanian et al. (2017) Cell 171:1437-1452

NETWORK CONSTRUCTION DATABASES

Dataset 12: STRING v11.5

Source: STRING Consortium | Accession: STRING-v11.5 | URL: https://string-db.org | Sample Size: 19,576 human proteins, 11,759,454 protein-protein interactions | Data Types: Protein-protein interactions from experimental data, databases, text mining, and co-expression (combined score >0.7 threshold for high confidence) | Primary Use: Multi-layer network construction—signaling layer edges, physical protein interactions | Reference: Szklarczyk et al. (2019) Nucleic Acids Res 47:D607-D613

Dataset 13: ENCODE ChIP-seq Compendium

Source: Encyclopedia of DNA Elements (ENCODE) Consortium | Accession: ENCODE | URL: https://www.encodeproject.org | Sample Size: 1,394 transcription factors, >50,000 ChIP-seq experiments across multiple cell types | Data Types: ChIP-seq peak calls for transcription factor binding sites (q-value <0.01 threshold) | Primary Use: Transcriptional layer construction—transcription factor-target gene regulatory relationships | Reference: ENCODE Project Consortium (2012) Nature 489:57-74

Dataset 14: GTEx v8

Source: Genotype-Tissue Expression (GTEx) Project, NIH Common Fund | Accession: GTEx-v8 | URL: https://gtexportal.org | Sample Size: 948 donors, 17,382 tissue samples (54 tissue types) | Data Types: RNA-seq expression profiles from non-diseased individuals | Primary Use: Normal tissue network construction for network rewiring analysis (ρ̃ component)—tumor vs normal network comparison | Reference: GTEx Consortium (2020) Science 369:1318-1330

EVOLUTIONARY AND DEVELOPMENTAL DATABASES

Dataset 15: gnomAD v2.1.1

Source: Genome Aggregation Database (gnomAD), Broad Institute | Accession: gnomAD-v2.1.1 | URL: https://gnomad.broadinstitute.org | Sample Size: 141,456 non-diseased individuals | Data Types: Allele frequencies, loss-of-function constraint metrics (pLI, LOEUF scores for 19,704 genes) | Primary Use: Evolutionary constraint calculation for β component of TD-NV—genes under strong negative selection (high pLI, low LOEUF) indicate non-redundancy and essentiality | Reference: Karczewski et al. (2020) Nature 581:434-443

Dataset 16: Mouse Genome Informatics (MGI)

Source: The Jackson Laboratory | Accession: MGI | URL: http://www.informatics.jax.org | Sample Size: >78,000 genes with >850,000 phenotype annotations | Data Types: Mouse knockout phenotypes annotated using Mammalian Phenotype Ontology (MPO) | Primary Use: Developmental essentiality assessment for DRI_dev component—genes with embryonic lethal (MP:0011110) or severe developmental defects (MP:0001672, MP:0002084) phenotypes | Reference: Smith et al. (2019) Nucleic Acids Res 47:D801-D806

FOOTNOTES

Sixteen primary datasets used for DNV-TC development, validation, and clinical correlation, organized into four categories: Clinical Cohorts (n=7,625 patients total), Functional Genomics Validation (>2,800 cell lines, >1.5M perturbation profiles), Network Construction Databases (19,576 proteins, 1,394 TFs), and Evolutionary/Developmental Databases (141,456 individuals, >78,000 genes).

Clinical Cohorts (Datasets 1-7): TCGA Pan-Cancer Atlas (n=4,827) served as primary development cohort with median follow-up 48.7 months. Individual tumor type cohorts provided cancer-specific validation (GBM, melanoma, breast, leukemia, neuroblastoma). BEAT AML (n=562) enabled ex vivo drug response validation.

Functional Genomics (Datasets 8-11): CCLE (n=712) and DepMap (n=1,073) provided CRISPR dependency validation (TD-NV vs CERES ρ=0.62, AUC=0.88). Perturb-seq single-cell screens validated CEI-T predictions (ρ=0.72). LINCS L1000 (>1.3M profiles) optimized temporal decay λ=0.15.

Network Databases (Datasets 12-14): STRING v11.5 (11.7M interactions) provided signaling/PPI layer. ENCODE (>50K ChIP-seq experiments) defined TF-target relationships. GTEx v8 (17,382 normal samples) enabled tumor-normal rewiring analysis.

Evolutionary/Developmental (Datasets 15-16): gnomAD v2.1.1 (141,456 individuals) quantified constraint (pLI/LOEUF for β). MGI (>850K phenotypes) provided developmental essentiality for DRI_dev.

Data Access: TCGA/TARGET via NCI GDC portal (https://portal.gdc.cancer.gov, controlled access via dbGaP for raw data). DepMap via https://depmap.org (open). STRING, ENCODE, gnomAD, MGI, GTEx freely accessible. GEO datasets (GSE90063, phs001657) require dbGaP authorization. Preprocessed data and analysis scripts are available from the corresponding author upon request.

Abbreviations: WES, whole exome sequencing; CNV, copy number variation; CRISPR, CRISPR-Cas9 screens; scRNA-seq, single-cell RNA sequencing; PPI, protein-protein interactions; TF, transcription factor; pLI, probability of loss-of-function intolerance; LOEUF, loss-of-function observed/expected upper bound; MPO, Mammalian Phenotype Ontology.

Complete dataset descriptions, file formats, preprocessing pipelines, and access protocols documented in Supplementary Methods Section S4.

**TABLE S5. SUMMARY OF SENSITIVITY AND ROBUSTNESS ANALYSES**

| **Analysis** | **Variation / comparison** | **Result** | **Interpretation** |
| --- | --- | --- | --- |
| T-DRI component weights | Alternative weighting schemes | AUC 0.87-0.89; rank ρ 0.91-0.96 vs baseline | Rankings stable |
| TD-NV component weights | Alternative weighting schemes | AUC 0.84-0.86; rank ρ 0.94-0.97 vs baseline | Stable across weighting schemes |
| CEI-T temporal decay | λ 0.12-0.18 | Validation correlation >0.70 | Robust operating range |
| Alternative PPI databases | BioGRID / HIPPIE / IntAct | AUC 0.83-0.86; ρ 0.89-0.93 vs STRING | Database choice has limited impact |
| Leave-one-out tumor type | Each tumor type excluded in turn | ΔTD-NV <0.03; ρ ≥0.97 | Not driven by a single tumor type |
| Bootstrap classification | 1,000 iterations | Agreement 89-94%; Cohen κ 0.85-0.92 | Good-to-excellent stability |
| 5-fold cross-validation | OS / metastasis / response | Mean AUC 0.86 / 0.84 / 0.81 | Consistent predictive performance |
| Threshold sensitivity | ±0.05 around current thresholds | NV HR 2.1-2.5; MP AUC 0.834-0.848; CEP 7% reclassification | Robust within ±0.05 |
| Missing-data sensitivity | 25% simulated missingness with imputation | ρ 0.91; 88% classification agreement | Robust to moderate missingness |

Source: Supplementary Methods Section S6. Values reproduce the sensitivity, cross-validation, threshold and missing-data analyses reported there.

**TABLE S6. DNV-TC SIGNATURE GENE SETS AND CLASSIFICATION THRESHOLDS**

| **Dimension / class** | **Signature genes or defining criteria** |
| --- | --- |
| RDP-I — Pluripotency | SOX2, OCT4, NANOG, MYC, KLF4; GSEA NES >2.0, FDR <0.05 |
| RDP-II — Neural crest | SOX10, PAX3, MITF, MYCN; GSEA NES >2.0, FDR <0.05 |
| RDP-III — EMT | TWIST1, SNAI1, ZEB1, VIM, CDH2; GSEA NES >2.0, FDR <0.05 |
| RDP-IV — Morphogenic | SHH, GLI1, WNT3A, CTNNB1, FGF2; pathway activation >0.6 |
| RDP-V — Lineage commitment | GATA3, NOTCH1, RUNX1, ESR1, FOXA1; GSEA NES >2.0, FDR <0.05 |
| NV-A — High single-node vulnerability | ≥1 gene with TD-NV >0.85; Gini coefficient >0.6 |
| NV-B — Distributed vulnerability | Multiple genes with TD-NV 0.5-0.8; Gini coefficient <0.5 |
| NV-C — Low vulnerability | Few genes with TD-NV >0.5; stable differentiated architecture |
| CEP-I — Hyper-cascading | Mean CEI-T of top 10 TD-NV genes >0.7 |
| CEP-II — Moderate cascading | Mean CEI-T 0.4-0.7 |
| CEP-III — Low cascading | Mean CEI-T <0.4 |
| MP-I — High metastatic propensity | RDP-II or RDP-III active; invasion score >0.65 |
| MP-II — Intermediate metastatic propensity | Mixed features; invasion score 0.35-0.65 |
| MP-III — Low metastatic propensity | RDP-V predominant; invasion score <0.35 |
| MP invasion gene set | MMP2, MMP9, MMP14, TWIST1, SNAI1, ZEB1, ITGA5, ITGAV, ITGB1, ITGB3 |

Source: DNV-TC classification criteria in Table 1 and the accompanying classification footnote of the main article. These are the signature genes and thresholds used for class assignment in the submitted framework.

**Supplementary Figures**

**Supplementary Figure S1. DNV-TC four-dimensional tumor classification system (full-page version of Figure 1 of the main article).** Comprehensive framework integrating four orthogonal classification dimensions. RDP-Class (Reactivated Developmental Program): Five developmental programs ranked by differentiation state—Pluripotency (RDP-I, SOX2/OCT4/NANOG), Neural Crest (RDP-II, SOX10/MITF/PAX3), EMT Transition (RDP-III, TWIST1/SNAI1/ZEB1), Morphogenic Pathways (RDP-IV, CTNNB1/SHH/WNT), Lineage Commitment (RDP-V, GATA3/NOTCH1/RUNX1).

NV-Class (Network Vulnerability): Three vulnerability architectures based on TD-NV scores—Single Vulnerability (NV-A, TD-NV >0.85, one dominant dependency node), Distributed Vulnerability (NV-B, TD-NV 0.5-0.8, multiple intermediate nodes), Low Vulnerability (NV-C, TD-NV <0.5, stable dispersed network).

CEP-Class (Cascade Expansion Phenotype): Perturbation propagation dynamics from CEI-T modeling—High Cascade (CEP-I, CEI-T >0.7, rapid network reorganization), Moderate Cascade (CEP-II, 0.4-0.7), Low Cascade (CEP-III, <0.4, limited propagation).

MP-Class (Metastatic Propensity): Invasion and metastatic potential—High (MP-I, 78% metastatic rate at median 14 months), Medium (MP-II, 43% at 28 months), Low (MP-III, 18% at >60 months). Body diagrams show representative metastatic burden.

Example Integration: BRAF V600E melanoma classified as RDP-II (neural crest origin) / NV-B (distributed MAPK pathway vulnerabilities) / CEP-I (high cascade following BRAF inhibition) / MP-I (high metastatic propensity), predicting intermediate targeted therapy response (68%) with frequent resistance emergence.

**Supplementary Figure S2. T-DRI scores across cancer types (full-page version of Figure 2 of the main article).** Heatmap showing Tumor Developmental Regulatory Impact (T-DRI) scores for 50 representative genes across 12 cancer types. Color intensity indicates T-DRI magnitude (blue = 0.0 minimal, red = 1.0 extreme). Genes grouped into three categories: Universal (high T-DRI across all cancer types, n=10 genes including TP53, MYC, EGFR), Lineage-Specific (tissue-restricted high T-DRI, including SOX10 in melanoma, GATA3 in luminal breast, BCR-ABL in CML, and PML-RARA in APL), and Other genes with generally lower T-DRI. Universal genes show consistently elevated T-DRI (mean >0.60) reflecting fundamental roles in tumor biology. Lineage-specific genes display extreme tissue restriction with "hot spots" corresponding to developmental lineage: SOX10/MITF (neural crest → melanoma), GATA3/ESR1 (mammary epithelium → breast), SOX2/OLIG2 (neuroectoderm → GBM/SCLC), MYCN (sympathetic neurons → neuroblastoma), VHL (kidney → ccRCC), BCR-ABL (CML), PML-RARA (APL), GATA1/RUNX1 (hematopoietic → leukemias). Complete T-DRI scores for all genes in Table S1.

Abbreviations: GBM, glioblastoma; Lum A, luminal A breast cancer; CML, chronic myeloid leukemia; TNBC, triple-negative breast cancer; PDAC, pancreatic ductal adenocarcinoma; Neuro, neuroblastoma; MBL, medulloblastoma; ccRCC, clear cell renal cell carcinoma; CRC, colorectal cancer; SCLC, small cell lung cancer; APL, acute promyelocytic leukemia.

**Supplementary Figure S3. Overall survival by network vulnerability class (full-page version of Figure 5 of the main article).** Kaplan-Meier survival curves for 866 patients stratified by Network Vulnerability (NV) Class. Patients were classified into three groups based on TD-NV scores and network topology analysis: NV-C (Low vulnerability, n=287, green), characterized by stable network architecture with few high-scoring vulnerability nodes (TD-NV <0.5 for most nodes); NV-A (Single-node vulnerable, n=156, red), characterized by dependence on one or few dominant vulnerability nodes (TD-NV >0.85 for key driver); NV-B (Distributed vulnerable, n=423, blue), characterized by multiple intermediate-scoring vulnerability nodes creating diffuse, untargetable dependencies (multiple nodes with TD-NV 0.5-0.8).

Solid lines represent Kaplan-Meier survival probability estimates; shaded regions indicate 95% confidence intervals. Vertical tick marks on curves denote censored observations. Median overall survival (time at which 50% of patients remained alive): NV-C 72 months, NV-A 42 months, NV-B 25 months. Five-year overall survival rates: NV-C 78%, NV-A 68%, NV-B 42%. The number-at-risk table shows patients remaining under observation at each time point.

Statistical comparison by log-rank test demonstrated highly significant differences in survival among the three groups (p<0.001). Pairwise comparisons (Holm-corrected): NV-C vs NV-B (HR 4.2, 95% CI 3.1-5.7, p<0.001), NV-C vs NV-A (HR 2.3, 95% CI 1.6-3.2, p<0.001), NV-A vs NV-B (HR 1.8, 95% CI 1.3-2.5, p<0.001). Multivariate Cox regression adjusting for stage, grade, and age confirmed independent prognostic value of NV-Class (see Table S3 Panel I).

Network Vulnerability Class demonstrates clear survival stratification independent of conventional clinical variables. Low vulnerability architecture (NV-C) predicts excellent long-term survival, consistent with stable, differentiated tumor biology. Single-node vulnerability (NV-A) shows intermediate prognosis with targetable dependencies. Distributed vulnerability (NV-B) confers poorest outcomes, reflecting untargetable multi-node dependencies characteristic of aggressive, undifferentiated malignancies.

Data sources: TCGA Pan-Cancer Atlas (n=4,827 primary cohort), supplemented with published cohort data. Median follow-up 48.7 months (range 2.1-156.8). Overall survival defined as time from diagnosis to death from any cause. Complete methodology in Supplementary Methods Sections S2-S5.

Abbreviations: NV, Network Vulnerability; TD-NV, Tumor Disease Network Vulnerability; HR, hazard ratio; CI, confidence interval.

**Supplementary Figure S4. Clinical outcomes stratified by DNV-TC classes (full-page version of Figure 3 of the main article).** Error bars indicate 95% confidence intervals.

In Panel C, metastatic follow-up data were missing for 34 patients and were excluded from metastatic rate calculations.

ORR denotes complete plus partial response.

Abbreviations: NV, network vulnerability; CEP, cascade expansion phenotype; ORR, overall response rate; DOR, duration of response.

**Supplementary Figure S5. Multi-layer tumor network architecture (full-page version of Figure 7 of the main article).** Schematic representation of the multi-layer tumor regulatory network architecture. The network is organized into four hierarchical layers: the signaling layer (top), transcriptional layer, epigenetic layer, and metabolic layer (bottom). Nodes represent genes or proteins within each regulatory layer, while edges indicate interactions within layers. Vertical arrows denote information flow and regulatory coupling between layers. Key hub genes with high tumor developmental network vulnerability (High TD-NV), including EGFR, TP53, MYC, DNMT, and TET, are highlighted by enlarged nodes with red outlines. The diagram illustrates how perturbations in upstream signaling propagate through transcriptional and epigenetic programs to influence downstream metabolic states, emphasizing multi-level integration in tumor regulatory networks.

**Supplementary Figure S6. Therapeutic response by network vulnerability class (full-page version of Figure 4 of the main article).** Therapeutic response characteristics stratified by Network Vulnerability (NV) class.

(A) Overall response rate (ORR) shown as stacked horizontal bars, partitioned into complete response (CR), partial response (PR), and no response. ORR represents the sum of CR and PR.

(B) Median time to response across NV classes, with error bars indicating variability in response onset.

(C) Median duration of response, highlighting distinct response durability patterns, including bimodal response in NV-A, rapid resistance in NV-B, and durable responses in NV-C. A horizontal dashed line denotes the 24-month durability benchmark.

**Supplementary Figure S7. ROC curves for 5-year metastasis-free survival prediction (full-page version of Figure 6 of the main article).** Receiver operating characteristic (ROC) curve analysis comparing the predictive performance of the MP-Class derived from the DNV-TC framework and conventional TNM staging for 5-year metastasis-free survival. The MP-Class model (red curve) demonstrates superior discrimination compared with TNM staging (blue curve), with a higher area under the curve (AUC). The dashed diagonal line represents no discrimination (AUC = 0.50). Optimal cutoff points are indicated for each model, illustrating sensitivity–specificity trade-offs. Statistical comparison of AUCs was performed using the DeLong test.
