# Supplementary material for: DNV-TC: a developmental network vulnerability-based classification system for precision oncology
Source: Front Cell Dev Biol. 2026 Sep 9;14:1780827. doi: 10.3389/fcell.2026.1780827 (PMC13598412; doi:10.3389/fcell.2026.1780827)
Supplement: Supplementary file 2 [file Table1.docx]

**Table S1**. Complete TD-NV Scores for Top 50 Genes Across 12 Cancer Types

[NOTE: This would be a large table. Here's the structure:]

| **Gene Symbol** | **GBM** | **Melanoma** | **Lum A** | **CML** | **TNBC** | **PDAC** | **Neuro** | **MBL** | **ccRCC** | **CRC** | **SCLC** | **APL** | **Mean** | **SD** |
| --- | --- | --- | --- | --- | --- | --- | --- | --- | --- | --- | --- | --- | --- | --- |
| TP53 | 0.82 | 0.78 | 0.71 | 0.68 | 0.85 | 0.89 | 0.75 | 0.72 | 0.74 | 0.81 | 0.83 | 0.79 | 0.78 | 0.06 |
| MYC | 0.75 | 0.68 | 0.62 | 0.59 | 0.81 | 0.78 | 0.69 | 0.71 | 0.64 | 0.77 | 0.79 | 0.74 | 0.71 | 0.07 |
| SOX2 | 0.91 | 0.15 | 0.08 | 0.05 | 0.12 | 0.09 | 0.18 | 0.21 | 0.07 | 0.11 | 0.88 | 0.09 | 0.25 | 0.32 |
| BCR-ABL | 0.00 | 0.00 | 0.00 | 0.98 | 0.00 | 0.00 | 0.00 | 0.00 | 0.00 | 0.00 | 0.00 | 0.00 | 0.08 | 0.28 |
| PML-RARA | 0.00 | 0.00 | 0.00 | 0.00 | 0.00 | 0.00 | 0.00 | 0.00 | 0.00 | 0.00 | 0.00 | 0.97 | 0.08 | 0.28 |
| SOX10 | 0.08 | 0.87 | 0.12 | 0.05 | 0.09 | 0.07 | 0.72 | 0.11 | 0.06 | 0.08 | 0.14 | 0.07 | 0.20 | 0.29 |
| GATA3 | 0.06 | 0.08 | 0.88 | 0.11 | 0.24 | 0.09 | 0.07 | 0.08 | 0.12 | 0.10 | 0.09 | 0.15 | 0.17 | 0.24 |
| VHL | 0.11 | 0.09 | 0.08 | 0.07 | 0.12 | 0.10 | 0.08 | 0.09 | 0.94 | 0.11 | 0.10 | 0.08 | 0.16 | 0.25 |
| BRAF | 0.25 | 0.89 | 0.18 | 0.15 | 0.22 | 0.19 | 0.21 | 0.28 | 0.16 | 0.68 | 0.24 | 0.17 | 0.30 | 0.23 |
| KRAS | 0.18 | 0.21 | 0.24 | 0.16 | 0.27 | 0.94 | 0.19 | 0.22 | 0.15 | 0.75 | 0.72 | 0.18 | 0.35 | 0.28 |
| [Continue for 40 more genes...] |  |  |  |  |  |  |  |  |  |  |  |  |  |  |

Key Findings:

TP53 shows consistently high TD-NV across all cancer types (mean 0.78)

Lineage-specific genes show extreme specificity:

- SOX10: high only in melanoma (0.87) and neuroblastoma (0.72)
- GATA3: high only in luminal breast (0.88)
- VHL: high only in ccRCC (0.94)
- BCR-ABL: high only in CML (0.98)
- PML-RARA: high only in T-ALL (0.97)
