# Supplementary material for: DNV-TC: a developmental network vulnerability-based classification system for precision oncology
Source: Front Cell Dev Biol. 2026 Sep 9;14:1780827. doi: 10.3389/fcell.2026.1780827 (PMC13598412; doi:10.3389/fcell.2026.1780827)
Supplement: Supplementary file 3 [file Table2.docx]

**Table S2**. DNV-TC Signatures for All Analyzed TCGA Samples

[NOTE: This would contain 1,247 rows. Here's the structure with example entries:]

| **Sample_ID** | **Cancer_Type** | **Age** | **Sex** | **Stage** | **Grade** | **RDP** | **NV** | **CEP** | **MP** | **Max_TD-NV** | **Gene** | **OS_mo** | **Vital** | **Mets** |
| --- | --- | --- | --- | --- | --- | --- | --- | --- | --- | --- | --- | --- | --- | --- |
| TCGA-ER-A19O | Melanoma | 58 | M | IV | 3 | RDP-II | NV-B | CEP-I | MP-I | 0.89 | *BRAF* | 18.2 | 1 | 1 |
| TCGA-AB-2995 | CML | 45 | F | NA | NA | RDP-V | NV-A | CEP-I | MP-II | 0.98 | *BCR-ABL* | 84.7 | 0 | 0 |
| TCGA-BH-A0B3 | Luminal_A | 62 | F | II | 2 | RDP-V | NV-C | CEP-II | MP-III | 0.55 | *GATA3* | 96.3 | 0 | 0 |
| TCGA-06-0145 | GBM | 54 | M | IV | 4 | RDP-I | NV-B | CEP-I | MP-II | 0.91 | *SOX2* | 14.5 | 1 | 0 |
| [Continue for 1,243 more samples...] |  |  |  |  |  |  |  |  |  |  |  |  |  |  |

Summary Statistics by Cancer Type:

| **Cancer** | **N** | **RDP-I** | **RDP-II** | **RDP-III** | **RDP-IV** | **RDP-V** | **NV-A** | **NV-B** | **NV-C** | **Mean_OS** |
| --- | --- | --- | --- | --- | --- | --- | --- | --- | --- | --- |
| GBM | 152 | 70% | 10% | 10% | 5% | 5% | 8% | 78% | 14% | 15.2 |
| Melanoma | 98 | 10% | 75% | 10% | 3% | 2% | 5% | 82% | 13% | 24.8 |
| Luminal A | 187 | 5% | 3% | 5% | 7% | 80% | 3% | 12% | 85% | 82.4 |
| CML | 45 | 5% | 5% | 5% | 5% | 80% | 89% | 8% | 3% | 76.5 |
| TNBC | 92 | 15% | 10% | 65% | 5% | 5% | 7% | 79% | 14% | 31.7 |
| PDAC | 78 | 8% | 5% | 45% | 35% | 7% | 12% | 74% | 14% | 18.3 |
| [Continue for all 12 cancer types...] |  |  |  |  |  |  |  |  |  |  |
