# Supplementary material for: DNV-TC: a developmental network vulnerability-based classification system for precision oncology
Source: Front Cell Dev Biol. 2026 Sep 9;14:1780827. doi: 10.3389/fcell.2026.1780827 (PMC13598412; doi:10.3389/fcell.2026.1780827)
Supplement: Supplementary file 4 [file Table3.docx]

**Table S3**. RDP-Class Gene Signatures (Complete Lists)

RDP-I: Pluripotency/Stemness (78 genes)

| **Gene Symbol** | **Entrez ID** | **Description** | **Knockout Phenotype** | **Peak Expression Stage** |
| --- | --- | --- | --- | --- |
| SOX2 | 6657 | SRY-box transcription factor 2 | Embryonic lethal E3.5 | Blastocyst |
| POU5F1 (OCT4) | 5460 | POU class 5 homeobox 1 | Embryonic lethal E3.5 | Blastocyst |
| NANOG | 79923 | Nanog homeobox | Embryonic lethal E5.5 | Blastocyst |
| MYC | 4609 | MYC proto-oncogene | Embryonic lethal E10.5 | Blastocyst-Gastrulation |
| KLF4 | 9314 | Kruppel-like factor 4 | Viable, skin barrier defect | Blastocyst |
| LIN28A | 79727 | Lin-28 homolog A | Viable, growth defect | Blastocyst |
| LIN28B | 389421 | Lin-28 homolog B | Viable, delayed puberty | Blastocyst |
| UTF1 | 8433 | Undifferentiated embryonic cell TF 1 | Embryonic lethal | Blastocyst |
| SALL4 | 57167 | Spalt-like transcription factor 4 | Embryonic lethal E9.5 | Blastocyst-Gastrulation |
| [Continue for 69 more genes...] |  |  |  |  |

RDP-II: Neural Crest (65 genes)

| **Gene Symbol** | **Entrez ID** | **Description** | **Knockout Phenotype** | **Peak Expression Stage** |
| --- | --- | --- | --- | --- |
| SOX10 | 6663 | SRY-box transcription factor 10 | Embryonic lethal E11.5, neural crest defects | Neurulation |
| SOX9 | 6662 | SRY-box transcription factor 9 | Embryonic lethal E11.5, cartilage defects | Neurulation-Organogenesis |
| PAX3 | 5077 | Paired box gene 3 | Embryonic lethal E13.5, neural tube defects | Neurulation |
| PAX7 | 5081 | Paired box gene 7 | Viable, muscle defects | Neurulation |
| MITF | 4286 | Melanocyte inducing transcription factor | Viable, pigmentation defects | Neurulation-Organogenesis |
| MYCN | 4613 | MYCN proto-oncogene | Embryonic lethal E11.5, neural defects | Neurulation-Organogenesis |
| SNAI2 (SLUG) | 6591 | Snail family transcriptional repressor 2 | Viable, melanocyte defects | Neurulation |
| [Continue for 58 more genes...] |  |  |  |  |

RDP-III: Epithelial-Mesenchymal Transition (54 genes)

| **Gene Symbol** | **Entrez ID** | **Description** | **Developmental Role** | **Cancer Role** |
| --- | --- | --- | --- | --- |
| TWIST1 | 7291 | Twist family bHLH transcription factor 1 | Mesoderm development | EMT driver |
| TWIST2 | 117581 | Twist family bHLH transcription factor 2 | Mesoderm development | EMT driver |
| SNAI1 (SNAIL) | 6615 | Snail family transcriptional repressor 1 | EMT in gastrulation | EMT driver |
| SNAI2 (SLUG) | 6591 | Snail family transcriptional repressor 2 | Neural crest EMT | EMT driver |
| ZEB1 | 6935 | Zinc finger E-box binding homeobox 1 | EMT regulation | EMT driver |
| ZEB2 | 9839 | Zinc finger E-box binding homeobox 2 | EMT regulation | EMT driver |
| VIM (Vimentin) | 7431 | Vimentin | Mesenchymal marker | EMT marker |
| CDH2 (N-cadherin) | 1000 | Cadherin 2 | Neural/mesenchymal adhesion | EMT marker |
| [Continue for 46 more genes...] |  |  |  |  |

RDP-IV: Morphogenic Pathways (92 genes)

Hedgehog Pathway (14 genes):

SHH, IHH, DHH, PTCH1, PTCH2, SMO, GLI1, GLI2, GLI3, SUFU, GAS1, BOC, CDON, HHIP

Wnt Pathway (43 genes):

WNT1, WNT2, WNT3A, WNT4, WNT5A, WNT7A, WNT8A, WNT10B, CTNNB1, APC, AXIN1, AXIN2, GSK3B, TCF7, TCF7L1, TCF7L2, LEF1, LRP5, LRP6, FZD1, FZD2, FZD3, FZD4, FZD5, FZD6, FZD7, FZD8, FZD9, FZD10, DKK1, DKK2, DKK3, DKK4, SFRP1, SFRP2, SFRP3, SFRP4, SFRP5, [5 more]

FGF Pathway (14 genes):

FGF1, FGF2, FGF4, FGF8, FGF10, FGFR1, FGFR2, FGFR3, FGFR4, FGFRL1, SPRY1, SPRY2, SPRY3, SPRY4

BMP/TGFβ Pathway (21 genes):

BMP2, BMP4, BMP7, BMPR1A, BMPR1B, BMPR2, TGFB1, TGFB2, TGFB3, TGFBR1, TGFBR2, TGFBR3, SMAD1, SMAD2, SMAD3, SMAD4, SMAD5, SMAD6, SMAD7, SMAD8, SMAD9

RDP-V: Lineage Commitment (85 genes)

Hematopoietic Lineage (30 genes):

GATA1, GATA2, RUNX1, SPI1 (PU.1), CEBPA, CEBPB, GFI1, GFI1B, KLF1, TAL1, LMO2, NOTCH1, HES1, MYB, HOXA9, HOXA10, MEIS1, PBX1, [12 more]

Mammary Lineage (20 genes):

GATA3, FOXA1, ESR1 (ERα), PGR, XBP1, ELF5, RARA, KRT8, KRT18, KRT19, [10 more]

Neural Lineage (20 genes):

ASCL1, NEUROD1, NEUROG1, NEUROG2, OLIG1, OLIG2, NKX2-2, PAX6, SOX2, DCX, TUBB3, [9 more]

Muscle Lineage (15 genes):

MYOD1, MYOG, MYF5, MYF6, MEF2A, MEF2B, MEF2C, MEF2D, PAX3, PAX7, SIX1, [4 more]
