# Supplementary material for: DNV-TC: a developmental network vulnerability-based classification system for precision oncology
Source: Front Cell Dev Biol. 2026 Sep 9;14:1780827. doi: 10.3389/fcell.2026.1780827 (PMC13598412; doi:10.3389/fcell.2026.1780827)
Supplement: Supplementary file 5 [file Table4.docx]

**Table S4.** Statistical Details for All Survival Analyses

Overall Survival by NV-Class

| **NV-Class** | **N** | **Events** | **Deaths** | **Censored** | **Median OS (mo)** | **95% CI** | **1-yr OS** | **3-yr OS** | **5-yr OS** | **Log-rank χ²** | **p-value** |
| --- | --- | --- | --- | --- | --- | --- | --- | --- | --- | --- | --- |
| NV-A | 156 | 89 | 89 | 67 | 42.3 | 36.8-51.7 | 78.2% | 71.5% | 68.2% | 45.3 | <0.001 |
| NV-B | 423 | 287 | 287 | 136 | 24.7 | 22.1-28.4 | 62.4% | 48.9% | 42.1% | (ref) | (ref) |
| NV-C | 287 | 98 | 98 | 189 | 71.8 | 63.2-NR | 89.5% | 82.7% | 78.4% | 38.7 | <0.001 |

Paiwise Comparisons:

- NV-B vs NV-C: HR 2.31 (95% CI 1.85-2.88), p < 0.001
- NV-A vs NV-C: HR 1.52 (95% CI 1.18-1.95), p = 0.001
- NV-A vs NV-B: HR 0.66 (95% CI 0.52-0.84), p = 0.001

Multivariate Cox Regression Analysis

| **Variable** | **HR** | **95% CI** | **SE** | **z-value** | **p-value** |
| --- | --- | --- | --- | --- | --- |
| **NV-Class** | | | | | |
| NV-B vs NV-C | 2.28 | 1.81-2.87 | 0.12 | 8.95 | <0.001 |
| NV-A vs NV-C | 1.48 | 1.15-1.91 | 0.13 | 3.12 | 0.002 |
| **Stage** | | | | | |
| II vs I | 1.85 | 1.42-2.41 | 0.13 | 5.21 | <0.001 |
| III vs I | 3.12 | 2.45-3.98 | 0.12 | 10.45 | <0.001 |
| IV vs I | 8.47 | 6.52-11.01 | 0.13 | 18.92 | <0.001 |
| **Grade** | | | | | |
| 2 vs 1 | 1.32 | 1.08-1.61 | 0.10 | 2.78 | 0.005 |
|  |  |  |  |  |  |
| 3 vs 1 | 1.87 | 1.52-2.30 | 0.11 | 6.12 | <0.001 |
| **Age** (per 10 years) | 1.23 | 1.15-1.32 | 0.03 | 6.84 | <0.001 |

**Model Statistics:**

- Concordance index: 0.742 (95% CI 0.718-0.766)
- Likelihood ratio test: χ² = 385.6, df = 9, p < 0.001
- Wald test: χ² = 352.8, df = 9, p < 0.001
- Score test: χ² = 378.2, df = 9, p < 0.001

**Progression-Free Survival by NV-Class**

| **NV-Class** | **N** | **Events** | **Median PFS (mo)** | **95% CI** | **1-yr PFS** | **3-yr PFS** | **5-yr PFS** |
| --- | --- | --- | --- | --- | --- | --- | --- |
| NV-A | 156 | 102 | 28.4 | 23.7-38.6 | 68.3% | 52.7% | 48.2% |
| NV-B | 423 | 358 | 14.2 | 12.8-16.1 | 48.7% | 28.4% | 23.8% |
| NV-C | 287 | 145 | 54.7 | 46.8-NR | 79.8% | 68.2% | 62.5% |
