# Supplementary material for: DNV-TC: a developmental network vulnerability-based classification system for precision oncology
Source: Front Cell Dev Biol. 2026 Sep 9;14:1780827. doi: 10.3389/fcell.2026.1780827 (PMC13598412; doi:10.3389/fcell.2026.1780827)
Supplement: Supplementary file 6 [file Table5.docx]

**Table S5**. Metastatic Outcome Data by DNV-TC Classes

Metastatic Events by MP-Class

| **MP-Class** | **N** | **Mets** | **Rate (%)** | **95% CI** | **Median Time (mo)** | **95% CI** | **1-yr Mets-Free** | **3-yr Mets-Free** | **5-yr Mets-Free** |
| --- | --- | --- | --- | --- | --- | --- | --- | --- | --- |
| MP-I | 245 | 191 | 78.0 | 72.4-82.9 | 14.2 | 12.3-17.8 | 52.3% | 28.4% | 22.0% |
| MP-II | 389 | 167 | 42.9 | 38.1-47.9 | 28.4 | 24.7-34.5 | 71.8% | 60.2% | 57.1% |
| MP-III | 198 | 36 | 18.2 | 13.3-24.2 | 62.8 | 51.7-NR | 89.4% | 84.5% | 81.8% |

Log-rank test: χ² = 198.5, p < 0.001

Metastatic Sites by MP-Class

| **MP-Class** | **Brain (%)** | **Liver (%)** | **Lung (%)** | **Bone (%)** | **Multiple Sites (%)** |
| --- | --- | --- | --- | --- | --- |
| MP-I | 45.0 | 38.2 | 52.4 | 31.4 | 62.3 |
| MP-II | 28.1 | 28.4 | 24.0 | 19.2 | 31.1 |
| MP-III | 11.1 | 22.2 | 19.4 | 41.7 | 11.1 |

ROC Analysis for Metastasis Prediction

| **Predictor** | **AUC** | **95% CI** | **Sensitivity** | **Specificity** | **PPV** | **NPV** | **Accuracy** |
| --- | --- | --- | --- | --- | --- | --- | --- |
| MP-Class (DNV-TC) | 0.842 | 0.801-0.883 | 0.781 | 0.824 | 0.765 | 0.837 | 0.805 |
| TNM Stage | 0.712 | 0.665-0.759 | 0.652 | 0.701 | 0.621 | 0.728 | 0.679 |
| Histological Grade | 0.634 | 0.584-0.684 | 0.589 | 0.638 | 0.582 | 0.645 | 0.615 |
| Combined Model | 0.891 | 0.857-0.925 | 0.834 | 0.857 | 0.821 | 0.868 | 0.847 |

**DeLong test for AUC comparison:**

- MP-Class vs TNM: Δ AUC = 0.130 (95% CI 0.085-0.175), p < 0.001
- MP-Class vs Grade: Δ AUC = 0.208 (95% CI 0.158-0.258), p < 0.001
- Combined vs MP-Class: Δ AUC = 0.049 (95% CI 0.028-0.070), p < 0.001

Metastatic Pattern by RDP-Class

| **RDP-Class** | **N with Mets** | **Mets Rate (%)** | **Brain** | **Liver** | **Lung** | **Bone** | **Multiple** |
| --- | --- | --- | --- | --- | --- | --- | --- |
| RDP-I | 82 | 68.3 | 41% | 28% | 35% | 22% | 48% |
| RDP-II | 103 | 81.7 | 62% | 34% | 41% | 28% | 71% |
| RDP-III | 87 | 75.7 | 38% | 52% | 58% | 31% | 64% |
| RDP-IV | 45 | 57.0 | 24% | 38% | 31% | 35% | 38% |
| RDP-V | 77 | 36.7 | 18% | 31% | 24% | 47% | 29% |

**Key Observations:**

- Neural crest program (RDP-II) shows highest brain metastasis rate (62%), consistent with neurotropism
- EMT program (RDP-III) shows highest lung metastasis rate (58%), consistent with hematogenous dissemination
- Lineage commitment (RDP-V) shows highest bone metastasis rate (47%), consistent with homing to bone marrow niches
