# Supplementary material for: DNV-TC: a developmental network vulnerability-based classification system for precision oncology
Source: Front Cell Dev Biol. 2026 Sep 9;14:1780827. doi: 10.3389/fcell.2026.1780827 (PMC13598412; doi:10.3389/fcell.2026.1780827)
Supplement: Supplementary file 7 [file Table6.docx]

**Table S6.** Therapeutic Response Data by Tumor Type and DNV-TC Class

Melanoma (BRAF V600E, n=84)

| **Treatment** | **NV-Class** | **N** | **ORR (%)** | **95% CI** | **CR (%)** | **PR (%)** | **SD (%)** | **PD (%)** | **Median TTR (wk)** | **Median DOR (mo)** | **Median PFS (mo)** |
| --- | --- | --- | --- | --- | --- | --- | --- | --- | --- | --- | --- |
| Vemurafenib | NV-B | 52 | 61.5 | 47.0-74.7 | 7.7 | 53.8 | 23.1 | 15.4 | 6.2 | 6.8 | 5.3 |
| Dabrafenib | NV-B | 32 | 65.6 | 46.8-81.4 | 9.4 | 56.2 | 18.8 | 15.6 | 5.8 | 7.1 | 5.8 |
| Dab+Tram | NV-B | 41 | 73.2 | 57.1-85.8 | 14.6 | 58.5 | 19.5 | 7.3 | 5.1 | 11.4 | 11.0 |

Resistance Mechanisms Observed (n=38):

- NRAS mutations: 34.2%
- MEK1/2 mutations: 23.7%
- RTK upregulation (PDGFR, IGF1R): 26.3%
- PTEN loss: 18.4%
- Multiple mechanisms: 21.1%

Chronic Myeloid Leukemia (BCR-ABL+, n=45)

| **Treatment** | **NV-Class** | **N** | **CCyR (%)** | **MMR @ 12mo (%)** | **CMR @ 24mo (%)** | **Median TTR (wk)** | **OS @ 5yr (%)** |
| --- | --- | --- | --- | --- | --- | --- | --- |
| Imatinib 400mg | NV-A | 45 | 88.9 | 82.2 | 68.9 | 3.1 | 89.3 |

Resistance (n=5, 11.1%):

- T315I mutation: 3 patients (60%)
- E255K/V mutation: 1 patient (20%)
- F317L mutation: 1 patient (20%)

Second-line therapy outcomes:

- Dasatinib 100mg: 4/5 achieved CCyR (80%)
- Ponatinib 45mg: 1/1 T315I patient achieved CCyR (100%)

Luminal A Breast Cancer (ER+/HER2-, n=187)

| **Treatment** | **NV-Class** | **N** | **ORR (%)** | **CBR (%)** | **Median TTR (wk)** | **Median DOR (mo)** | **Median PFS (mo)** | **OS @ 5yr (%)** |
| --- | --- | --- | --- | --- | --- | --- | --- | --- |
| Tamoxifen | NV-C | 98 | 32.7 | 68.4 | 16.2 | 28.4 | 16.8 | 82.1 |
| Aromatase Inhibitor | NV-C | 89 | 38.2 | 72.8 | 14.7 | 32.6 | 18.3 | 84.7 |

Resistance Pattern:

- ESR1 mutations (D538G, Y537S): 18.7%
- Loss of ER expression: 12.3%
- PI3K/AKT pathway activation: 24.6%
- Unknown mechanism: 44.4%

Triple-Negative Breast Cancer (n=92)

| **Treatment** | **NV-Class** | **N** | **ORR (%)** | **Median PFS (mo)** | **Median OS (mo)** |
| --- | --- | --- | --- | --- | --- |
| Chemotherapy | NV-B | 65 | 41.2 | 7.2 | 18.3 |
| Chemo + Bevacizumab | NV-B | 27 | 10.8 | 10.8 | 22.7 |
| PARP-i (BRCA-deficient) | NV-A | 18 | 14.2 | 14.2 | NR |

Glioblastoma (IDH-WT, n=152)

| **Treatment** | **NV-Class** | **N** | **ORR (%)** | **6-mo PFS (%)** | **Median PFS (mo)** | **Median OS (mo)** |
| --- | --- | --- | --- | --- | --- | --- |
| TMZ + RT | NV-B | 89 | 22.5 | 53.9 | 6.9 | 14.8 |
| Bevacizumab | NV-B | 42 | 28.6 | 42.9 | 4.2 | 9.7 |
| Erlotinib | NV-B | 21 | 4.8 | 14.3 | 2.1 | 7.3 |

No single-agent targeted therapy showed significant benefit, consistent with distributed vulnerability.

Pancreatic Adenocarcinoma (n=78)
